# Atopic eczema in adulthood and mortality: UK population–based cohort study, 1998-2016

**DOI:** 10.1016/j.jaci.2020.12.001

**Published:** 2021-05

**Authors:** Richard J. Silverwood, Kathryn E. Mansfield, Amy Mulick, Angel Y.S. Wong, Sigrún A.J. Schmidt, Amanda Roberts, Liam Smeeth, Katrina Abuabara, Sinéad M. Langan

**Affiliations:** a Faculty of Epidemiology and Population Health, London School of Hygiene and Tropical Medicine, London, United Kingdom; b Centre for Longitudinal Studies, University College London Social Research Institute, University College London, London, United Kingdom; c Department of Clinical Epidemiology and Department of Dermatology, Aarhus University Hospital, Aarhus, Denmark; d Nottingham Support Group for Carers of Children with Eczema, Nottingham, United Kingdom; e Department of Dermatology, University of California, San Francisco School of Medicine, San Francisco, Calif; f Health Data Research UK, London, United Kingdom

**Keywords:** Activity, atopic eczema, cohort study, electronic health care records, mortality, population-based, primary care, severity, United Kingdom, CPRD, Clinical Practice Research Datalink, GP, General practitioner, HES, Hospital Episode Statistics, HR, Hazard ratio, ICD-10, *International Classification of Diseases*, 10th Revision, IMD, Index of Multiple Deprivation, ONS, Office for National Statistics

## Abstract

**Background:**

Atopic eczema affects up to 10% of adults and is becoming more common globally. Few studies have assessed whether atopic eczema increases the risk of death.

**Objective:**

We aimed to determine whether adults with atopic eczema were at increased risk of death overall and by specific causes and to assess whether the risk varied by atopic eczema severity and activity.

**Methods:**

The study was a population-based matched cohort study using UK primary care electronic health care records from the Clinical Practice Research Datalink with linked hospitalization data from Hospital Episode Statistics and mortality data from the Office for National Statistics from 1998 to 2016.

**Results:**

A total of 526,736 patients with atopic eczema were matched to 2,567,872 individuals without atopic eczema. The median age at entry was 41.8 years, and the median follow-up time was 4.5 years. There was limited evidence of increased hazard for all-cause mortality in those with atopic eczema (hazard ratio = 1.04; 99% CI = 1.03-1.06), but there were somewhat stronger associations (8%-14% increased hazard) for deaths due to infectious, digestive, and genitourinary causes. Differences on the absolute scale were modest owing to low overall mortality rates. Mortality risk increased markedly with eczema severity and activity. For example, patients with severe atopic eczema had a 62% increased hazard (hazard ratio = 1.62; 99% CI = 1.54-1.71) for mortality compared with those without eczema, with the strongest associations for infectious, respiratory, and genitourinary causes.

**Conclusion:**

The increased hazards for all-cause and cause-specific mortality were largely restricted to those with the most severe or predominantly active atopic eczema. Understanding the reasons for these increased hazards for mortality is an urgent priority.

Atopic eczema affects up to 10% of adults^1^ and is becoming more common globally.^2^ It is a challenging disease characterized by itch, sleeplessness, and adverse effects on quality of life. Approximately 30% of people with atopic eczema have moderate-to-severe disease.^3^, ^4^, ^5^

Recent evidence has led to a paradigm shift in how atopic eczema is perceived, from focusing on skin symptoms and associated allergic diseases to understanding that atopic eczema may be associated with a range of important medical outcomes. For example, we have previously examined the association between atopic eczema and cardiovascular outcomes, including cardiovascular death.^6^ A recent meta-analysis of the limited evidence base in this area that was conducted by our group found atopic eczema to be associated with an increased risk of myocardial infarction, ischemic stroke, angina, and heart failure in the identified cohort studies, although the analysis did not find any evidence for pooled associations with angina, myocardial infarction, heart failure, or stroke from cross-sectional studies.^7^

Few studies have assessed whether atopic eczema increases the risk of death. Danish data suggest that atopic eczema might be associated with a slightly increased risk of death in people hospitalized with atopic eczema and people diagnosed with atopic eczema in outpatient hospital speciality clinics.^8^^,^^9^ However, limited sample sizes and poor information on potential mediators hamper conclusions. Atopic eczema is a relapsing and remitting disease that varies in severity and disease activity, but no study has examined whether the potential association between atopic eczema and mortality differs in terms of these characteristics. If atopic eczema is associated with increased mortality, this is important from both a public health and a therapeutic perspective, as atopic eczema is common in adults.^1^

Therefore, our objective was to examine whether adults with atopic eczema had greater all-cause and cause-specific mortality rates than adults without atopic eczema in a UK general population cohort. We also aimed to investigate whether the all-cause and cause-specific mortality rates varied by atopic eczema severity and disease activity over time.

## Methods

### Study design and setting

We undertook a cohort study using data from the UK Clinical Practice Research Datalink (CPRD) that were linked to Hospital Episode Statistics (HES) inpatient data, Office for National Statistics (ONS) mortality data, and Index of Multiple Deprivation (IMD) data. CPRD is a database of prospectively collected primary care records from general practitioners (GPs) that were collected by using Vision software; approximately 7% of the UK population are represented in the database.^10^^,^^11^ Approximately 80% of CPRD practices registered in England have consented to their patients’ primary care records being linked to other data sources. HES records include all National Health Service–funded inpatient hospitalizations in England since 1997, including diagnoses and procedures. ONS-linked mortality data contain the underlying cause of death, recorded on the death certificate, along with up to 15 other recorded causes of death. IMD is an area-level measure of relative deprivation. For this study, data were extracted from the July 2017 CPRD build and the Set 14–linked data. All code lists are available on the London School of Hygiene and Tropical Medicine Data Compass website (https://doi.org/10.17037/DATA.00001986).

### Study population

All adults (aged ≥18 years) contributing to CPRD between January 2, 1998, and March 31, 2016, with linked HES and ONS mortality data were eligible for inclusion. All individuals had to be registered with a practice meeting CPRD quality control standards and have at least 12 months of registration before study entry (to allow adequate time for recording of covariate data in the patient record).

#### Defining patients with atopic eczema and matched unexposed individuals

The exposed cohort included all patients with atopic eczema, with onset defined at the latest of an atopic eczema diagnosis and 2 atopic eczema treatments (on separate dates), which is consistent with a validation study demonstrating a positive predictive value in adults of 82% (95% CI = 73%-89%)^12^ (see Methods E1 in the Online Repository at www.jacionline.org). For each patient with atopic eczema, we randomly matched without replacement up to 5 individuals without eczema by age (within 15 years), sex, and general practice (see Methods E1). A graphic depiction of the study design is presented in Fig E1 (in the Online Repository at www.jacionline.org).

### Defining follow-up

Follow-up for exposed patients began at the latest of the following dates: January 2, 1998; the date on which the individual turned 18 years old; the date on which the individual fulfilled our diagnostic algorithm for atopic eczema (ie, the latest of a record of a diagnostic morbidity code for eczema or the second record for eczema therapy), and the start of CPRD follow-up plus 365 days. Follow-up for unexposed individuals began at the start date of their matched patient with atopic eczema. Follow-up ended at the earliest of study end date, death (using the ONS date), transfer out of practice, or practice last collection date. Patients contributing at least 1 day of follow-up were included in the study.

### Defining eczema severity and activity

Atopic eczema severity was defined as a time-updated variable for patients with atopic eczema so that at any given point during follow-up they belonged to 1 of 3 severity categories: mild, moderate, or severe (see Methods E1). Atopic eczema activity was characterized by using the first 12 months of follow-up, with patients with atopic eczema split into 3 categories for analysis: those who never had active atopic eczema during the first year of follow-up, those who had active atopic eczema for less than 50% of the first year of follow-up, and those who had active atopic eczema for at least 50% of the first year of follow-up (see Methods E1). This was a *post hoc* analysis, as results from our prespecified analysis were suggestive of time-related bias (see Methods E2 in the Online Repository at www.jacionline.org).

### Defining mortality outcomes

Mortality was considered separately by underlying cause of death (infections [*International Classification of Diseases*, 10th Revision (ICD-10) Chapter I], neoplasms [ICD-10 Chapter II], circulatory disease [ICD-10 Chapter IX], respiratory disease [ICD-10 Chapter X], digestive disease [ICD-10 Chapter XI], diseases of the genitourinary system [ICD-10 Chapter XIV], and other causes [all other ICD-10 chapters]), as well as by all causes combined. Deaths from each cause were identified from ONS data, defined as any relevant ICD-10 (2001 onward) or *International Classification of Diseases*, Ninth Revision (before 2001) code recorded as a cause of death.

### Covariates

We used a directed acyclic graph^13^ to inform the identification of covariates and avoid collider bias (see Fig E2 in the Online Repository at www.jacionline.org). We considered variables relating to calendar period, sex, socioeconomic deprivation (IMD), ethnicity, and age as potential confounders to be adjusted for in analyses. Full details regarding the definition of these variables can be found in Methods E1.

### Statistical analysis

The characteristics of those with and without atopic eczema (at cohort entry) were described. We estimated the cause-specific cumulative incidence functions in the atopic eczema–*exposed* patients only on account of the fact that because of the matching, atopic eczema–*unexposed* patients in the sample are not representative of the population of eczema-unexposed patients. For each cause of death, we estimated the cumulative incidence function in the full sample of exposed patients nonparametrically, allowing for competing risks.^14^

#### Primary analyses

For both the all-cause and cause-specific mortality analyses, we used Cox regression stratified by matched set (matched on age at cohort entry, sex, and practice) with current age as the underlying time scale to estimate hazard ratios (HRs) for the association between atopic eczema and mortality (the “unadjusted” model). A subsequent multivariable analysis adjusted for *a priori* confounders (current calendar period, IMD, and time-varying asthma [the “adjusted” model]). We used 99% CIs and an implied 1% level of statistical significance to reduce the risk of type 1 error, consistent with CPRD Independent Scientific Advisory Committee guidelines.

All-cause and cause-specific mortality incidence rates in the atopic eczema–exposed patients were estimated by using the data in our sample. Incidence rates among people without atopic eczema (which cannot be reliably estimated from the sample on account of the matching) were then estimated by multiplying the incidence rate in the atopic eczema–exposed patients by our corresponding estimated HR (after having first inverted it so that it compared unexposed patients with exposed patients). Attributable risks were calculated as the difference between these exposure group–specific incidence rates. Population-attributable risks were estimated by using the estimated HR and assuming an atopic eczema prevalence of 10%.^15^

#### Secondary analyses

We repeated analyses (adjusted model only) within strata of sex, current asthma status, and current age group (18-39, 40-59, and ≥60 years) to explore potential effect modification. We also repeated the analysis with alternative exposure definitions, where atopic eczema was categorized on the basis of severity and separately, on the basis of category of disease activity. Finally, we conducted an exploratory mediation analysis that is described and reported in Methods E3 (in the Online Repository at www.jacionline.org).

#### Sensitivity analyses and model checking

A series of sensitivity analyses were conducted (Table I). We checked the proportional hazards assumption in all the primary analysis models through plots of the Schöenfeld residuals.

**Table I:** Overview of sensitivity analyses

Sensitivity analysis	Justification	Findings
*Primary analyses*		
The primary analysis was repeated on an incident (newly active) atopic eczema cohort (exposed patients, defined as those joining the cohort when they first fulfill our diagnostic criteria and after the start of the study period)	To reduce potential overadjustment, as covariates measured at entry precede atopic eczema onset in the incident atopic eczema cohort so will not be on the causal pathway between atopic eczema and mortality	Associations were partially attenuated for most causes of death relative to those in the primary analysis (see Table E6 in the Online Repository at www.jacionline.org)
The primary analysis was repeated on individuals with at least 1 consultation with their GP in the year before cohort entry	To reduce the possibility that the control group members are healthier purely because of the study design (controls are not required to have had a recent GP consultation in the primary analysis, whereas atopic eczema is defined on the basis of diagnosis and relevant treatment)	Associations were partially attenuated for all causes of death relative to those in the primary analysis (see Table E7 in the Online Repository at www.jacionline.org)
The primary analysis was repeated on a first redefined cohort (“first redefined cohort”), where the pool of unexposed persons also included patients with an atopic eczema diagnosis but without 2 further treatments for the entire duration of their follow-up and patients in the exposed cohort (with an atopic eczema diagnosis and 2 further treatments) were included as unexposed up until their cohort entry (ie, the latest of their atopic eczema diagnosis and their 2 further treatments)	To explore the sensitivity of the results to the definition of the exposure	Associations were very similar to those in the primary analysis for all causes of death (see Table E8 in the Online Repository at www.jacionline.org)
The primary analysis was repeated on a second redefined cohort (“second redefined cohort”), where the exposed patients were those with an atopic eczema diagnosis only (ie, without requiring 2 atopic eczema treatments), and these patients were eligible for the unexposed cohort up until their atopic eczema diagnosis (some patients may have had childhood atopic eczema but may not have had treatment codes recorded if registered at GP during adulthood, and therefore may have been erroneously excluded from the exposed cohort in the primary analysis)	To explore the sensitivity of the results to the definition of the exposure	Associations were partially attenuated for all causes of death relative to those in the primary analysis (see Table E9 in the Online Repository at www.jacionline.org)
The primary analysis was repeated on a subset of patients registered from 2006 onward, with additional adjustment for ethnicity	To examine whether the omission of this covariate in the primary analysis may have introduced bias	Associations adjusted for ethnicity (restricting to the 339,734 people who had such data available and who remained in valid matched sets) were consistent with those that were unadjusted for ethnicity in the same reduced cohort (see Table E10 in the Online Repository at www.jacionline.org)
The primary analysis was repeated with the exposure redefined to represent time-varying time since diagnosis (unexposed vs exposed at 0-4 years since diagnosis vs exposed at 5-9 years since diagnosis vs exposed at ≥10 years since diagnosis)	To examine whether the association between atopic eczema and all-cause and cause-specific mortality differed by time since diagnosis	The association with all-cause mortality appeared less strong within 5 years of diagnosis than it was more than 5 years after diagnosis, but the patterns with individual causes of death were heterogeneous (see Table E11 in the Online Repository at www.jacionline.org)
*Secondary analyses*		
The atopic eczema severity analysis was repeated on individuals with at least 1 consultation with their GP in the year before cohort entry	To reduce the possibility that the control group members were healthier purely owing to the study design (controls were not required to have had a recent GP consultation in the primary analysis, whereas atopic eczema was defined on the basis of diagnosis and relevant treatment)	Associations were only slightly attenuated relative to those in the unrestricted analysis (see Table E12 in the Online Repository at www.jacionline.org)
The atopic eczema activity analysis was repeated on individuals with at least 1 consultation with their GP in the year before cohort entry	To reduce the possibility that the control group was healthier purely on account of to the study design (controls were not required to have had a recent GP consultation in the primary analysis, whereas atopic eczema was defined on the basis of diagnosis and relevant treatment)	Associations were only slightly attenuated relative to those in the unrestricted analysis (see Table E13 in the Online Repository at www.jacionline.org)
The atopic eczema activity analysis (assessed during the first 12 months of follow-up, assuming active eczema for 3 months after a single health care contact) was repeated, without excluding the first year of follow-up	To explore any potential bias caused by conditioning on survival up to 12 months in the analysis in which the first 12 months of follow-up are excluded. However, bias due to inclusion of the exposure definition period in follow-up may instead be induced	Associations were generally very similar, although with greater evidence of a protective association with moderately active atopic eczema for some causes of death (see Table E14 in the Online Repository at www.jacionline.org)

#### Software

All analyses were conducted using Stata, version 15 (StataCorp, College Station, Tex).

## Results

**Fig 1:**
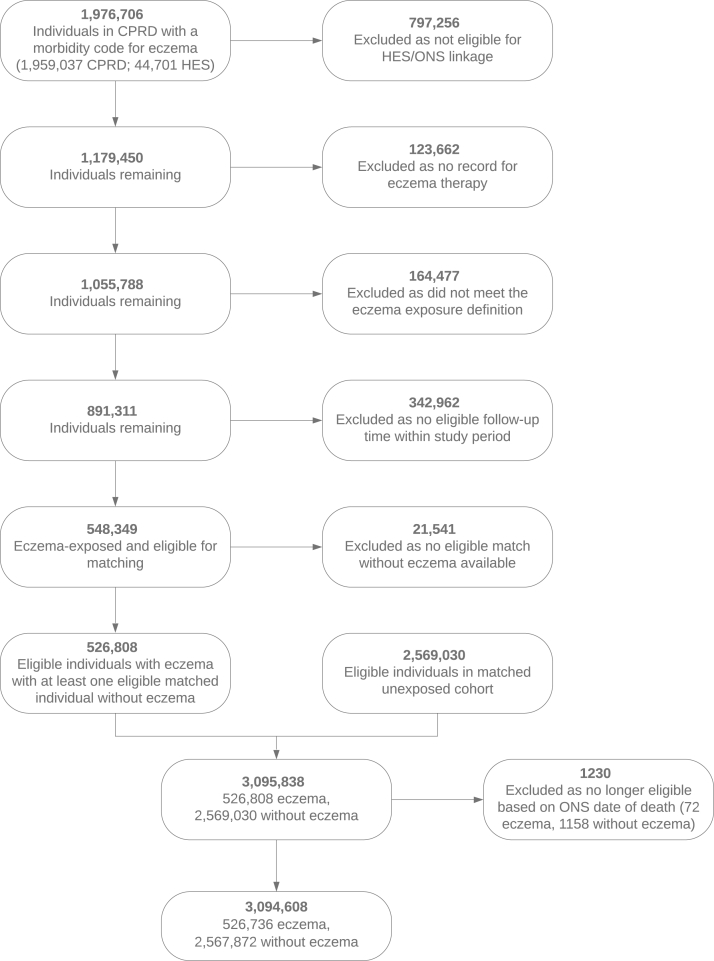
Data flowchart for UK population–based cohort study, 1998–2016.

In total, 3,094,608 individuals (526,736 patients with atopic eczema and 2,567,872 unexposed individuals) were successfully matched and eligible for cohort entry (Fig 1). The median age at cohort entry was 41.8 years, and the median follow-up was 4.5 years (Table II). Among all the patients with atopic eczema, 5.7% of the total follow-up was spent with severe atopic eczema.

**Table II:** Summary statistics for individuals included in the study

Characteristic	Without atopic eczema n = 2,567,872 (83.0%)	With atopic eczema n = 526,736 (17.0%)	Total N = 3,094,608
Follow-up (y), median (first and third quartiles)	4.4 (1.7, 8.9)	5.0 (2.0, 9.6)	4.5 (1.7, 9.0)
At entry into cohort			
Sex, no. (%)			
Male	1,079,198 (42.0)	218,702 (41.5)	1,297,900 (41.9)
Female	1,488,674 (58.0)	308,034 (58.5)	1,796,708 (58.1)
Age (y), median (first and third quartiles), no. (%)	41.7 (27.0, 60.8)	42.3 (25.7, 62.3)	41.8 (26.7, 61.1)
18-19	317,332 (12.4)	81,760 (15.5)	399,092 (12.9)
20-29	471,212 (18.4)	84,511 (16.0)	555,723 (18.0)
30-39	429,028 (16.7)	80,315 (15.2)	509,343 (16.5)
40-49	351,880 (13.7)	69,694 (13.2)	421,574 (13.6)
50-59	328,941 (12.8)	63,942 (12.1)	392,883 (12.7)
60-69	303,674 (11.8)	61,896 (11.8)	365,570 (11.8)
70-79	227,150 (8.8)	52,037 (9.9)	279,187 (9.0)
≥80	138,655 (5.4)	32,581 (6.2)	171,236 (5.5)
IMD, no. (%)			
1 (least deprived)	611,696 (23.8)	126,788 (24.1)	738,484 (23.9)
2	589,085 (22.9)	120,936 (23.0)	710,021 (22.9)
3	508,244 (19.8)	103,632 (19.7)	611,876 (19.8)
4	488,876 (19.0)	100,415 (19.1)	589,291 (19.0)
5 (most deprived)	369,971 (14.4)	74,965 (14.2)	444,936 (14.4)
BMI, no. (%)			
Underweight	65,917 (3.2)	13,442 (3.0)	79,359 (3.1)
Normal weight	947,898 (45.7)	196,737 (44.1)	1,144,635 (45.4)
Overweight	667,042 (32.2)	143,901 (32.2)	810,943 (32.2)
Obese	393,485 (19.0)	92,520 (20.7)	486,005 (19.3)
Smoking, no. (%)			
Nonsmoker	1,293,562 (53.5)	266,116 (51.9)	1,559,678 (53.2)
Current/ex-smoker	1,125,203 (46.5)	246,748 (48.1)	1,371,951 (46.8)
Diabetes, no. (%)	127,484 (5.0)	32,387 (6.1)	159,871 (5.2)
Depression, no. (%)	53,619 (2.1)	15,382 (2.9)	69,001 (2.2)
Anxiety, no. (%)	18,577 (0.7)	5,941 (1.1)	24,518 (0.8)
Asthma, no. (%)	318,263 (12.4)	125,815 (23.9)	444,078 (14.4)
Severe alcohol use, no. (%)	57,215 (2.2)	14,351 (2.7)	71,566 (2.3)
By exit from cohort, no. (%)			
Diabetes	233,623 (9.1)	59,922 (11.4)	293,545 (9.5)
Depression	107,424 (4.2)	31,018 (5.9)	138,442 (4.5)
Anxiety	33,302 (1.3)	10,067 (1.9)	43,369 (1.4)
Asthma	385,700 (15.0)	146,179 (27.8)	531,879 (17.2)
Severe alcohol use	86,306 (3.4)	21,794 (4.1)	108,100 (3.5)

*BMI*, Body mass index; *GUS*, genitourinary system.

The most common causes of death among the atopic eczema–exposed patients were circulatory disease and neoplasms, with infections and diseases of the genitourinary system causing the fewest deaths (see Fig E3 in the Online Repository at www.jacionline.org). The median age at death among the atopic eczema–exposed patients was 83.2 years.

In the primary analysis, there was limited evidence of an association between atopic eczema and all-cause mortality (HR = 1.04; 99% CI = 1.03-1.06 in the adjusted model), but there were somewhat stronger associations with several of the individual causes of death (Table III). Associations were strongest with infections (HR = 1.14; 99% CI = 0.98-1.32), digestive diseases (HR = 1.11; 99% CI = 1.03-1.19), and diseases of the genitourinary system (HR = 1.08; 99% CI = 0.96-1.20).

**Table III:** Association between eczema and all-cause and cause-specific mortality (cause-specific hazards) (N = 3,094,608)

Mortality	n	P-Y at risk	Events	HR and 99% CI∗			
				Unadjusted		Adjusted	
All-cause mortality							
Unexposed	2,567,872	14,925,990	199,645	1.00	(ref)	1.00	(ref)
Exposed	526,736	3,308,778	49,514	1.08	1.06, 1.09	1.04	1.03, 1.06
Cause-specific mortality							
Infections							
Unexposed	2,567,872	14,925,990	2,040	1.00	(ref)	1.00	(ref)
Exposed	526,736	3,308,778	563	1.17	1.01, 1.36	1.14	0.98, 1.32
Neoplasms							
Unexposed	2,567,872	14,925,990	56,873	1.00	(ref)	1.00	(ref)
Exposed	526,736	3,308,778	14,052	1.08	1.05, 1.11	1.06	1.03, 1.09
Circulatory disease							
Unexposed	2,567,872	14,925,990	68,669	1.00	(ref)	1.00	(ref)
Exposed	526,736	3,308,778	16,788	1.06	1.03, 1.09	1.04	1.01, 1.06
Respiratory disease							
Unexposed	2,567,872	14,925,990	28,383	1.00	(ref)	1.00	(ref)
Exposed	526,736	3,308,778	7,670	1.17	1.12, 1.22	1.06	1.02, 1.11
Digestive disease							
Unexposed	2,567,872	14,925,990	9,492	1.00	(ref)	1.00	(ref)
Exposed	526,736	3,308,778	2,451	1.14	1.07, 1.22	1.11	1.03, 1.19
Diseases of the GUS							
Unexposed	2,567,872	14,925,990	3,877	1.00	(ref)	1.00	(ref)
Exposed	526,736	3,308,778	1,008	1.10	0.98, 1.23	1.08	0.96, 1.20
Other causes							
Unexposed	2,567,872	14,925,990	30,311	1.00	(ref)	1.00	(ref)
Exposed	526,736	3,308,778	6,982	1.00	0.96, 1.04	0.99	0.95, 1.03

*GUS*, Genitourinary system; *P-Y*, person years; *ref*, reference.

Unadjusted means no adjustment; adjusted means adjusted for the current calendar period (1998-2001, 2002-2004, 2005-2007, 2008-2010, 2011-2013, and 2014-2016), Index of Multiple Deprivation at cohort entry and time-varying asthma.

∗ Estimated HRs from Cox regression with current age as the underlying time scale, stratified by matched set (matched on age at cohort entry, sex, date at cohort entry, and practice).

The estimated attributable risks confirmed the higher incidence rates of all-cause and cause-specific mortality among those with atopic eczema, although the absolute levels were relatively modest (Table IV^1^^,^^15^). The all-cause mortality-attributable risk was 62 per 100,000 person years (99% CI = 40-84), and the cause-specific mortality-attributable risks were greatest for neoplasms ( 24 per 100,00 person years; 99% CI = 12-35) and circulatory disease (18 per 100,000 person years; 99% CI = 5-31). The greatest population-attributable risks were estimated for infections (1.4%; 99% CI = –0.2-3.1) and digestive disease ( 1.1%; 99% CI = 0.3-1.8).

**Table IV:** Absolute mortality rates, mortality rate differences (attributable risks), and population-attributable risks of all-cause and cause-specific mortality

Mortality	Estimated mortality rate per 100,000 person years in atopic eczema–exposed patients	HR and 99% CI∗		Inverse HR and 99% CI†		Estimated mortality rate and 99% CI per 100,000 person years in people without atopic eczema‡		Estimated mortality rate difference (attributable risk) and 99% CI per 100,000 person years§		Estimated population-attributable risk (%) and 99% CI‖	
All-cause mortality	1496	1.04	1.03-1.06	0.96	0.94-0.97	1435	1413-1457	62	40-84	0.4	0.3-0.6
Cause-specific mortality											
Infections	17	1.14	0.98-1.32	0.88	0.76-1.02	15	13-17	2	0-4	1.4	–0.2-3.1
Neoplasms	425	1.06	1.03-1.09	0.94	0.92-0.97	401	390-412	24	12-35	0.6	0.3-0.9
Circulatory disease	507	1.04	1.01-1.06	0.96	0.94-0.99	490	477-503	18	5-31	0.4	0.1-0.6
Respiratory disease	232	1.06	1.02-1.11	0.94	0.90-0.98	218	209-228	14	4-23	0.6	0.2-1.1
Digestive disease	74	1.11	1.03-1.19	0.90	0.84-0.97	67	62-72	7	2-12	1.1	0.3-1.8
Diseases of the GUS	30	1.08	0.96-1.20	0.93	0.83-1.04	28	25-32	2	–1-5	0.7	–0.4-2.0
Other causes	211	0.99	0.95-1.03	1.01	0.97-1.05	213	204-222	-2	–11-7	–0.1	–0.5-0.3

∗ Comparison of atopic eczema–exposed patients with people without atopic eczema. Estimated HRs from Cox regression with current age as underlying time scale, stratified by matched set (matched on age at cohort entry, sex, date at cohort entry, and practice) comparing atopic eczema–exposed people with atopic eczema–unexposed people. Adjusted for current calendar period (1998-2001, 2002-2004, 2005-2007, 2008-2010, 2011-2013, and 2014-2016), Index of Multiple Deprivation quintile at cohort entry, and time-varying asthma.

† Comparison of people without atopic eczema with atopic eczema–exposed patients.

‡ Calculated by multiplying the estimated incidence rate in atopic eczema–exposed patients by the inverse estimated HR and 99% CI.

§ Calculated by subtracting the estimated incidence rate and 99% CI in people without atopic eczema from the estimated incidence rate in atopic eczema–exposed patients.

‖ Estimated as P(HR – 1)/(1 + P(HR – 1)), where P (the prevalence of atopic eczema) is assumed to be 10%^1^^,^^15^ and HR is the estimated HR comparing atopic eczema–exposed patients with people without atopic eczema.

There was no convincing evidence of effect modification by asthma (see Table E1 in the Online Repository at www.jacionline.org). However, stronger associations were observed in males and younger people (aged 18-39 or 40-59 years, depending on the cause of death) for several causes of death (see Tables E2 and E3 in the Online Repository at www.jacionline.org).

**Fig 2:**
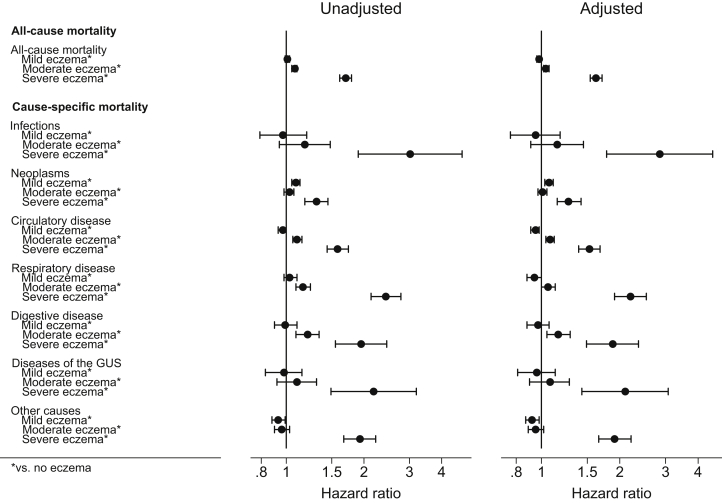
Association between atopic eczema and all-cause and cause-specific mortality, by severity of atopic eczema (n = 3,094,608). Estimated cause-specific HRs from Cox regression with current age as underlying time scale, stratified by matched set (matched on age at cohort entry, sex, date at cohort entry, and practice). Unadjusted means no adjustment; adjusted means adjusted for the current calendar period (1998-2001, 2002-2004, 2005-2007, 2008-2010, 2011-2013, and 2014-2016), Index of Multiple Deprivation at cohort entry, and time-varying asthma. *GUS*, Genitourinary system.

**Fig 3:**
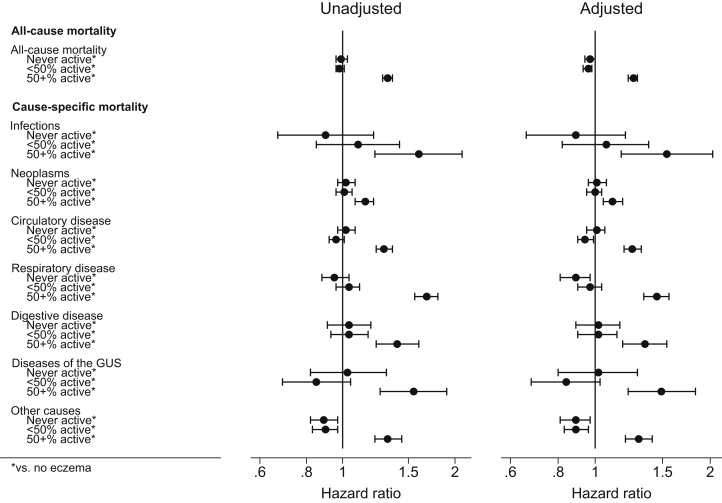
Association between atopic eczema and all-cause and cause-specific mortality, by activity of atopic eczema (assessed during the first 12 months of follow-up, assuming active eczema for 3 months after a single health care contact), excluding the first 12 months of follow-up (n = 2,614,344). Estimated cause-specific HRs from Cox regression with current age as underlying time scale, stratified by matched set (matched on age at cohort entry, sex, date at cohort entry, and practice). Unadjusted means no adjustment; adjusted means adjusted for the current calendar period (1998-2001, 2002-2004, 2005-2007, 2008-2010, 2011-2013, and 2014-2016), Index of Multiple Deprivation at cohort entry, and time-varying asthma. *GUS*, Genitourinary system.

A total of 34,610 people, contributing 187,910 years of follow-up time, were classified as having severe atopic eczema, of whom 4,446 died. Associations were substantially stronger in patients with severe atopic eczema than in those without eczema, in particular, for deaths due to infections (HR = 2.85; 99% CI = 1.78-4.55 in the adjusted model), respiratory disease (HR = 2.20; 99% CI = 1.91-2.53), diseases of the genitourinary system (HR = 2.10; 99% CI = 1.43-3.07), and other causes (HR = 1.91; 99% CI = 1.66-2.21), as well as for mortality overall (HR = 1.62; 99% CI = 1.54-1.71) (Fig 2 and see Table E4 in the Online Repository at www.jacionline.org). Associations were also stronger in people with the most active atopic eczema (Fig 3 and see Table E5 in the Online Repository at www.jacionline.org).

### Sensitivity analyses and model checking

The sensitivity analysis results are presented in Table I.

Schöenfeld residual plots did not provide compelling evidence that the proportional hazards assumption was not satisfied in the primary analysis models.

## Discussion

### Principal findings

This study found limited evidence of increased all-cause mortality in those with atopic eczema, although there was a small increase in the rate of death due to some specific causes. The differences on the absolute scale were modest owing to the low overall mortality rates. There was, however, strong evidence of elevated mortality among patients with the most severe or active eczema.

### Comparison with other studies

Two previous studies, both of which used Danish hospital registry data, assessed whether atopic eczema is associated with increased mortality.^8^^,^^9^ Egeberg et al assessed mortality during the 10 years following hospital admission for atopic eczema; they reported a 71% increased hazard for mortality compared with that in the Danish general population.^8^ These findings are consistent with our observation of a 62% increased hazard of death in those with severe eczema in the UK population. Thyssen et al reported a 27% greater hazard of mortality in people diagnosed with atopic eczema in outpatient hospital clinics or certain private dermatology clinics (moderate) or during hospital admission (severe) than in people without atopic eczema in the Danish general population.^9^ As the majority of patients are likely to represent moderate eczema, the findings of Thyssen et al are consistent with the small increase in rates of death that we observed for those with moderate atopic eczema.

Thyssen et al^9^ examined cause-specific mortality, reporting increased rates of death due to cardiovascular, infectious, and urogenital disease, but with very wide CIs, as the sample size was small (N = 8,686, with fewer than 10 deaths for most specific causes). Our study observed similarly increased rates of death due to these causes, and it additionally found increased rates of death due to respiratory disease and digestive diseases, particularly in those with the most severe or active atopic eczema.

The finding of increased hazard of death associated with infectious diseases is supported by previous observations from our group, and from others, of increased risks of cutaneous and extracutaneous infections in those with atopic eczema.^16^^,^^17^ Possible mechanisms of this increased risk include skin barrier and immunologic dysfunction. Use of systemic immunosuppressants is an alternative explanation; however, adjusting for this covariate in sensitivity analyses did not attenuate the association.

The increased hazard for death due to circulatory disease in those with the most severe or active atopic eczema is consistent with reports from our group and others.^6^ Of note, the findings regarding circulatory disease mortality in this article do not exactly duplicate those for cardiovascular death in our previous work owing to differing exclusion criteria and outcome definitions (leading to mortality rates in eczema-unexposed individuals of 319 and 460 per 100,000 person years, respectively).^6^ Mechanisms for the increased risk may include reduced physical actvity^18^ and reduced sleep quality.^19^

Our observation of a 2-fold increase in hazard of mortality among those with severe atopic eczema related to urogenital disease highlights an area that requires more research. Previous limited reports have suggested that children with atopic eczema may have an increased risk of nephrotic syndrome.^20^, ^21^, ^22^, ^23^ Use of cyclosporine for severe atopic eczema could lead to an increased risk of chronic kidney disease, although similar estimates were seen in our study when adjusting, albeit fairly crudely, for use of systemic treatment for atopic eczema. Increased risks of chronic kidney disease have also been observed in other inflammatory dermatoses such as psoriasis.^24^, ^25^, ^26^ Understanding of these associations is currently limited; the proposed mechanisms include increased prevalence of metabolic syndrome and cardiovascular disease, use of nephrotoxic drugs, or relationship to chronic inflammation over many years.^27^

The increased mortality due to respiratory disease may relate to residual confounding on account of misclassification of asthma. Alternative explanations could be adverse effects of drugs (eg, azathioprine) or residual confounding from smoking.

Our observation of increased mortality due to digestive diseases is novel and warrants replication and further analyses. Atopic eczema is known to be associated with eosinophilic esophagitis, but there are limited studies addressing potential gastrointestinal diseases that might increase mortality risk, such as peptic ulceration. Alternative explanations may include adverse effects of drugs or residual confounding from alcohol use.

The observation that associations between atopic eczema and mortality (on a relative scale) were highest in younger individuals for most causes of death may be explained by younger people being relatively less likely to die of most causes regardless of their atopic eczema status, meaning that there was greater opportunity for a raised HR, which is consistent with the findings of other studies of inflammatory skin diseases.^28^

We chose not to look specifically at death due to suicide over concerns regarding the reliability of coding for this cause of death. However, patients with atopic eczema have been found to be at increased risk of suicidal ideation and suicide attempts,^29^ so suicide may be contributing to the observed increased hazard of death due to “other causes” among those with the most severe or active atopic eczema.

### Strengths and limitations

This study is, to our knowledge, the largest study to date to assess the association between atopic eczema and all-cause and cause-specific mortality. We used population-based data from UK general practice with linked data on hospitalization and cause-specific mortality.^30^^,^^31^ Previous studies have demonstrated that this population is largely representative of the general UK population.^11^ We used a validated algorithm to identify atopic eczema, and our approach to defining atopic eczema severity demonstrated a distribution of severity similar to that in the published literature.^12^^,^^32^ We used a directed acyclic graph^13^ to inform the identification of covariates and avoid collider bias. The primary analysis included all successfully matched individuals.

Limitations of the study include the possibility for confounding, information bias, and selection bias because of missing data. It is not possible to disentangle the effects of therapy and severity, as those with more severe disease are given specific therapies. It is also not possible to truly disentangle the effects of atopic and nonatopic eczema, as these data do not contain total or specific IgE levels. ONS mortality data contain the underlying cause of death as certified by a doctor or a coroner. Although extensive validation checks are built into the process,^33^ imprecisions may remain. However, we would not expect any misclassification of cause of death to be differential with respect to atopic eczema diagnosis. Although the algorithm used to identify patients with atopic eczema was demonstrated to have a positive predictive value of 82% (95% CI = 73%-89%) in a previous validation study,^12^ there remains the possibility of misclassification of individuals with atopic eczema, which may have led to bias.

People were matched within 15-year age intervals, as finer age matching led to a significant loss of those with atopic eczema with no eligible matches. However, age was accounted for as the underlying time scale in all analyses, closely adjusting for the confounding effects of age. Although sparsity of ethnicity data in the early years of the study period meant that ethnicity could not be included in the main analyses, the results adjusted for ethnicity in the subsample with complete data were consistent with those from the primary analysis conducted on the same reduced cohort, suggesting limited bias from omission of this covariate.

Individuals with worse health are more likely to see their GP, and given that our algorithm used to identify patients with atopic eczema required a GP visit whereas visiting a GP was not required in our comparison cohort, there is the potential for overestimation of the relative mortality risk among patients with atopic eczema. Although there was some evidence of this in the sensitivity analysis restricting the primary analysis to individuals with at least 1 consultation with their GP in the year before cohort entry, in the equivalent atopic eczema severity and activity analyses there remained strong evidence of increased mortality in those with the most severe or active eczema, suggesting that such bias was not the cause of the observed findings.

Assessing major long-term health outcomes in a disorder such as atopic eczema that frequently starts in early childhood may be challenging owing to possible survival bias.^34^^,^^35^ As survival bias would bias the association toward the null, it cannot explain our findings, and it could potentially have led to underestimation of the associations.^36^ Onset confounding (differential time since atopic eczema onset) may bias the results, although the direction of bias will depend on whether the rate of mortality increases or decreases with time since onset. The attenuation of results for most causes of death when restricting to incident atopic eczema diagnoses suggests that such bias may be present to some degree, although interpretation is difficult given the possibility of misclassification of preexisting (childhood) eczema as incident (adulthood) eczema.

Misclassification of disease severity is possible (for example, when examining whether patients with severe disease used only topical treatment or patients requiring systemic therapies subsequently went into “remission” or had predominantly mild disease). Although the atopic eczema severity and activity aspects of the algorithm are unvalidated and based on clinical judgment, the proportions of individuals in each group are consistent with those in previous reports.^37^

Although we used 99% CIs and an implied 1% level of statistical significance to reduce the risk of type 1 error in the primary analyses, the total number of analyses undertaken (ie, those including sensitivity analyses) means that type 1 errors remain a possibility.

### Conclusions

We have shown a small increase in the rate of mortality due to several causes in individuals with atopic eczema. This increased risk is particularly elevated in individuals with severe or more active disease. People with severe atopic eczema warrant thorough health assessments, including preventative approaches. Useful next steps would be to improve our understanding of the likely causes of these increased hazards for mortality in severe or predominantly active atopic eczema and whether proactive reduction in inflammation with systemic treatments can decrease overall and cause-specific mortality.

**Key messages**

• Our study found a small increased hazard for mortality due to several causes in those with atopic eczema.
• Patients with severe and predominantly active atopic eczema were found to have an increased hazard for all-cause mortality compared with those without eczema.
• The excess risk of death in patients with severe atopic eczema was highest for deaths due to infections, respiratory disease, and diseases of the genitourinary system.
