# Supplementary material for: Atopic eczema in adulthood and mortality: UK population–based cohort study, 1998-2016
Source: J Allergy Clin Immunol. 2021 May;147(5):1753–63. doi: 10.1016/j.jaci.2020.12.001 (PMC8098860; doi:10.1016/j.jaci.2020.12.001)
Supplement: Supplementary Material [file mmc1.docx]

**ONLINE REPOSITORY**

Contents

[**Methods E1**. Additional methods. 4](#_Toc55504716)

[**Methods E2**. Pre-specified active atopic eczema definition. 8](#_Toc55504717)

[**Methods E3**. Exploratory mediation analysis. 9](#_Toc55504718)

[**Table E1**. Association between atopic eczema and all-cause and cause-specific mortality (cause-specific hazards), by exposure to time-varying asthma. n = 3,094,608. Adjusted models^1^. 12](#_Toc55504719)

[**Table E2**. Association between atopic eczema and all-cause and cause-specific mortality (cause-specific hazards), by sex. n = 3,094,608. Adjusted models^1^. 14](#_Toc55504720)

[**Table E3**. Association between atopic eczema and all-cause and cause-specific mortality (cause-specific hazards), by current age. n = 3,094,608. Adjusted models^1^. 16](#_Toc55504721)

[**Table E4**. Association between atopic eczema severity and all-cause and cause-specific mortality (cause-specific hazards). n = 3,094,608. 18](#_Toc55504722)

[**Table E5**. Association between atopic eczema activity and all-cause and cause-specific mortality (cause-specific hazards), by activity of atopic eczema (assessed during the first twelve months of follow-up, assuming active eczema for three months after a single health care contact), excluding the first 12 months of follow-up. n = 2,614,344. 20](#_Toc55504723)

[**Table E6**. Association between atopic eczema and all-cause and cause-specific mortality (cause-specific hazards), with exposed patients restricted to those with incident atopic eczema. Fitted to individuals from valid matched sets^1^. n = 1,552,870. 22](#_Toc55504724)

[**Table E7**. Association between atopic eczema and all-cause and cause-specific mortality (cause-specific hazards) restricted to people with at least one consultation with their GP in the year prior to cohort entry. Fitted to individuals from valid matched sets^1^. n = 2,478,735. 23](#_Toc55504725)

[**Table E8**. Association between atopic eczema and all-cause and cause-specific mortality (cause-specific hazards), using first redefined cohort^1^. n = 3,132,843. 24](#_Toc55504726)

[**Table E9**. Association between atopic eczema and all-cause and cause-specific mortality (cause-specific hazards), using second redefined cohort^1^. n = 3,782,266. 25](#_Toc55504727)

[**Table E10**. Association between atopic eczema and cardiovascular outcomes, using only people registered from 2006 onwards with complete ethnicity data. Fitted to individuals from valid matched sets^1^. n = 339,734. 26](#_Toc55504728)

[**Table E11**. Association between atopic eczema and all-cause and cause-specific mortality (cause-specific hazards), treating time since diagnosis as a time-varying exposure. n = 3,094,608. 27](#_Toc55504729)

[**Table E12**. Association between atopic eczema severity and all-cause and cause-specific mortality (cause-specific hazards) restricted to people with at least one consultation with their GP in the year prior to cohort entry. n = 2,478,735. 29](#_Toc55504730)

[**Table E13**. Association between atopic eczema activity and all-cause and cause-specific mortality (cause-specific hazards) restricted to people with at least one consultation with their GP in the year prior to cohort entry, by activity of atopic eczema (assessed during the first twelve months of follow-up, assuming active eczema for three months after a single health care contact), excluding the first 12 months of follow-up. n = 2,108,343. 31](#_Toc55504731)

[**Table E14**. Association between atopic eczema activity and all-cause and cause-specific mortality (cause-specific hazards), by activity of atopic eczema (second activity definition: assessed during the first twelve months of follow-up, assuming active eczema for three months after a single health care contact), without excluding the first 12 months of follow-up. n = 3,094,608. 33](#_Toc55504732)

[**Table E15**. Association between atopic eczema activity and all-cause and cause-specific mortality (cause-specific hazards), by activity of atopic eczema (pre-specified activity definition: assessed across all of follow-up, assuming active eczema for 12 months after two health care contacts). n = 3,094,608. 35](#_Toc55504733)

[**Table E16**. Association between atopic eczema activity and all-cause and cause-specific mortality (cause-specific hazards), by activity of atopic eczema (pre-specified activity definition: assessed across all of follow-up, assuming active eczema for 12 months after two health care contacts), restricted to patients with at least 5 years of follow-up. Fitted to individuals from valid matched sets^1^. n = 1,159,330. 37](#_Toc55504734)

[**Table E17**. Covariate summary statistics. Number (%) unless otherwise stated. 39](#_Toc55504735)

[**Table E18**. Association between eczema and all-cause and cause-specific mortality (cause-specific hazards). Fitted to individuals with complete data for all variables included in the models and from valid matched sets^1^. n = 2,278,944. 41](#_Toc55504736)

[**Table E19**. Association between atopic eczema severity and all-cause and cause-specific mortality (cause-specific hazards). Fitted to individuals with complete data for all variables included in the models and from valid matched sets^1^. n = 2,278,944. 42](#_Toc55504737)

[**Table E20**. Association between atopic eczema activity and all-cause and cause-specific mortality (cause-specific hazards), by activity of atopic eczema (assessed during the first twelve months of follow-up, assuming active eczema for three months after a single health care contact), excluding the first 12 months of follow-up. Fitted to individuals with complete data for all variables included in the models and from valid matched sets^1^. n = 1,882,012. 44](#_Toc55504738)

[**Table E21**. Summary statistics comparing people with registrations from 2006 onwards with the overall cohort. 46](#_Toc55504739)

[**Table E22**. Association between atopic eczema and all-cause and cause-specific mortality (cause-specific hazards) restricted to people registered from 2006 onwards. Fitted to individuals with complete data for all variables included in the models and from valid matched sets^1^. n = 301,693. 48](#_Toc55504740)

[**Table E23**. Association between atopic eczema and all-cause and cause-specific mortality (cause-specific hazards), additionally adjusted for ever systemic drug or high-dose oral glucocorticoid use. Fitted to individuals with complete data for all variables included in the models and from valid matched sets^1^. n = 2,278,944. 49](#_Toc55504741)

[**Table E24**. Association between atopic eczema and all-cause and cause-specific mortality (cause-specific hazards), additionally adjusted for current high-dose oral glucocorticoid use. Fitted to individuals with complete data for all variables included in the models and from valid matched sets^1^. n = 2,278,944. 51](#_Toc55504742)

[**Fig. E1**. Graphical depiction of study design. 53](#_Toc55504743)

[**Fig. E2**. Directed acyclic graph used to inform the identification of covariates and mediators. BMI: Body mass index; SES: Socioeconomic status. Arrows between covariates have been omitted to ensure that the figure can be clearly read. 54](#_Toc55504744)

[**Fig. E3**. Stacked cause-specific cumulative incidence functions up to age 100 years among people with atopic eczema. Estimated non-parametrically in the full sample, allowing for competing risks. GUS: Genitourinary system. 55](#_Toc55504745)

# **Methods E1**. Additional methods.

**Study population**

*Defining atopic eczema patients*

The exposed cohort included all patients with atopic eczema, with onset defined at the latest of an atopic eczema diagnosis and two atopic eczema treatments (on separate dates), consistent with a validation study demonstrating a positive predictive value in adults of 82% (95% CI 73%-89%).^1^ Atopic eczema diagnostic codes were identified in CPRD (using Read codes) and HES (using ICD-10 codes in the primary diagnosis field of any episode). Treatments included atopic eczema-related prescriptions from primary care, including emollients, topical and oral corticosteroids, topical calcineurin inhibitors (tacrolimus and pimecrolimus) and systemic immunosuppressants (methotrexate, ciclosporin, mycophenolate mofetil, azathioprine), as well as phototherapy records from CPRD or Office of Population Censuses and Surveys (OPCS) Classification of Interventions and Procedures codes in HES.

*Defining matched unexposed individuals*

For each patient with atopic eczema, we randomly matched without replacement up to five individuals without eczema by age (within 15 years), sex, and general practice. We matched on the point of entry to study follow-up in calendar date order (i.e. individuals in the matched cohort were assigned first to those with earliest study entry) to avoid time-related bias. We allowed a 15-year age difference for matching to increase the likelihood of successfully matching all patients with atopic eczema and therefore maximise the representativeness of our cohort (though analyses were conducted on the current age timescale, allowing closer control for age). Unexposed individuals were required to have at least one year of follow-up in CPRD and no history of atopic eczema when matched. Any patients with an atopic eczema diagnosis were included in the pool of eligible unexposed patients up until the date of their atopic eczema diagnosis. Prior to their atopic eczema diagnosis, these patients were not considered to have atopic eczema (as there was no evidence of this from their CPRD records, going back as far as such records were available, including prior to the study period) and were therefore eligible to contribute to unexposed person time. Patients with an atopic eczema diagnosis who did not go on to meet the full definition of atopic eczema (at least one diagnosis code and two treatment codes on separate dates) were also in the pool of eligible unexposed patients up until the date of their atopic eczema diagnosis code. Removing these patients from the pool of eligible unexposed at the point of diagnosis rather than allowing them to remain until they met the full validated definition of atopic eczema ensured greater certainty that the pool of unexposed patients did not have atopic eczema.

**Covariates**

*Calendar period* was considered in the following categories: 1998-2001, 2002-2004, 2005-2007, 2008-2010, 2011-2013, 2014-2016.

We used *index of multiple deprivation* (IMD) as a proxy for socioeconomic deprivation. IMD was measured using quintiles of patient-level IMD scores linked via the practice and patient postcode (where patient-level data was available for patients eligible for linkage to IMD data). We used the 2007 version of IMD data (IMD data is available for the years: 2004, 2007, 2010 and 2015); we chose this as the midpoint of the study (January 1998 to March 2016). However, where patient-level data were unavailable, we used practice-level data from England (due to the requirement for HES linkage, we used English data only) in 2010 (the closest available English practice-level data to 2007, i.e. the version of patient-level data used).

Patients were defined as having *diabetes mellitus* (type I, type II, or not specified), *asthma*, *depression* (classic unipolar depression diagnoses of various severities) and *anxiety* (generalised anxiety disorder and panic disorder) on the date of their first relevant morbidity code in primary or secondary care, which may have been after cohort entry. *Harmful alcohol use* was defined based on morbidity coding in primary care suggesting harmful or heavy alcohol use (including alcohol dependency codes and codes related to physical/psychological harm related to alcohol use) or a prescription for drugs used to maintain abstinence (acamprosate, disulfiram, or nalmefene). Individuals were defined as harmful alcohol users on the date of the first record of a relevant morbidity code or prescription, which may again have been after cohort entry. These variables were considered as time-updated covariates, with each disease/condition considered to be present from the point of first recording to the end of the study period.

*Smoking* and *body mass index* (BMI) were defined based on primary care records for these measures recorded closest to cohort entry date. The identification of lifestyle variables was pragmatically based on the status recorded closest to the cohort entry date, with records within -1 year to +1 month from cohort entry date regarded as the best, +1 months to +1 years from cohort entry date being second best, the nearest before -1 year from cohort entry date as the third best, and within +1 year from cohort entry date being the worst. Smoking status was classified as: current/ex-smoker or non-smoker. Read codes for BMI category were not used (because they are rarely recorded); instead BMI was calculated using the height and weight measures recorded closest to cohort entry date. BMI was classified using World Health Organisation categories: (underweight (<18.5 kg/m2), normal weight (18.5–24.9 kg/m2), overweight (25–29.9 kg/m2), obese (≥30 kg/m2)).

*Ethnicity* was assigned to five categories: White, South Asian, Black, Other or Mixed. First the most common ethnicity in CPRD was used, then the latest ethnicity in CPRD was used where several ethnicities were recorded equally, and finally HES ethnicity was used where CPRD ethnicity was missing. Since the quality of the recording for ethnicity was only acceptable from 2006 onwards, we restricted our study population to those remaining registered with the CPRD general practice after 1 January 2006^2^.

Patients were defined as being exposed to *systemic drugs* (azathioprine, ciclosporin, mycophenolate and methotrexate) at their first recorded prescription identified from primary care records. We identified prescriptions for oral corticosteroids with glucocorticoid activity (prednisolone, betamethasone, deflazacort, dexamethasone, hydrocortisone, methylprednisolone, prednisone, triamcinolone, and cortisone) and converted the daily dose prescribed to prednisolone equivalent dose. *High-dose oral glucocorticoid* use was defined as a dose of 20 mg/day or more prednisolone equivalent dose (PED). Exposure to high-dose oral glucocorticoids was defined using two approaches: i) “ever” use (exposure from the first recorded prescription), and ii) “current” use (exposed periods covering each course of treatment plus a subsequent 90 day period only).

**Defining severe and active eczema**

Atopic eczema severity was defined as a time-updated variable for atopic eczema patients. Atopic eczema patients were considered to have mild disease by default. They were classified as having “moderate” atopic eczema at the first of: i) a second potent topical corticosteroid treatment within one year or ii) a first topical calcineurin inhibitor treatment.^3^ Atopic eczema patients were classified as having “severe” atopic eczema at the first of: i) a systemic immunosuppressant treatment, ii) a phototherapy code in CPRD or HES, or iii) a referral for atopic eczema. Once defined as moderate, atopic eczema patients remained as such unless they developed severe atopic eczema; once defined as severe, atopic eczema patients remained as such, similar to established approaches for defining severity in psoriasis studies.^4^ At any given point during follow-up, atopic eczema patients therefore belonged to one of three severity categories: mild, moderate, or severe.

Atopic eczema activity was characterised using the first 12 months of follow-up in a post-hoc analysis as results from our pre-specified analysis were suggestive of time-related bias (see Methods E2). “Active” disease started at any single CPRD or HES eczema record (either diagnosis or treatment) and was assumed to last for 3 months (considered a reasonable interval between prescriptions), unless another eczema record appeared. Atopic eczema patients were then split into three categories for analysis: those who never had active atopic eczema during the first year of follow-up, those who had active atopic eczema for less than 50% of the first year of follow-up, and those who had active atopic eczema for at least 50% of the first year of follow-up. The first 12 months of follow-up were subsequently excluded from the analysis to ensure that activity was defined using a non-overlapping period.

1. Abuabara K, Magyari AM, Hoffstad O, Jabbar-Lopez ZK, Smeeth L, Williams HC, et al. Development and Validation of an Algorithm to Accurately Identify Atopic Eczema Patients in Primary Care Electronic Health Records from the UK. J Invest Dermatol 2017; 137:1655-62.

2. Mathur R, Bhaskaran K, Chaturvedi N, Leon DA, vanStaa T, Grundy E, et al. Completeness and usability of ethnicity data in UK-based primary care and hospital databases. J Public Health (Oxf) 2014; 36:684-92.

3. National Institute for Health and Care Excellence. Tacrolimus and pimecrolimus for atopic eczema. Technology appraisal guidance: NICE; 2004.

4. Gelfand J, Troxel A, Lewis J, Kurd S, Shin D, Wang X, et al. The risk of mortality in patients with psoriasis: results from a population-based study. Arch Dermatol 2007; 143:1493-9.

# **Methods E2**. Pre-specified active atopic eczema definition.

In the main paper we report that, “Atopic eczema activity was characterised using the first 12 months of follow-up in a post-hoc analysis as results from our pre-specified analysis were suggestive of time-related bias.” This section details our pre-specified active atopic eczema definition and briefly reports the results from the analyses and sensitivity analyses we conducted using this definition.

In the pre-specified active atopic eczema definition, “active” disease started at the latest of two CPRD or HES atopic eczema records (either diagnoses or treatment) appearing within any one year period. Active disease was assumed to last for 12 months, unless another atopic eczema record appeared, in which case its duration was prolonged for another 12 months. Atopic eczema patients were subsequently split into three categories for analysis: those who never had active atopic eczema during follow-up, those who had active atopic eczema for less than 50% of follow-up, and those who had active atopic eczema for at least 50% of follow-up.

As with the definition of atopic eczema activity used in the main paper, the primary analysis was repeated using the pre-specified definition of atopic eczema activity.

As a sensitivity analysis, the analysis using the pre-specified definition of atopic eczema activity was repeated, restricted to patients with at least 5 years of follow-up, in order to explore any potential bias caused by atopic eczema patients with short follow-up periods being more likely to have either none or all of their follow-up with active atopic eczema

Using the pre-specified definition of active atopic eczema, effect estimates were stronger in patients with the most active atopic eczema, though a protective effect of moderately active atopic eczema was also observed (Table E15).

In the sensitivity analysis, restricting the analysis to patients with at least 5 years of follow-up (42.3% of patients) diminished or completely removed the apparent protective effect of moderately active atopic eczema (Table E16).

We therefore concluded that results from our pre-specified analysis were suggestive of time-related bias.

# **Methods E3**. Exploratory mediation analysis.

*Methods*

We used a directed acyclic graph to inform the identification of potential mediators and to avoid collider bias (Fig. E2). We considered variables relating to diabetes mellitus, asthma, depression, anxiety, harmful alcohol use, smoking status and body mass index (BMI) to be potential mediators. Full details regarding the definition of these variables can be found in Methods E1.

We conducted an exploratory analysis where we additionally adjusted for potential mediators (i.e. variables on the causal pathway between atopic eczema and mortality) (smoking and BMI, and time-varying depression, anxiety, diabetes and harmful alcohol use; the “mediation” model). Individuals with missing BMI or smoking data were excluded (and matching preserved). The adjusted model was first refitted on the mediation analysis sample for comparison.

Two additional sensitivity analyses were conducted: i) The exploratory mediation analysis was repeated on a subset of patients registered from 2006 onwards, to reduce potential selection bias due to missing data, as data on covariates subject to missingness (BMI and smoking) would be expected to be more complete from 2006 onwards; ii) The exploratory mediation analysis was repeated with additional adjustment for systemic drug and high-dose oral glucocorticoid use, to examine how much of any observed association may be due to systemic drug and high-dose oral glucocorticoid use.

*Results*

Of the 3,094,608 individuals in the study, 585,859 (18.9%) were excluded from the exploratory mediation analysis as they did not have complete data on the analysis variables (18.5% missing BMI and 5.3% missing smoking), and a further 229,805 (7.4%) individuals were excluded as they had no remaining matches, leaving a final analysis sample of 2,278,944 individuals (440,317 atopic eczema patients and 1,838,627 unexposed individuals). Distributions of variables did not differ substantially between those included in the mediation analysis sample and the overall sample, except for a somewhat higher proportion of atopic eczema patients (19.3% vs. 17.0%), more females (62.9% vs. 58.1%), and fewer of the very youngest (18–19 years) patients (5.7% vs. 12.9%) in the mediation analysis sample (Table E17). In the primary analysis model (binary atopic eczema) there was consistent partial attenuation of the estimated associations across all causes of death in the exploratory mediation analysis, weakening evidence for some of the associations once mediators were accounted for (Table E18). In analyses considering atopic eczema severity and activity there was similar attenuation in the mediation models, though there remained evidence of substantially stronger associations in patients with the most severe and active atopic eczema (Tables E19-E20).

In the additional sensitivity analyses: i) Only 301,693 individuals had registrations from 2006 onwards, complete data for mediation analysis variables and remained in valid matched sets. This small subset of patients differed substantially from the main mediation analysis sample, particularly by being younger (Table E21), and the HRs differed markedly, with wide CIs (Table E22); ii) Associations adjusted for ever systemic drug (Table E23) or current high dose oral glucocorticoid use (Table E24) showed only limited attenuation relative to the main mediation analysis. Associations adjusted for ever high dose oral glucocorticoid use showed somewhat more attenuation (Table E17).

*A note on missing data handling*

The primary analysis and most secondary analyses included all successfully matched individuals. However, individuals with missing BMI or smoking data were excluded from the exploratory mediation analysis sample (and matching preserved). Of those initially identified eligible atopic eczema patients and matched unexposed individuals, 18.9% were excluded from subsequent analyses as they did not have complete data on the analysis variables (18.5% missing BMI and 5.3% missing smoking), and a further 7.4% individuals were excluded as they had no remaining matches. Distributions of variables did not differ substantially between those included in the final analysis sample and the overall sample, except for a somewhat higher proportion of atopic eczema patients (19.3% vs. 17.0%), more females (62.9% vs. 58.1%), and fewer of the very youngest (18–19 years) patients (5.7% vs. 12.9%) in the analysis sample (Table E17). This suggests that our mediation analysis sample was largely representative of the overall sample.

The adjusted model refitted on the mediation analysis sample (Tables E18-E20 for the primary, severity and activity analyses, respectively) can be compared with the adjusted model fitted on the full sample (i.e. including individuals with missing BMI or smoking data; Tables 3, E4 and E5 for the primary, severity and activity analyses, respectively) to allow assessment of any bias introduced in the mediation analysis by excluding these individuals. These models gave very similar results, particularly for the severity and activity analyses, suggesting that excluding these individuals induced little bias.

We attempted to explore the issue of missing data further via a sensitivity analysis in which the exploratory mediation analysis was repeated on a subset of patients registered from 2006 onwards (in which we would expect data on covariates subject to missingness - BMI and smoking - to be more complete). However, only 301,693 individuals had registrations from 2006 onwards, complete data for mediation analysis variables and remained in valid matched sets, and this small subset of patients differed substantially from the primary analysis sample, particularly by being younger (Table E21), and the HRs differed markedly, with wide confidence intervals (Table E22). This sensitivity analysis was therefore not deemed informative with respect to the issue of missing data.

We believe the missing BMI and smoking data are likely to be missing not at random (as missingness is likely to depend on the actual values). Complete case analysis is valid where the missingness is independent of each outcome (death or specific cause of death), conditional on the model covariates.^1^ We believe that there are no factors leading to missingness that would independently affect mortality, therefore using a complete-case analysis was valid.

This belief, taken in combination with the empirical results summarised above, gives us some confidence that the mediation analysis findings are not subject to substantial bias arising from the exclusion of individuals with missing data.

1. White IR, Carlin JB. Bias and efficiency of multiple imputation compared with complete-case analysis for missing covariate values. S*tat Med* 2010; 29(28): 2920-31.

# **Table E1**. Association between atopic eczema and all-cause and cause-specific mortality (cause-specific hazards), by exposure to time-varying asthma. n = 3,094,608. Adjusted models^1^.

|  |  |  |  |  | | Interaction |
| --- | --- | --- | --- | --- | --- | --- |
|  | n | P-Y at risk | Events | HR & 99% CI^2^ | | p-value |
| **All-cause mortality** |  |  |  |  |  |  |
|  |  |  |  |  |  |  |
| All-cause mortality |  |  |  |  |  | 0.001 |
| No asthma |  |  |  |  |  |  |
| Unexposed | 2,249,556 | 12,913,510 | 166,817 | 1.00 | (ref) |  |
| Exposed | 400,569 | 2,466,989 | 38,050 | 1.05 | 1.03, 1.07 |  |
| Asthma |  |  |  |  |  |  |
| Unexposed | 385,645 | 2,012,479 | 32,828 | 1.00 | (ref) |  |
| Exposed | 146,166 | 841,789 | 11,464 | 1.00 | 0.94, 1.06 |  |
|  |  |  |  |  |  |  |
| **Cause-specific mortality** |  |  |  |  |  |  |
|  |  |  |  |  |  |  |
| Infections |  |  |  |  |  | 0.44 |
| No asthma |  |  |  |  |  |  |
| Unexposed | 2,249,556 | 12,913,510 | 1,672 | 1.00 | (ref) |  |
| Exposed | 400,569 | 2,466,989 | 438 | 1.19 | 1.00, 1.41 |  |
| Asthma |  |  |  |  |  |  |
| Unexposed | 385,645 | 2,012,479 | 368 | 1.00 | (ref) |  |
| Exposed | 146,166 | 841,789 | 125 | 1.12 | 0.61, 2.08 |  |
|  |  |  |  |  |  |  |
| Neoplasms |  |  |  |  |  | 0.003 |
| No asthma |  |  |  |  |  |  |
| Unexposed | 2,249,556 | 12,913,510 | 48,426 | 1.00 | (ref) |  |
| Exposed | 400,569 | 2,466,989 | 11,076 | 1.08 | 1.04, 1.11 |  |
| Asthma |  |  |  |  |  |  |
| Unexposed | 385,645 | 2,012,479 | 8,447 | 1.00 | (ref) |  |
| Exposed | 146,166 | 841,789 | 2,976 | 1.02 | 0.91, 1.15 |  |
|  |  |  |  |  |  |  |
| Circulatory disease |  |  |  |  |  | 0.25 |
| No asthma |  |  |  |  |  |  |
| Unexposed | 2,249,556 | 12,913,510 | 58,762 | 1.00 | (ref) |  |
| Exposed | 400,569 | 2,466,989 | 13,326 | 1.04 | 1.01, 1.07 |  |
| Asthma |  |  |  |  |  |  |
| Unexposed | 385,645 | 2,012,479 | 9,907 | 1.00 | (ref) |  |
| Exposed | 146,166 | 841,789 | 3,462 | 0.98 | 0.87, 1.10 |  |
|  |  |  |  |  |  |  |
| Respiratory disease |  |  |  |  |  | 0.02 |
| No asthma |  |  |  |  |  |  |
| Unexposed | 2,249,556 | 12,913,510 | 19,978 | 1.00 | (ref) |  |
| Exposed | 400,569 | 2,466,989 | 4,822 | 1.09 | 1.03, 1.15 |  |
| Asthma |  |  |  |  |  |  |
| Unexposed | 385,645 | 2,012,479 | 8,405 | 1.00 | (ref) |  |
| Exposed | 146,166 | 841,789 | 2,848 | 1.02 | 0.90, 1.15 |  |
|  |  |  |  |  |  |  |
| Digestive disease |  |  |  |  |  | 0.70 |
| No asthma |  |  |  |  |  |  |
| Unexposed | 2,249,556 | 12,913,510 | 7,969 | 1.00 | (ref) |  |
| Exposed | 400,569 | 2,466,989 | 1,863 | 1.11 | 1.02, 1.20 |  |
| Asthma |  |  |  |  |  |  |
| Unexposed | 385,645 | 2,012,479 | 1,523 | 1.00 | (ref) |  |
| Exposed | 146,166 | 841,789 | 588 | 0.96 | 0.73, 1.25 |  |
|  |  |  |  |  |  |  |
| Diseases of the GUS |  |  |  |  |  | 0.12 |
| No asthma |  |  |  |  |  |  |
| Unexposed | 2,249,556 | 12,913,510 | 3,354 | 1.00 | (ref) |  |
| Exposed | 400,569 | 2,466,989 | 795 | 1.05 | 0.92, 1.20 |  |
| Asthma |  |  |  |  |  |  |
| Unexposed | 385,645 | 2,012,479 | 523 | 1.00 | (ref) |  |
| Exposed | 146,166 | 841,789 | 213 | 0.99 | 0.59, 1.68 |  |
|  |  |  |  |  |  |  |
| Other causes |  |  |  |  |  | 0.84 |
| No asthma |  |  |  |  |  |  |
| Unexposed | 2,249,556 | 12,913,510 | 26,656 | 1.00 | (ref) |  |
| Exposed | 400,569 | 2,466,989 | 5,730 | 0.99 | 0.94, 1.04 |  |
| Asthma |  |  |  |  |  |  |
| Unexposed | 385,645 | 2,012,479 | 3,655 | 1.00 | (ref) |  |
| Exposed | 146,166 | 841,789 | 1,252 | 0.94 | 0.78, 1.14 |  |
|  |  |  |  |  |  |  |

^1^Adjusted for current calendar period (1998-2001, 2002-2004, 2005-2007, 2008-2010, 2011-2013, 2014-2016) and Index of Multiple Deprivation at cohort entry.

^2^Estimated hazard ratios from Cox regression with current age as underlying timescale, stratified by matched set (matched on age at cohort entry, gender, date at cohort entry and practice).

GUS: Genitourinary system

# **Table E2**. Association between atopic eczema and all-cause and cause-specific mortality (cause-specific hazards), by sex. n = 3,094,608. Adjusted models^1^.

|  |  |  |  |  | | Interaction |
| --- | --- | --- | --- | --- | --- | --- |
|  | n | P-Y at risk | Events | HR & 99% CI^2^ | | p-value |
| **All-cause mortality** |  |  |  |  |  |  |
|  |  |  |  |  |  |  |
| All-cause mortality |  |  |  |  |  | <0.001 |
| Males |  |  |  |  |  |  |
| Unexposed | 1,079,198 | 6,255,454 | 93,634 | 1.00 | (ref) |  |
| Exposed | 218,702 | 1,338,384 | 24,321 | 1.09 | 1.07, 1.11 |  |
| Females |  |  |  |  |  |  |
| Unexposed | 1,488,674 | 8,670,536 | 106,011 | 1.00 | (ref) |  |
| Exposed | 308,034 | 1,970,394 | 25,193 | 1.00 | 0.98, 1.02 |  |
|  |  |  |  |  |  |  |
| **Cause-specific mortality** |  |  |  |  |  |  |
|  |  |  |  |  |  |  |
| Infections |  |  |  |  |  | 0.01 |
| Males |  |  |  |  |  |  |
| Unexposed | 1,079,198 | 6,255,454 | 847 | 1.00 | (ref) |  |
| Exposed | 218,702 | 1,338,384 | 252 | 1.35 | 1.08, 1.69 |  |
| Females |  |  |  |  |  |  |
| Unexposed | 1,488,674 | 8,670,536 | 1,193 | 1.00 | (ref) |  |
| Exposed | 308,034 | 1,970,394 | 311 | 1.01 | 0.83, 1.23 |  |
|  |  |  |  |  |  |  |
| Neoplasms |  |  |  |  |  | 0.01 |
| Males |  |  |  |  |  |  |
| Unexposed | 1,079,198 | 6,255,454 | 29,094 | 1.00 | (ref) |  |
| Exposed | 218,702 | 1,338,384 | 7,384 | 1.09 | 1.05, 1.13 |  |
| Females |  |  |  |  |  |  |
| Unexposed | 1,488,674 | 8,670,536 | 27,779 | 1.00 | (ref) |  |
| Exposed | 308,034 | 1,970,394 | 6,668 | 1.03 | 0.99, 1.07 |  |
|  |  |  |  |  |  |  |
| Circulatory disease |  |  |  |  |  | 0.01 |
| Males |  |  |  |  |  |  |
| Unexposed | 1,079,198 | 6,255,454 | 33,255 | 1.00 | (ref) |  |
| Exposed | 218,702 | 1,338,384 | 8,401 | 1.07 | 1.03, 1.11 |  |
| Females |  |  |  |  |  |  |
| Unexposed | 1,488,674 | 8,670,536 | 35,414 | 1.00 | (ref) |  |
| Exposed | 308,034 | 1,970,394 | 8,387 | 1.01 | 0.97, 1.04 |  |
|  |  |  |  |  |  |  |
| Respiratory disease |  |  |  |  |  | <0.001 |
| Males |  |  |  |  |  |  |
| Unexposed | 1,079,198 | 6,255,454 | 13,211 | 1.00 | (ref) |  |
| Exposed | 218,702 | 1,338,384 | 3,813 | 1.13 | 1.06, 1.20 |  |
| Females |  |  |  |  |  |  |
| Unexposed | 1,488,674 | 8,670,536 | 15,172 | 1.00 | (ref) |  |
| Exposed | 308,034 | 1,970,394 | 3,857 | 1.00 | 0.95, 1.06 |  |
|  |  |  |  |  |  |  |
| Digestive disease |  |  |  |  |  | 0.003 |
| Males |  |  |  |  |  |  |
| Unexposed | 1,079,198 | 6,255,454 | 4,075 | 1.00 | (ref) |  |
| Exposed | 218,702 | 1,338,384 | 1,102 | 1.22 | 1.10, 1.35 |  |
| Females |  |  |  |  |  |  |
| Unexposed | 1,488,674 | 8,670,536 | 5,417 | 1.00 | (ref) |  |
| Exposed | 308,034 | 1,970,394 | 1,349 | 1.03 | 0.94, 1.13 |  |
|  |  |  |  |  |  |  |
| Diseases of the GUS |  |  |  |  |  | 0.79 |
| Males |  |  |  |  |  |  |
| Unexposed | 1,079,198 | 6,255,454 | 1,621 | 1.00 | (ref) |  |
| Exposed | 218,702 | 1,338,384 | 440 | 1.06 | 0.89, 1.27 |  |
| Females |  |  |  |  |  |  |
| Unexposed | 1,488,674 | 8,670,536 | 2,256 | 1.00 | (ref) |  |
| Exposed | 308,034 | 1,970,394 | 568 | 1.09 | 0.94, 1.26 |  |
|  |  |  |  |  |  |  |
| Other causes |  |  |  |  |  | <0.001 |
| Males |  |  |  |  |  |  |
| Unexposed | 1,079,198 | 6,255,454 | 11,531 | 1.00 | (ref) |  |
| Exposed | 218,702 | 1,338,384 | 2,929 | 1.07 | 1.00, 1.14 |  |
| Females |  |  |  |  |  |  |
| Unexposed | 1,488,674 | 8,670,536 | 18,780 | 1.00 | (ref) |  |
| Exposed | 308,034 | 1,970,394 | 4,053 | 0.94 | 0.88, 0.99 |  |
|  |  |  |  |  |  |  |

^1^Adjusted for current calendar period (1998-2001, 2002-2004, 2005-2007, 2008-2010, 2011-2013, 2014-2016), Index of Multiple Deprivation at cohort entry and time-varying asthma.

^2^Estimated hazard ratios from Cox regression with current age as underlying timescale, stratified by matched set (matched on age at cohort entry, gender, date at cohort entry and practice).

GUS: Genitourinary system

# **Table E3**. Association between atopic eczema and all-cause and cause-specific mortality (cause-specific hazards), by current age. n = 3,094,608. Adjusted models^1^.

|  |  |  |  |  | | Interaction |
| --- | --- | --- | --- | --- | --- | --- |
|  | n | P-Y at risk | Events | HR & 99% CI^2^ | | p-value |
| **All-cause mortality** |  |  |  |  |  |  |
|  |  |  |  |  |  |  |
| All-cause mortality |  |  |  |  |  | <0.001 |
| Age 18-44 years |  |  |  |  |  |  |
| Unexposed | 1,400,027 | 6,085,404 | 3,946 | 1.00 | (ref) |  |
| Exposed | 282,758 | 1,363,465 | 1,027 | 1.13 | 1.02, 1.25 |  |
| Age 45-64 years |  |  |  |  |  |  |
| Unexposed | 906,695 | 4,776,591 | 20,363 | 1.00 | (ref) |  |
| Exposed | 183,603 | 1,014,283 | 5,323 | 1.19 | 1.14, 1.24 |  |
| Age 65+ years |  |  |  |  |  |  |
| Unexposed | 737,385 | 4,063,995 | 175,336 | 1.00 | (ref) |  |
| Exposed | 162,589 | 931,030 | 43,164 | 1.02 | 1.00, 1.04 |  |
|  |  |  |  |  |  |  |
| **Cause-specific mortality** |  |  |  |  |  |  |
|  |  |  |  |  |  |  |
| Infections |  |  |  |  |  | 0.03 |
| Age 18-44 years |  |  |  |  |  |  |
| Unexposed | 1,400,027 | 6,085,404 | 59 | 1.00 | (ref) |  |
| Exposed | 282,758 | 1,363,465 | 21 | 1.82 | 0.86, 3.88 |  |
| Age 45-64 years |  |  |  |  |  |  |
| Unexposed | 906,695 | 4,776,591 | 191 | 1.00 | (ref) |  |
| Exposed | 183,603 | 1,014,283 | 63 | 1.53 | 1.01, 2.33 |  |
| Age 65+ years |  |  |  |  |  |  |
| Unexposed | 737,385 | 4,063,995 | 1,790 | 1.00 | (ref) |  |
| Exposed | 162,589 | 931,030 | 479 | 1.07 | 0.91, 1.26 |  |
|  |  |  |  |  |  |  |
| Neoplasms |  |  |  |  |  | 0.003 |
| Age 18-44 years |  |  |  |  |  |  |
| Unexposed | 1,400,027 | 6,085,404 | 970 | 1.00 | (ref) |  |
| Exposed | 282,758 | 1,363,465 | 265 | 1.20 | 0.98, 1.46 |  |
| Age 45-64 years |  |  |  |  |  |  |
| Unexposed | 906,695 | 4,776,591 | 9,623 | 1.00 | (ref) |  |
| Exposed | 183,603 | 1,014,283 | 2,344 | 1.13 | 1.06, 1.20 |  |
| Age 65+ years |  |  |  |  |  |  |
| Unexposed | 737,385 | 4,063,995 | 46,280 | 1.00 | (ref) |  |
| Exposed | 162,589 | 931,030 | 11,443 | 1.04 | 1.01, 1.07 |  |
|  |  |  |  |  |  |  |
| Circulatory disease |  |  |  |  |  | 0.001 |
| Age 18-44 years |  |  |  |  |  |  |
| Unexposed | 1,400,027 | 6,085,404 | 556 | 1.00 | (ref) |  |
| Exposed | 282,758 | 1,363,465 | 137 | 1.07 | 0.81, 1.42 |  |
| Age 45-64 years |  |  |  |  |  |  |
| Unexposed | 906,695 | 4,776,591 | 4,996 | 1.00 | (ref) |  |
| Exposed | 183,603 | 1,014,283 | 1,232 | 1.15 | 1.05, 1.25 |  |
| Age 65+ years |  |  |  |  |  |  |
| Unexposed | 737,385 | 4,063,995 | 63,117 | 1.00 | (ref) |  |
| Exposed | 162,589 | 931,030 | 15,419 | 1.02 | 1.00, 1.05 |  |
|  |  |  |  |  |  |  |
| Respiratory disease |  |  |  |  |  | <0.001 |
| Age 18-44 years |  |  |  |  |  |  |
| Unexposed | 1,400,027 | 6,085,404 | 141 | 1.00 | (ref) |  |
| Exposed | 282,758 | 1,363,465 | 57 | 1.47 | 0.91, 2.38 |  |
| Age 45-64 years |  |  |  |  |  |  |
| Unexposed | 906,695 | 4,776,591 | 1,404 | 1.00 | (ref) |  |
| Exposed | 183,603 | 1,014,283 | 509 | 1.41 | 1.20, 1.65 |  |
| Age 65+ years |  |  |  |  |  |  |
| Unexposed | 737,385 | 4,063,995 | 26,838 | 1.00 | (ref) |  |
| Exposed | 162,589 | 931,030 | 7,104 | 1.03 | 0.99, 1.08 |  |
|  |  |  |  |  |  |  |
| Digestive disease |  |  |  |  |  | 0.001 |
| Age 18-44 years |  |  |  |  |  |  |
| Unexposed | 1,400,027 | 6,085,404 | 308 | 1.00 | (ref) |  |
| Exposed | 282,758 | 1,363,465 | 88 | 1.25 | 0.88, 1.79 |  |
| Age 45-64 years |  |  |  |  |  |  |
| Unexposed | 906,695 | 4,776,591 | 1,604 | 1.00 | (ref) |  |
| Exposed | 183,603 | 1,014,283 | 456 | 1.33 | 1.15, 1.54 |  |
| Age 65+ years |  |  |  |  |  |  |
| Unexposed | 737,385 | 4,063,995 | 7,580 | 1.00 | (ref) |  |
| Exposed | 162,589 | 931,030 | 1,907 | 1.05 | 0.97, 1.13 |  |
|  |  |  |  |  |  |  |
| Diseases of the GUS |  |  |  |  |  | 0.01 |
| Age 18-44 years |  |  |  |  |  |  |
| Unexposed | 1,400,027 | 6,085,404 | 34 | 1.00 | (ref) |  |
| Exposed | 282,758 | 1,363,465 | 3 | 0.23 | 0.03, 1.92 |  |
| Age 45-64 years |  |  |  |  |  |  |
| Unexposed | 906,695 | 4,776,591 | 111 | 1.00 | (ref) |  |
| Exposed | 183,603 | 1,014,283 | 39 | 1.75 | 1.03, 3.00 |  |
| Age 65+ years |  |  |  |  |  |  |
| Unexposed | 737,385 | 4,063,995 | 3,732 | 1.00 | (ref) |  |
| Exposed | 162,589 | 931,030 | 966 | 1.06 | 0.94, 1.19 |  |
|  |  |  |  |  |  |  |
| Other causes |  |  |  |  |  | <0.001 |
| Age 18-44 years |  |  |  |  |  |  |
| Unexposed | 1,400,027 | 6,085,404 | 1,878 | 1.00 | (ref) |  |
| Exposed | 282,758 | 1,363,465 | 456 | 1.06 | 0.91, 1.23 |  |
| Age 45-64 years |  |  |  |  |  |  |
| Unexposed | 906,695 | 4,776,591 | 2,434 | 1.00 | (ref) |  |
| Exposed | 183,603 | 1,014,283 | 680 | 1.30 | 1.15, 1.47 |  |
| Age 65+ years |  |  |  |  |  |  |
| Unexposed | 737,385 | 4,063,995 | 25,999 | 1.00 | (ref) |  |
| Exposed | 162,589 | 931,030 | 5,846 | 0.94 | 0.90, 0.98 |  |
|  |  |  |  |  |  |  |

^1^Adjusted for current calendar period (1998-2001, 2002-2004, 2005-2007, 2008-2010, 2011-2013, 2014-2016), Index of Multiple Deprivation at cohort entry and time-varying asthma.

^2^Estimated hazard ratios from Cox regression with current age as underlying timescale, stratified by matched set (matched on age at cohort entry, gender, date at cohort entry and practice).

GUS: Genitourinary system

# **Table E4**. Association between atopic eczema severity and all-cause and cause-specific mortality (cause-specific hazards). n = 3,094,608.

|  |  |  |  | HR & 99% CI^1^ | | | |
| --- | --- | --- | --- | --- | --- | --- | --- |
|  | n | P-Y at risk | Events | Unadjusted | | Adjusted | |
| **All-cause mortality** |  |  |  |  |  |  |  |
|  |  |  |  |  |  |  |  |
| All-cause mortality |  |  |  |  |  |  |  |
| Unexposed | 2,567,872 | 14,925,990 | 199,645 | 1.00 | (ref) | 1.00 | (ref) |
| Exposed – low | 385,163 | 2,000,297 | 24,292 | 1.01 | 0.99, 1.03 | 0.98 | 0.96, 1.00 |
| Exposed – moderate | 186,069 | 1,120,570 | 20,776 | 1.08 | 1.05, 1.10 | 1.04 | 1.02, 1.07 |
| Exposed – severe | 34,610 | 187,910 | 4,446 | 1.70 | 1.61, 1.79 | 1.62 | 1.54, 1.71 |
|  |  |  |  |  |  |  |  |
| **Cause-specific mortality** |  |  |  |  |  |  |  |
|  |  |  |  |  |  |  |  |
| Infections |  |  |  |  |  |  |  |
| Unexposed | 2,567,872 | 14,925,990 | 2,040 | 1.00 | (ref) | 1.00 | (ref) |
| Exposed – low | 385,163 | 2,000,297 | 244 | 0.97 | 0.79, 1.20 | 0.95 | 0.76, 1.18 |
| Exposed – moderate | 186,069 | 1,120,570 | 237 | 1.18 | 0.94, 1.48 | 1.15 | 0.91, 1.45 |
| Exposed – severe | 34,610 | 187,910 | 82 | 3.02 | 1.90, 4.80 | 2.85 | 1.78, 4.55 |
|  |  |  |  |  |  |  |  |
| Neoplasms |  |  |  |  |  |  |  |
| Unexposed | 2,567,872 | 14,925,990 | 56,873 | 1.00 | (ref) | 1.00 | (ref) |
| Exposed – low | 385,163 | 2,000,297 | 7,449 | 1.09 | 1.05, 1.13 | 1.07 | 1.03, 1.11 |
| Exposed – moderate | 186,069 | 1,120,570 | 5,607 | 1.03 | 0.98, 1.07 | 1.01 | 0.97, 1.05 |
| Exposed – severe | 34,610 | 187,910 | 996 | 1.31 | 1.18, 1.45 | 1.27 | 1.15, 1.42 |
|  |  |  |  |  |  |  |  |
| Circulatory disease |  |  |  |  |  |  |  |
| Unexposed | 2,567,872 | 14,925,990 | 68,669 | 1.00 | (ref) | 1.00 | (ref) |
| Exposed – low | 385,163 | 2,000,297 | 7,981 | 0.97 | 0.93, 1.00 | 0.95 | 0.91, 0.98 |
| Exposed – moderate | 186,069 | 1,120,570 | 7,379 | 1.10 | 1.06, 1.15 | 1.08 | 1.04, 1.12 |
| Exposed – severe | 34,610 | 187,910 | 1,428 | 1.58 | 1.44, 1.74 | 1.53 | 1.39, 1.68 |
|  |  |  |  |  |  |  |  |
| Respiratory disease |  |  |  |  |  |  |  |
| Unexposed | 2,567,872 | 14,925,990 | 28,383 | 1.00 | (ref) | 1.00 | (ref) |
| Exposed – low | 385,163 | 2,000,297 | 3,585 | 1.03 | 0.98, 1.10 | 0.94 | 0.88, 1.00 |
| Exposed – moderate | 186,069 | 1,120,570 | 3,210 | 1.16 | 1.09, 1.24 | 1.06 | 1.00, 1.13 |
| Exposed – severe | 34,610 | 187,910 | 875 | 2.43 | 2.13, 2.78 | 2.20 | 1.91, 2.53 |
|  |  |  |  |  |  |  |  |
| Digestive disease |  |  |  |  |  |  |  |
| Unexposed | 2,567,872 | 14,925,990 | 9,492 | 1.00 | (ref) | 1.00 | (ref) |
| Exposed – low | 385,163 | 2,000,297 | 1,138 | 0.99 | 0.90, 1.10 | 0.97 | 0.88, 1.07 |
| Exposed – moderate | 186,069 | 1,120,570 | 1,063 | 1.21 | 1.09, 1.34 | 1.16 | 1.05, 1.29 |
| Exposed – severe | 34,610 | 187,910 | 250 | 1.95 | 1.55, 2.45 | 1.88 | 1.49, 2.36 |
|  |  |  |  |  |  |  |  |
| Diseases of the GUS |  |  |  |  |  |  |  |
| Unexposed | 2,567,872 | 14,925,990 | 3,877 | 1.00 | (ref) | 1.00 | (ref) |
| Exposed – low | 385,163 | 2,000,297 | 451 | 0.98 | 0.83, 1.15 | 0.96 | 0.81, 1.13 |
| Exposed – moderate | 186,069 | 1,120,570 | 438 | 1.10 | 0.92, 1.31 | 1.08 | 0.90, 1.28 |
| Exposed – severe | 34,610 | 187,910 | 119 | 2.18 | 1.49, 3.19 | 2.10 | 1.43, 3.07 |
|  |  |  |  |  |  |  |  |
| Other causes |  |  |  |  |  |  |  |
| Unexposed | 2,567,872 | 14,925,990 | 30,311 | 1.00 | (ref) | 1.00 | (ref) |
| Exposed – low | 385,163 | 2,000,297 | 3,444 | 0.93 | 0.88, 0.99 | 0.92 | 0.87, 0.98 |
| Exposed – moderate | 186,069 | 1,120,570 | 2,842 | 0.96 | 0.90, 1.03 | 0.95 | 0.89, 1.02 |
| Exposed – severe | 34,610 | 187,910 | 696 | 1.93 | 1.67, 2.22 | 1.91 | 1.66, 2.21 |
|  |  |  |  |  |  |  |  |

^1^Estimated hazard ratios from Cox regression with current age as underlying timescale, stratified by matched set (matched on age at cohort entry, gender, date at cohort entry and practice).

GUS: Genitourinary system

Unadjusted: No adjustment.

Adjusted: Adjusted for current calendar period (1998-2001, 2002-2004, 2005-2007, 2008-2010, 2011-2013, 2014-2016), Index of Multiple Deprivation at cohort entry and time-varying asthma.

# **Table E5**. Association between atopic eczema activity and all-cause and cause-specific mortality (cause-specific hazards), by activity of atopic eczema (assessed during the first twelve months of follow-up, assuming active eczema for three months after a single health care contact), excluding the first 12 months of follow-up. n = 2,614,344.

|  |  |  |  | HR & 99% CI^1^ | | | |
| --- | --- | --- | --- | --- | --- | --- | --- |
|  | n | P-Y at risk | Events | Unadjusted | | Adjusted | |
| **All-cause mortality** |  |  |  |  |  |  |  |
|  |  |  |  |  |  |  |  |
| All-cause mortality |  |  |  |  |  |  |  |
| Unexposed | 2,158,956 | 12,571,608 | 170,953 | 1.00 | (ref) | 1.00 | (ref) |
| Exposed – never active | 167,831 | 1,047,613 | 12,672 | 0.99 | 0.96, 1.03 | 0.97 | 0.94, 1.00 |
| Exposed – <50% active | 202,791 | 1,270,652 | 15,790 | 0.98 | 0.96, 1.01 | 0.96 | 0.93, 0.98 |
| Exposed – 50+% active | 84,766 | 500,615 | 14,101 | 1.32 | 1.28, 1.36 | 1.26 | 1.22, 1.29 |
|  |  |  |  |  |  |  |  |
| **Cause-specific mortality** |  |  |  |  |  |  |  |
|  |  |  |  |  |  |  |  |
| Infections |  |  |  |  |  |  |  |
| Unexposed | 2,158,956 | 12,571,608 | 1,800 | 1.00 | (ref) | 1.00 | (ref) |
| Exposed – never active | 167,831 | 1,047,613 | 132 | 0.90 | 0.67, 1.21 | 0.89 | 0.66, 1.20 |
| Exposed – <50% active | 202,791 | 1,270,652 | 176 | 1.10 | 0.85, 1.42 | 1.07 | 0.82, 1.38 |
| Exposed – 50+% active | 84,766 | 500,615 | 186 | 1.60 | 1.22, 2.09 | 1.54 | 1.17, 2.03 |
|  |  |  |  |  |  |  |  |
| Neoplasms |  |  |  |  |  |  |  |
| Unexposed | 2,158,956 | 12,571,608 | 49,758 | 1.00 | (ref) | 1.00 | (ref) |
| Exposed – never active | 167,831 | 1,047,613 | 3,730 | 1.02 | 0.97, 1.08 | 1.01 | 0.96, 1.07 |
| Exposed – <50% active | 202,791 | 1,270,652 | 4,917 | 1.01 | 0.96, 1.06 | 1.00 | 0.95, 1.04 |
| Exposed – 50+% active | 84,766 | 500,615 | 3,317 | 1.15 | 1.08, 1.21 | 1.11 | 1.05, 1.18 |
|  |  |  |  |  |  |  |  |
| Circulatory disease |  |  |  |  |  |  |  |
| Unexposed | 2,158,956 | 12,571,608 | 58,350 | 1.00 | (ref) | 1.00 | (ref) |
| Exposed – never active | 167,831 | 1,047,613 | 4,402 | 1.02 | 0.97, 1.08 | 1.01 | 0.95, 1.06 |
| Exposed – <50% active | 202,791 | 1,270,652 | 5,253 | 0.96 | 0.92, 1.01 | 0.94 | 0.90, 0.99 |
| Exposed – 50+% active | 84,766 | 500,615 | 4,821 | 1.29 | 1.23, 1.36 | 1.25 | 1.19, 1.32 |
|  |  |  |  |  |  |  |  |
| Respiratory disease |  |  |  |  |  |  |  |
| Unexposed | 2,158,956 | 12,571,608 | 24,233 | 1.00 | (ref) | 1.00 | (ref) |
| Exposed – never active | 167,831 | 1,047,613 | 1,776 | 0.95 | 0.88, 1.04 | 0.89 | 0.81, 0.97 |
| Exposed – <50% active | 202,791 | 1,270,652 | 2,297 | 1.04 | 0.96, 1.11 | 0.97 | 0.90, 1.04 |
| Exposed – 50+% active | 84,766 | 500,615 | 2,626 | 1.68 | 1.56, 1.80 | 1.45 | 1.34, 1.56 |
|  |  |  |  |  |  |  |  |
| Digestive disease |  |  |  |  |  |  |  |
| Unexposed | 2,158,956 | 12,571,608 | 8,270 | 1.00 | (ref) | 1.00 | (ref) |
| Exposed – never active | 167,831 | 1,047,613 | 656 | 1.04 | 0.91, 1.19 | 1.02 | 0.89, 1.16 |
| Exposed – <50% active | 202,791 | 1,270,652 | 794 | 1.04 | 0.93, 1.17 | 1.02 | 0.90, 1.14 |
| Exposed – 50+% active | 84,766 | 500,615 | 683 | 1.40 | 1.23, 1.60 | 1.35 | 1.18, 1.54 |
|  |  |  |  |  |  |  |  |
| Diseases of the GUS |  |  |  |  |  |  |  |
| Unexposed | 2,158,956 | 12,571,608 | 3,368 | 1.00 | (ref) | 1.00 | (ref) |
| Exposed – never active | 167,831 | 1,047,613 | 250 | 1.03 | 0.82, 1.31 | 1.02 | 0.80, 1.29 |
| Exposed – <50% active | 202,791 | 1,270,652 | 290 | 0.85 | 0.69, 1.05 | 0.84 | 0.68, 1.03 |
| Exposed – 50+% active | 84,766 | 500,615 | 338 | 1.55 | 1.26, 1.90 | 1.49 | 1.22, 1.83 |
|  |  |  |  |  |  |  |  |
| Other causes |  |  |  |  |  |  |  |
| Unexposed | 2,158,956 | 12,571,608 | 25,174 | 1.00 | (ref) | 1.00 | (ref) |
| Exposed – never active | 167,831 | 1,047,613 | 1,726 | 0.89 | 0.82, 0.97 | 0.89 | 0.81, 0.97 |
| Exposed – <50% active | 202,791 | 1,270,652 | 2,063 | 0.90 | 0.83, 0.97 | 0.89 | 0.83, 0.96 |
| Exposed – 50+% active | 84,766 | 500,615 | 2,130 | 1.32 | 1.22, 1.44 | 1.30 | 1.20, 1.41 |
|  |  |  |  |  |  |  |  |

^1^Estimated hazard ratios from Cox regression with current age as underlying timescale, stratified by matched set (matched on age at cohort entry, gender, date at cohort entry and practice).

GUS: Genitourinary system

Unadjusted: No adjustment.

Adjusted: Adjusted for current calendar period (1998-2001, 2002-2004, 2005-2007, 2008-2010, 2011-2013, 2014-2016), Index of Multiple Deprivation at cohort entry and time-varying asthma.

# **Table E6**. Association between atopic eczema and all-cause and cause-specific mortality (cause-specific hazards), with exposed patients restricted to those with incident atopic eczema. Fitted to individuals from valid matched sets^1^. n = 1,552,870.

|  |  |  |  | HR & 99% CI^2^ | | | |
| --- | --- | --- | --- | --- | --- | --- | --- |
|  | n | P-Y at risk | Events | Unadjusted | | Adjusted | |
| **All-cause mortality** |  |  |  |  |  |  |  |
|  |  |  |  |  |  |  |  |
| All-cause mortality |  |  |  |  |  |  |  |
| Unexposed | 1,285,804 | 7,008,930 | 102,664 | 1.00 | (ref) | 1.00 | (ref) |
| Exposed | 267,066 | 1,588,462 | 25,792 | 1.02 | 1.00, 1.04 | 1.00 | 0.97, 1.02 |
|  |  |  |  |  |  |  |  |
| **Cause-specific mortality** |  |  |  |  |  |  |  |
|  |  |  |  |  |  |  |  |
| Infections |  |  |  |  |  |  |  |
| Unexposed | 1,285,804 | 7,008,930 | 1,111 | 1.00 | (ref) | 1.00 | (ref) |
| Exposed | 267,066 | 1,588,462 | 309 | 1.14 | 0.94, 1.39 | 1.12 | 0.91, 1.36 |
|  |  |  |  |  |  |  |  |
| Neoplasms |  |  |  |  |  |  |  |
| Unexposed | 1,285,804 | 7,008,930 | 29,713 | 1.00 | (ref) | 1.00 | (ref) |
| Exposed | 267,066 | 1,588,462 | 7,817 | 1.08 | 1.04, 1.12 | 1.07 | 1.03, 1.11 |
|  |  |  |  |  |  |  |  |
| Circulatory disease |  |  |  |  |  |  |  |
| Unexposed | 1,285,804 | 7,008,930 | 33,648 | 1.00 | (ref) | 1.00 | (ref) |
| Exposed | 267,066 | 1,588,462 | 8,376 | 1.00 | 0.96, 1.04 | 0.98 | 0.94, 1.02 |
|  |  |  |  |  |  |  |  |
| Respiratory disease |  |  |  |  |  |  |  |
| Unexposed | 1,285,804 | 7,008,930 | 14,401 | 1.00 | (ref) | 1.00 | (ref) |
| Exposed | 267,066 | 1,588,462 | 3,879 | 1.08 | 1.02, 1.14 | 0.99 | 0.93, 1.05 |
|  |  |  |  |  |  |  |  |
| Digestive disease |  |  |  |  |  |  |  |
| Unexposed | 1,285,804 | 7,008,930 | 4,860 | 1.00 | (ref) | 1.00 | (ref) |
| Exposed | 267,066 | 1,588,462 | 1,305 | 1.11 | 1.01, 1.22 | 1.08 | 0.98, 1.19 |
|  |  |  |  |  |  |  |  |
| Diseases of the GUS |  |  |  |  |  |  |  |
| Unexposed | 1,285,804 | 7,008,930 | 2,051 | 1.00 | (ref) | 1.00 | (ref) |
| Exposed | 267,066 | 1,588,462 | 532 | 1.00 | 0.85, 1.17 | 0.98 | 0.83, 1.14 |
|  |  |  |  |  |  |  |  |
| Other causes |  |  |  |  |  |  |  |
| Unexposed | 1,285,804 | 7,008,930 | 16,880 | 1.00 | (ref) | 1.00 | (ref) |
| Exposed | 267,066 | 1,588,462 | 3,574 | 0.87 | 0.82, 0.92 | 0.86 | 0.81, 0.92 |
|  |  |  |  |  |  |  |  |

^1^Matched sets including one exposed person and at least one unexposed person.

^2^Estimated hazard ratios from Cox regression with current age as underlying timescale, stratified by matched set (matched on age at cohort entry, gender, date at cohort entry and practice).

GUS: Genitourinary system

Unadjusted: No adjustment.

Adjusted: Adjusted for current calendar period (1998-2001, 2002-2004, 2005-2007, 2008-2010, 2011-2013, 2014-2016), Index of Multiple Deprivation at cohort entry and time-varying asthma.

# **Table E7**. Association between atopic eczema and all-cause and cause-specific mortality (cause-specific hazards) restricted to people with at least one consultation with their GP in the year prior to cohort entry. Fitted to individuals from valid matched sets^1^. n = 2,478,735.

|  |  |  |  | HR & 99% CI^2^ | | | |
| --- | --- | --- | --- | --- | --- | --- | --- |
|  | n | P-Y at risk | Events | Unadjusted | | Adjusted | |
| **All-cause mortality** |  |  |  |  |  |  |  |
|  |  |  |  |  |  |  |  |
| All-cause mortality |  |  |  |  |  |  |  |
| Unexposed | 1,981,046 | 11,628,862 | 179,712 | 1.00 | (ref) | 1.00 | (ref) |
| Exposed | 497,689 | 3,132,853 | 48,515 | 1.04 | 1.02, 1.06 | 1.01 | 1.00, 1.03 |
|  |  |  |  |  |  |  |  |
| **Cause-specific mortality** |  |  |  |  |  |  |  |
|  |  |  |  |  |  |  |  |
| Infections |  |  |  |  |  |  |  |
| Unexposed | 1,981,046 | 11,628,862 | 1,881 | 1.00 | (ref) | 1.00 | (ref) |
| Exposed | 497,689 | 3,132,853 | 553 | 1.11 | 0.95, 1.29 | 1.08 | 0.93, 1.26 |
|  |  |  |  |  |  |  |  |
| Neoplasms |  |  |  |  |  |  |  |
| Unexposed | 1,981,046 | 11,628,862 | 50,023 | 1.00 | (ref) | 1.00 | (ref) |
| Exposed | 497,689 | 3,132,853 | 13,756 | 1.05 | 1.02, 1.08 | 1.04 | 1.01, 1.07 |
|  |  |  |  |  |  |  |  |
| Circulatory disease |  |  |  |  |  |  |  |
| Unexposed | 1,981,046 | 11,628,862 | 62,022 | 1.00 | (ref) | 1.00 | (ref) |
| Exposed | 497,689 | 3,132,853 | 16,472 | 1.03 | 1.00, 1.06 | 1.01 | 0.98, 1.04 |
|  |  |  |  |  |  |  |  |
| Respiratory disease |  |  |  |  |  |  |  |
| Unexposed | 1,981,046 | 11,628,862 | 26,127 | 1.00 | (ref) | 1.00 | (ref) |
| Exposed | 497,689 | 3,132,853 | 7,527 | 1.11 | 1.07, 1.16 | 1.03 | 0.98, 1.07 |
|  |  |  |  |  |  |  |  |
| Digestive disease |  |  |  |  |  |  |  |
| Unexposed | 1,981,046 | 11,628,862 | 8,445 | 1.00 | (ref) | 1.00 | (ref) |
| Exposed | 497,689 | 3,132,853 | 2,377 | 1.09 | 1.01, 1.16 | 1.06 | 0.99, 1.14 |
|  |  |  |  |  |  |  |  |
| Diseases of the GUS |  |  |  |  |  |  |  |
| Unexposed | 1,981,046 | 11,628,862 | 3,567 | 1.00 | (ref) | 1.00 | (ref) |
| Exposed | 497,689 | 3,132,853 | 990 | 1.04 | 0.92, 1.17 | 1.02 | 0.90, 1.14 |
|  |  |  |  |  |  |  |  |
| Other causes |  |  |  |  |  |  |  |
| Unexposed | 1,981,046 | 11,628,862 | 27,647 | 1.00 | (ref) | 1.00 | (ref) |
| Exposed | 497,689 | 3,132,853 | 6,840 | 0.95 | 0.91, 0.99 | 0.95 | 0.91, 0.99 |
|  |  |  |  |  |  |  |  |

^1^Matched sets including one exposed person and at least one unexposed person.

^2^Estimated hazard ratios from Cox regression with current age as underlying timescale, stratified by matched set (matched on age at cohort entry, gender, date at cohort entry and practice).

GUS: Genitourinary system

Unadjusted: No adjustment.

Adjusted: Adjusted for current calendar period (2006-2010, 2011-2013, 2014-2016), Index of Multiple Deprivation at cohort entry and time-varying asthma.

# **Table E8**. Association between atopic eczema and all-cause and cause-specific mortality (cause-specific hazards), using first redefined cohort^1^. n = 3,132,843.

|  |  |  |  | HR & 99% CI^2^ | | | |
| --- | --- | --- | --- | --- | --- | --- | --- |
|  | n | P-Y at risk | Events | Unadjusted | | Adjusted | |
| **All-cause mortality** |  |  |  |  |  |  |  |
|  |  |  |  |  |  |  |  |
| All-cause mortality |  |  |  |  |  |  |  |
| Unexposed | 2,601,730 | 14,969,463 | 199,120 | 1.00 | (ref) | 1.00 | (ref) |
| Exposed | 531,113 | 3,326,791 | 49,805 | 1.09 | 1.07, 1.10 | 1.05 | 1.04, 1.07 |
|  |  |  |  |  |  |  |  |
| **Cause-specific mortality** |  |  |  |  |  |  |  |
|  |  |  |  |  |  |  |  |
| Infections |  |  |  |  |  |  |  |
| Unexposed | 2,601,730 | 14,969,463 | 2,042 | 1.00 | (ref) | 1.00 | (ref) |
| Exposed | 531,113 | 3,326,791 | 566 | 1.18 | 1.02, 1.37 | 1.15 | 0.99, 1.33 |
|  |  |  |  |  |  |  |  |
| Neoplasms |  |  |  |  |  |  |  |
| Unexposed | 2,601,730 | 14,969,463 | 56,853 | 1.00 | (ref) | 1.00 | (ref) |
| Exposed | 531,113 | 3,326,791 | 14,126 | 1.08 | 1.06, 1.11 | 1.07 | 1.04, 1.10 |
|  |  |  |  |  |  |  |  |
| Circulatory disease |  |  |  |  |  |  |  |
| Unexposed | 2,601,730 | 14,969,463 | 68,556 | 1.00 | (ref) | 1.00 | (ref) |
| Exposed | 531,113 | 3,326,791 | 16,880 | 1.07 | 1.04, 1.10 | 1.05 | 1.02, 1.08 |
|  |  |  |  |  |  |  |  |
| Respiratory disease |  |  |  |  |  |  |  |
| Unexposed | 2,601,730 | 14,969,463 | 28,225 | 1.00 | (ref) | 1.00 | (ref) |
| Exposed | 531,113 | 3,326,791 | 7,716 | 1.17 | 1.13, 1.22 | 1.07 | 1.02, 1.11 |
|  |  |  |  |  |  |  |  |
| Digestive disease |  |  |  |  |  |  |  |
| Unexposed | 2,601,730 | 14,969,463 | 9,375 | 1.00 | (ref) | 1.00 | (ref) |
| Exposed | 531,113 | 3,326,791 | 2,462 | 1.13 | 1.06, 1.21 | 1.10 | 1.03, 1.18 |
|  |  |  |  |  |  |  |  |
| Diseases of the GUS |  |  |  |  |  |  |  |
| Unexposed | 2,601,730 | 14,969,463 | 3,862 | 1.00 | (ref) | 1.00 | (ref) |
| Exposed | 531,113 | 3,326,791 | 1,013 | 1.09 | 0.98, 1.22 | 1.08 | 0.97, 1.21 |
|  |  |  |  |  |  |  |  |
| Other causes |  |  |  |  |  |  |  |
| Unexposed | 2,601,730 | 14,969,463 | 30,207 | 1.00 | (ref) | 1.00 | (ref) |
| Exposed | 531,113 | 3,326,791 | 7,042 | 1.01 | 0.97, 1.05 | 1.00 | 0.96, 1.04 |
|  |  |  |  |  |  |  |  |

^1^People with an atopic eczema diagnosis but without two further treatments are included in the unexposed pool for the entire duration of their follow-up. People in the exposed cohort (diagnosis and two treatments) are included as unexposed, up until their cohort entry (i.e. latest of their diagnosis and two treatments).  The pool of exposed patients is the same as the main analysis above.

^2^Estimated hazard ratios from Cox regression with current age as underlying timescale, stratified by matched set (matched on age at cohort entry, gender, date at cohort entry and practice).

GUS: Genitourinary system

Unadjusted: No adjustment.

Adjusted: Adjusted for current calendar period (1998-2001, 2002-2004, 2005-2007, 2008-2010, 2011-2013, 2014-2016), Index of Multiple Deprivation at cohort entry and time-varying asthma.

# **Table E9**. Association between atopic eczema and all-cause and cause-specific mortality (cause-specific hazards), using second redefined cohort^1^. n = 3,782,266.

|  |  |  |  | HR & 99% CI^2^ | | | |
| --- | --- | --- | --- | --- | --- | --- | --- |
|  | n | P-Y at risk | Events | Unadjusted | | Adjusted | |
| **All-cause mortality** |  |  |  |  |  |  |  |
|  |  |  |  |  |  |  |  |
| All-cause mortality |  |  |  |  |  |  |  |
| Unexposed | 3,128,246 | 18,617,195 | 216,893 | 1.00 | (ref) | 1.00 | (ref) |
| Exposed | 654,020 | 4,205,054 | 52,479 | 1.02 | 1.01, 1.04 | 1.00 | 0.98, 1.01 |
|  |  |  |  |  |  |  |  |
| **Cause-specific mortality** |  |  |  |  |  |  |  |
|  |  |  |  |  |  |  |  |
| Infections |  |  |  |  |  |  |  |
| Unexposed | 3,128,246 | 18,617,195 | 2,231 | 1.00 | (ref) | 1.00 | (ref) |
| Exposed | 654,020 | 4,205,054 | 600 | 1.11 | 0.96, 1.28 | 1.10 | 0.95, 1.27 |
|  |  |  |  |  |  |  |  |
| Neoplasms |  |  |  |  |  |  |  |
| Unexposed | 3,128,246 | 18,617,195 | 62,497 | 1.00 | (ref) | 1.00 | (ref) |
| Exposed | 654,020 | 4,205,054 | 14,859 | 1.01 | 0.98, 1.03 | 1.00 | 0.97, 1.02 |
|  |  |  |  |  |  |  |  |
| Circulatory disease |  |  |  |  |  |  |  |
| Unexposed | 3,128,246 | 18,617,195 | 74,292 | 1.00 | (ref) | 1.00 | (ref) |
| Exposed | 654,020 | 4,205,054 | 17,882 | 1.02 | 1.00, 1.05 | 1.01 | 0.98, 1.03 |
|  |  |  |  |  |  |  |  |
| Respiratory disease |  |  |  |  |  |  |  |
| Unexposed | 3,128,246 | 18,617,195 | 30,573 | 1.00 | (ref) | 1.00 | (ref) |
| Exposed | 654,020 | 4,205,054 | 7,898 | 1.08 | 1.04, 1.12 | 1.00 | 0.96, 1.04 |
|  |  |  |  |  |  |  |  |
| Digestive disease |  |  |  |  |  |  |  |
| Unexposed | 3,128,246 | 18,617,195 | 10,334 | 1.00 | (ref) | 1.00 | (ref) |
| Exposed | 654,020 | 4,205,054 | 2,601 | 1.08 | 1.01, 1.15 | 1.06 | 1.00, 1.14 |
|  |  |  |  |  |  |  |  |
| Diseases of the GUS |  |  |  |  |  |  |  |
| Unexposed | 3,128,246 | 18,617,195 | 4,085 | 1.00 | (ref) | 1.00 | (ref) |
| Exposed | 654,020 | 4,205,054 | 1,058 | 1.02 | 0.91, 1.14 | 1.00 | 0.89, 1.12 |
|  |  |  |  |  |  |  |  |
| Other causes |  |  |  |  |  |  |  |
| Unexposed | 3,128,246 | 18,617,195 | 32,881 | 1.00 | (ref) | 1.00 | (ref) |
| Exposed | 654,020 | 4,205,054 | 7,581 | 0.97 | 0.93, 1.01 | 0.97 | 0.93, 1.01 |
|  |  |  |  |  |  |  |  |

^1^Exposed people are atopic eczema diagnosis only (without the treatment criteria), and these patients are eligible to be a control up until their atopic eczema diagnosis.

^2^Estimated hazard ratios from Cox regression with current age as underlying timescale, stratified by matched set (matched on age at cohort entry, gender, date at cohort entry and practice).

GUS: Genitourinary system

Unadjusted: No adjustment.

Adjusted: Adjusted for current calendar period (1998-2001, 2002-2004, 2005-2007, 2008-2010, 2011-2013, 2014-2016), Index of Multiple Deprivation at cohort entry and time-varying asthma.

# **Table E10**. Association between atopic eczema and cardiovascular outcomes, using only people registered from 2006 onwards with complete ethnicity data. Fitted to individuals from valid matched sets^1^. n = 339,734.

|  |  |  |  | HR & 99% CI^2^ | | | |
| --- | --- | --- | --- | --- | --- | --- | --- |
|  |  |  |  | Adjusted | | | |
|  | n | P-Y at risk | Events | No adjustment  for ethnicity | | Adjusted for  ethnicity | |
| **All-cause mortality** |  |  |  |  |  |  |  |
|  |  |  |  |  |  |  |  |
| All-cause mortality |  |  |  |  |  |  |  |
| Unexposed | 260,475 | 588,874 | 4,976 | 1.00 | (ref) | 1.00 | (ref) |
| Exposed | 79,259 | 196,739 | 2,125 | 1.09 | 0.98, 1.21 | 1.09 | 0.99, 1.22 |
|  |  |  |  |  |  |  |  |
| **Cause-specific mortality** |  |  |  |  |  |  |  |
|  |  |  |  |  |  |  |  |
| Infections |  |  |  |  |  |  |  |
| Unexposed | 260,475 | 588,874 | 46 | 1.00 | (ref) | 1.00 | (ref) |
| Exposed | 79,259 | 196,739 | 24 | 0.87 | 0.24, 3.25 | 0.99 | 0.26, 3.69 |
|  |  |  |  |  |  |  |  |
| Neoplasms |  |  |  |  |  |  |  |
| Unexposed | 260,475 | 588,874 | 870 | 1.00 | (ref) | 1.00 | (ref) |
| Exposed | 79,259 | 196,739 | 415 | 1.17 | 0.94, 1.45 | 1.18 | 0.94, 1.47 |
|  |  |  |  |  |  |  |  |
| Circulatory disease |  |  |  |  |  |  |  |
| Unexposed | 260,475 | 588,874 | 1,333 | 1.00 | (ref) | 1.00 | (ref) |
| Exposed | 79,259 | 196,739 | 557 | 1.12 | 0.90, 1.37 | 1.12 | 0.91, 1.38 |
|  |  |  |  |  |  |  |  |
| Respiratory disease |  |  |  |  |  |  |  |
| Unexposed | 260,475 | 588,874 | 718 | 1.00 | (ref) | 1.00 | (ref) |
| Exposed | 79,259 | 196,739 | 355 | 1.20 | 0.91, 1.59 | 1.17 | 0.88, 1.55 |
|  |  |  |  |  |  |  |  |
| Digestive disease |  |  |  |  |  |  |  |
| Unexposed | 260,475 | 588,874 | 179 | 1.00 | (ref) | 1.00 | (ref) |
| Exposed | 79,259 | 196,739 | 96 | 1.70 | 1.04, 2.79 | 1.75 | 1.05, 2.90 |
|  |  |  |  |  |  |  |  |
| Diseases of the GUS |  |  |  |  |  |  |  |
| Unexposed | 260,475 | 588,874 | 109 | 1.00 | (ref) | 1.00 | (ref) |
| Exposed | 79,259 | 196,739 | 43 | 0.89 | 0.43, 1.87 | 0.91 | 0.43, 1.90 |
|  |  |  |  |  |  |  |  |
| Other causes |  |  |  |  |  |  |  |
| Unexposed | 260,475 | 588,874 | 1,721 | 1.00 | (ref) | 1.00 | (ref) |
| Exposed | 79,259 | 196,739 | 635 | 0.91 | 0.75, 1.11 | 0.92 | 0.76, 1.13 |
|  |  |  |  |  |  |  |  |

^1^Matched sets including one exposed person and at least one unexposed person.

^2^Estimated hazard ratios from Cox regression with current age as underlying timescale, stratified by matched set (matched on age at cohort entry, gender, date at cohort entry and practice).

^3^Exploratory analysis additionally adjusting for potential mediators.

^4^Problems with model convergence. Results not reported.

GUS: Genitourinary system

Adjusted: Adjusted for current calendar period (2006-2010, 2011-2013, 2014-2016), Index of Multiple Deprivation at cohort entry and time-varying asthma.

Mediation: Adjusted additionally for BMI and smoking at cohort entry, and time-varying depression, anxiety, diabetes and harmful alcohol use.

# **Table E11**. Association between atopic eczema and all-cause and cause-specific mortality (cause-specific hazards), treating time since diagnosis as a time-varying exposure. n = 3,094,608.

|  |  |  |  | HR & 99% CI^1^ | | | |
| --- | --- | --- | --- | --- | --- | --- | --- |
|  | n | P-Y at risk | Events | Unadjusted | | Adjusted | |
| **All-cause mortality** |  |  |  |  |  |  |  |
|  |  |  |  |  |  |  |  |
| All-cause mortality |  |  |  |  |  |  |  |
| Unexposed | 2,567,872 | 14,925,990 | 199,645 | 1.00 | (ref) | 1.00 | (ref) |
| Exposed |  |  |  |  |  |  |  |
| 0-4 years since diagnosis | 307,032 | 941,496 | 15,558 | 1.03 | 1.00, 1.06 | 0.99 | 0.97, 1.02 |
| 5-9 years since diagnosis | 270,911 | 850,439 | 13,724 | 1.10 | 1.07, 1.13 | 1.07 | 1.04, 1.10 |
| 10+ years since diagnosis | 282,808 | 1,516,843 | 20,232 | 1.10 | 1.08, 1.13 | 1.07 | 1.04, 1.09 |
|  |  |  |  |  |  |  |  |
| **Cause-specific mortality** |  |  |  |  |  |  |  |
|  |  |  |  |  |  |  |  |
| Infections |  |  |  |  |  |  |  |
| Unexposed | 2,567,872 | 14,925,990 | 2040 | 1.00 | (ref) | 1.00 | (ref) |
| Exposed |  |  |  |  |  |  |  |
| 0-4 years since diagnosis | 307,032 | 941,496 | 176 | 1.18 | 0.91, 1.52 | 1.14 | 0.87, 1.48 |
| 5-9 years since diagnosis | 270,911 | 850,439 | 133 | 1.04 | 0.78, 1.39 | 1.01 | 0.75, 1.35 |
| 10+ years since diagnosis | 282,808 | 1,516,843 | 254 | 1.26 | 1.00, 1.57 | 1.23 | 0.98, 1.54 |
|  |  |  |  |  |  |  |  |
| Neoplasms |  |  |  |  |  |  |  |
| Unexposed | 2,567,872 | 14,925,990 | 56873 | 1.00 | (ref) | 1.00 | (ref) |
| Exposed |  |  |  |  |  |  |  |
| 0-4 years since diagnosis | 307,032 | 941,496 | 4350 | 1.07 | 1.02, 1.13 | 1.06 | 1.00, 1.11 |
| 5-9 years since diagnosis | 270,911 | 850,439 | 3819 | 1.05 | 1.00, 1.11 | 1.04 | 0.98, 1.09 |
| 20+ years since diagnosis | 282,808 | 1,516,843 | 5883 | 1.10 | 1.05, 1.14 | 1.08 | 1.03, 1.12 |
|  |  |  |  |  |  |  |  |
| Circulatory disease |  |  |  |  |  |  |  |
| Unexposed | 2,567,872 | 14,925,990 | 68669 | 1.00 | (ref) | 1.00 | (ref) |
| Exposed |  |  |  |  |  |  |  |
| 0-4 years since diagnosis | 307,032 | 941,496 | 5384 | 1.03 | 0.98, 1.07 | 0.99 | 0.95, 1.04 |
| 5-9 years since diagnosis | 270,911 | 850,439 | 4812 | 1.10 | 1.05, 1.15 | 1.09 | 1.04, 1.14 |
| 10+ years since diagnosis | 282,808 | 1,516,843 | 6592 | 1.06 | 1.01, 1.10 | 1.04 | 0.99, 1.08 |
|  |  |  |  |  |  |  |  |
| Respiratory disease |  |  |  |  |  |  |  |
| Unexposed | 2,567,872 | 14,925,990 | 28383 | 1.00 | (ref) | 1.00 | (ref) |
| Exposed |  |  |  |  |  |  |  |
| 0-4 years since diagnosis | 307,032 | 941,496 | 2316 | 1.06 | 0.99, 1.14 | 0.95 | 0.88, 1.03 |
| 5-9 years since diagnosis | 270,911 | 850,439 | 2174 | 1.18 | 1.10, 1.27 | 1.10 | 1.02, 1.19 |
| 20+ years since diagnosis | 282,808 | 1,516,843 | 3180 | 1.26 | 1.18, 1.34 | 1.13 | 1.06, 1.21 |
|  |  |  |  |  |  |  |  |
| Digestive disease |  |  |  |  |  |  |  |
| Unexposed | 2,567,872 | 14,925,990 | 9492 | 1.00 | (ref) | 1.00 | (ref) |
| Exposed |  |  |  |  |  |  |  |
| 0-4 years since diagnosis | 307,032 | 941,496 | 757 | 1.11 | 0.93, 1.25 | 1.07 | 0.95, 1.21 |
| 5-9 years since diagnosis | 270,911 | 850,439 | 685 | 1.19 | 1.05, 1.36 | 1.17 | 1.03, 1.33 |
| 10+ years since diagnosis | 282,808 | 1,516,843 | 1009 | 1.13 | 1.01, 1.26 | 1.10 | 0.98, 1.22 |
|  |  |  |  |  |  |  |  |
| Diseases of the GUS |  |  |  |  |  |  |  |
| Unexposed | 2,567,872 | 14,925,990 | 3877 | 1.00 | (ref) | 1.00 | (ref) |
| Exposed |  |  |  |  |  |  |  |
| 0-4 years since diagnosis | 307,032 | 941,496 | 340 | 1.08 | 0.89, 1.31 | 1.04 | 0.86, 1.26 |
| 5-9 years since diagnosis | 270,911 | 850,439 | 264 | 1.16 | 0.94, 1.45 | 1.15 | 0.92, 1.43 |
| 10+ years since diagnosis | 282,808 | 1,516,843 | 404 | 1.06 | 0.89, 1.28 | 1.06 | 0.88, 1.28 |
|  |  |  |  |  |  |  |  |
| Other causes |  |  |  |  |  |  |  |
| Unexposed | 2,567,872 | 14,925,990 | 30311 | 1.00 | (ref) | 1.00 | (ref) |
| Exposed |  |  |  |  |  |  |  |
| 0-4 years since diagnosis | 307,032 | 941,496 | 2235 | 0.89 | 0.83, 0.96 | 0.87 | 0.81, 0.94 |
| 5-9 years since diagnosis | 270,911 | 850,439 | 1837 | 1.06 | 0.98, 0.96 | 1.06 | 0.98, 1.15 |
| 10+ years since diagnosis | 282,808 | 1,516,843 | 2910 | 1.06 | 0.99, 1.13 | 1.06 | 0.99, 1.13 |
|  |  |  |  |  |  |  |  |

^1^Estimated hazard ratios from Cox regression with current age as underlying timescale, stratified by matched set (matched on age at cohort entry, gender, date at cohort entry and practice).

GUS: Genitourinary system

Unadjusted: No adjustment.

Adjusted: Adjusted for time since diagnosis (0-4, 5-9, 10-14, 15-19, 20+ years), current calendar period (2006-2010, 2011-2013, 2014-2016), Index of Multiple Deprivation at cohort entry and time-varying asthma.

# **Table E12**. Association between atopic eczema severity and all-cause and cause-specific mortality (cause-specific hazards) restricted to people with at least one consultation with their GP in the year prior to cohort entry. n = 2,478,735.

|  |  |  |  | HR & 99% CI^1^ | | | |
| --- | --- | --- | --- | --- | --- | --- | --- |
|  | n | P-Y at risk | Events | Unadjusted | | Adjusted | |
| **All-cause mortality** |  |  |  |  |  |  |  |
|  |  |  |  |  |  |  |  |
| All-cause mortality |  |  |  |  |  |  |  |
| Unexposed | 1,981,046 | 11,628,862 | 179,712 | 1.00 | (ref) | 1.00 | (ref) |
| Exposed – low | 362,686 | 1,880,509 | 23,790 | 0.98 | 0.96, 1.00 | 0.95 | 0.93, 0.98 |
| Exposed – moderate | 178,950 | 1,072,754 | 20,369 | 1.04 | 1.01, 1.06 | 1.01 | 0.99, 1.03 |
| Exposed – severe | 33,190 | 179,590 | 4,356 | 1.62 | 1.53, 1.71 | 1.55 | 1.47, 1.64 |
|  |  |  |  |  |  |  |  |
| **Cause-specific mortality** |  |  |  |  |  |  |  |
|  |  |  |  |  |  |  |  |
| Infections |  |  |  |  |  |  |  |
| Unexposed | 1,981,046 | 11,628,862 | 1,881 | 1.00 | (ref) | 1.00 | (ref) |
| Exposed – low | 362,686 | 1,880,509 | 240 | 0.92 | 0.74, 1.14 | 0.90 | 0.72, 1.12 |
| Exposed – moderate | 178,950 | 1,072,754 | 233 | 1.13 | 0.89, 1.43 | 1.11 | 0.87, 1.41 |
| Exposed – severe | 33,190 | 179,590 | 80 | 2.79 | 1.72, 4.51 | 2.60 | 1.60, 4.24 |
|  |  |  |  |  |  |  |  |
| Neoplasms |  |  |  |  |  |  |  |
| Unexposed | 1,981,046 | 11,628,862 | 50,023 | 1.00 | (ref) | 1.00 | (ref) |
| Exposed – low | 362,686 | 1,880,509 | 7,289 | 1.06 | 1.02, 1.10 | 1.05 | 1.01, 1.09 |
| Exposed – moderate | 178,950 | 1,072,754 | 5,490 | 1.01 | 0.96, 1.06 | 0.99 | 0.95, 1.04 |
| Exposed – severe | 33,190 | 179,590 | 977 | 1.26 | 1.13, 1.40 | 1.23 | 1.10, 1.37 |
|  |  |  |  |  |  |  |  |
| Circulatory disease |  |  |  |  |  |  |  |
| Unexposed | 1,981,046 | 11,628,862 | 62,022 | 1.00 | (ref) | 1.00 | (ref) |
| Exposed – low | 362,686 | 1,880,509 | 7,835 | 0.94 | 0.91, 0.98 | 0.93 | 0.89, 0.96 |
| Exposed – moderate | 178,950 | 1,072,754 | 7,239 | 1.07 | 1.02, 1.11 | 1.05 | 1.00, 1.09 |
| Exposed – severe | 33,190 | 179,590 | 1,398 | 1.51 | 1.36, 1.66 | 1.46 | 1.32, 1.61 |
|  |  |  |  |  |  |  |  |
| Respiratory disease |  |  |  |  |  |  |  |
| Unexposed | 1,981,046 | 11,628,862 | 26,127 | 1.00 | (ref) | 1.00 | (ref) |
| Exposed – low | 362,686 | 1,880,509 | 3,513 | 0.99 | 0.94, 1.05 | 0.91 | 0.86, 0.97 |
| Exposed – moderate | 178,950 | 1,072,754 | 3,157 | 1.11 | 1.04, 1.18 | 1.03 | 0.96, 1.10 |
| Exposed – severe | 33,190 | 179,590 | 857 | 2.25 | 1.96, 2.58 | 2.07 | 1.79, 2.40 |
|  |  |  |  |  |  |  |  |
| Digestive disease |  |  |  |  |  |  |  |
| Unexposed | 1,981,046 | 11,628,862 | 8,445 | 1.00 | (ref) | 1.00 | (ref) |
| Exposed – low | 362,686 | 1,880,509 | 1,109 | 0.95 | 0.86, 1.05 | 0.93 | 0.84, 1.03 |
| Exposed – moderate | 178,950 | 1,072,754 | 1,027 | 1.14 | 1.02, 1.27 | 1.11 | 0.99, 1.23 |
| Exposed – severe | 33,190 | 179,590 | 241 | 1.87 | 1.47, 2.37 | 1.81 | 1.43, 2.31 |
|  |  |  |  |  |  |  |  |
| Diseases of the GUS |  |  |  |  |  |  |  |
| Unexposed | 1,981,046 | 11,628,862 | 3,567 | 1.00 | (ref) | 1.00 | (ref) |
| Exposed – low | 362,686 | 1,880,509 | 442 | 0.93 | 0.79, 1.10 | 0.91 | 0.77, 1.08 |
| Exposed – moderate | 178,950 | 1,072,754 | 432 | 1.04 | 0.87, 1.25 | 1.02 | 0.85, 1.22 |
| Exposed – severe | 33,190 | 179,590 | 116 | 1.98 | 1.33, 2.93 | 1.91 | 1.29, 2.84 |
|  |  |  |  |  |  |  |  |
| Other causes |  |  |  |  |  |  |  |
| Unexposed | 1,981,046 | 11,628,862 | 27,647 | 1.00 | (ref) | 1.00 | (ref) |
| Exposed – low | 362,686 | 1,880,509 | 3,362 | 0.89 | 0.83, 0.94 | 0.88 | 0.83, 0.94 |
| Exposed – moderate | 178,950 | 1,072,754 | 2,791 | 0.92 | 0.85, 0.98 | 0.91 | 0.85, 0.97 |
| Exposed – severe | 33,190 | 179,590 | 687 | 1.86 | 1.61, 2.16 | 1.86 | 1.60, 2.15 |
|  |  |  |  |  |  |  |  |

^1^Estimated hazard ratios from Cox regression with current age as underlying timescale, stratified by matched set (matched on age at cohort entry, gender, date at cohort entry and practice).

GUS: Genitourinary system

Unadjusted: No adjustment.

Adjusted: Adjusted for current calendar period (1998-2001, 2002-2004, 2005-2007, 2008-2010, 2011-2013, 2014-2016), Index of Multiple Deprivation at cohort entry and time-varying asthma.

# **Table E13**. Association between atopic eczema activity and all-cause and cause-specific mortality (cause-specific hazards) restricted to people with at least one consultation with their GP in the year prior to cohort entry, by activity of atopic eczema (assessed during the first twelve months of follow-up, assuming active eczema for three months after a single health care contact), excluding the first 12 months of follow-up. n = 2,108,343.

|  |  |  |  | HR & 99% CI^1^ | | | |
| --- | --- | --- | --- | --- | --- | --- | --- |
|  | n | P-Y at risk | Events | Unadjusted | | Adjusted | |
| **All-cause mortality** |  |  |  |  |  |  |  |
|  |  |  |  |  |  |  |  |
| All-cause mortality |  |  |  |  |  |  |  |
| Unexposed | 1,677,147 | 9,805,871 | 152,673 | 1.00 | (ref) | 1.00 | (ref) |
| Exposed – never active | 150,359 | 942,201 | 12,208 | 0.97 | 0.94, 1.00 | 0.95 | 0.92, 0.98 |
| Exposed – <50% active | 197,526 | 1,236,168 | 15,497 | 0.95 | 0.92, 0.98 | 0.93 | 0.91, 0.96 |
| Exposed – 50+% active | 83,311 | 491,042 | 13,952 | 1.27 | 1.24, 1.31 | 1.22 | 1.18, 1.25 |
|  |  |  |  |  |  |  |  |
| **Cause-specific mortality** |  |  |  |  |  |  |  |
|  |  |  |  |  |  |  |  |
| Infections |  |  |  |  |  |  |  |
| Unexposed | 1,677,147 | 9,805,871 | 1,651 | 1.00 | (ref) | 1.00 | (ref) |
| Exposed – never active | 150,359 | 942,201 | 128 | 0.87 | 0.64, 1.18 | 0.86 | 0.63, 1.17 |
| Exposed – <50% active | 197,526 | 1,236,168 | 174 | 1.07 | 0.82, 1.39 | 1.04 | 0.80, 1.35 |
| Exposed – 50+% active | 83,311 | 491,042 | 185 | 1.48 | 1.12, 1.95 | 1.44 | 1.09, 1.90 |
|  |  |  |  |  |  |  |  |
| Neoplasms |  |  |  |  |  |  |  |
| Unexposed | 1,677,147 | 9,805,871 | 43,285 | 1.00 | (ref) | 1.00 | (ref) |
| Exposed – never active | 150,359 | 942,201 | 3,583 | 1.01 | 0.95, 1.07 | 1.00 | 0.94, 1.06 |
| Exposed – <50% active | 197,526 | 1,236,168 | 4,831 | 0.99 | 0.95, 1.04 | 0.98 | 0.94, 1.03 |
| Exposed – 50+% active | 83,311 | 491,042 | 3,277 | 1.12 | 1.05, 1.19 | 1.09 | 1.02, 1.15 |
|  |  |  |  |  |  |  |  |
| Circulatory disease |  |  |  |  |  |  |  |
| Unexposed | 1,677,147 | 9,805,871 | 52,371 | 1.00 | (ref) | 1.00 | (ref) |
| Exposed – never active | 150,359 | 942,201 | 4,261 | 1.00 | 0.95, 1.05 | 0.98 | 0.93, 1.04 |
| Exposed – <50% active | 197,526 | 1,236,168 | 5,165 | 0.93 | 0.89, 0.98 | 0.92 | 0.88, 0.96 |
| Exposed – 50+% active | 83,311 | 491,042 | 4,766 | 1.25 | 1.18, 1.31 | 1.21 | 1.15, 1.28 |
|  |  |  |  |  |  |  |  |
| Respiratory disease |  |  |  |  |  |  |  |
| Unexposed | 1,677,147 | 9,805,871 | 22,162 | 1.00 | (ref) | 1.00 | (ref) |
| Exposed – never active | 150,359 | 942,201 | 1,721 | 0.92 | 0.84, 1.01 | 0.87 | 0.79, 0.95 |
| Exposed – <50% active | 197,526 | 1,236,168 | 2,244 | 0.98 | 0.91, 1.06 | 0.93 | 0.87, 1.01 |
| Exposed – 50+% active | 83,311 | 491,042 | 2,603 | 1.60 | 1.48, 1.72 | 1.40 | 1.29, 1.51 |
|  |  |  |  |  |  |  |  |
| Digestive disease |  |  |  |  |  |  |  |
| Unexposed | 1,677,147 | 9,805,871 | 7,314 | 1.00 | (ref) | 1.00 | (ref) |
| Exposed – never active | 150,359 | 942,201 | 617 | 0.98 | 0.85, 1.13 | 0.96 | 0.83, 1.11 |
| Exposed – <50% active | 197,526 | 1,236,168 | 772 | 0.99 | 0.88, 1.12 | 0.98 | 0.87, 1.10 |
| Exposed – 50+% active | 83,311 | 491,042 | 674 | 1.33 | 1.16, 1.53 | 1.29 | 1.13, 1.48 |
|  |  |  |  |  |  |  |  |
| Diseases of the GUS |  |  |  |  |  |  |  |
| Unexposed | 1,677,147 | 9,805,871 | 3,079 | 1.00 | (ref) | 1.00 | (ref) |
| Exposed – never active | 150,359 | 942,201 | 244 | 0.97 | 0.76, 1.24 | 0.96 | 0.75, 1.23 |
| Exposed – <50% active | 197,526 | 1,236,168 | 283 | 0.81 | 0.65, 1.00 | 0.80 | 0.64, 0.99 |
| Exposed – 50+% active | 83,311 | 491,042 | 334 | 1.45 | 1.17, 1.78 | 1.40 | 1.13, 1.73 |
|  |  |  |  |  |  |  |  |
| Other causes |  |  |  |  |  |  |  |
| Unexposed | 1,677,147 | 9,805,871 | 22,811 | 1.00 | (ref) | 1.00 | (ref) |
| Exposed – never active | 150,359 | 942,201 | 1,654 | 0.85 | 0.77, 0.93 | 0.84 | 0.77, 0.92 |
| Exposed – <50% active | 197,526 | 1,236,168 | 2,028 | 0.86 | 0.79, 0.92 | 0.85 | 0.79, 0.92 |
| Exposed – 50+% active | 83,311 | 491,042 | 2,113 | 1.27 | 1.16, 1.37 | 1.25 | 1.15, 1.36 |
|  |  |  |  |  |  |  |  |

^1^Estimated hazard ratios from Cox regression with current age as underlying timescale, stratified by matched set (matched on age at cohort entry, gender, date at cohort entry and practice).

GUS: Genitourinary system

Unadjusted: No adjustment.

Adjusted: Adjusted for current calendar period (1998-2001, 2002-2004, 2005-2007, 2008-2010, 2011-2013, 2014-2016), Index of Multiple Deprivation at cohort entry and time-varying asthma.

# **Table E14**. Association between atopic eczema activity and all-cause and cause-specific mortality (cause-specific hazards), by activity of atopic eczema (second activity definition: assessed during the first twelve months of follow-up, assuming active eczema for three months after a single health care contact), without excluding the first 12 months of follow-up. n = 3,094,608.

|  |  |  |  | HR & 99% CI^1^ | | | |
| --- | --- | --- | --- | --- | --- | --- | --- |
|  | n | P-Y at risk | Events | Unadjusted | | Adjusted | |
| **All-cause mortality** |  |  |  |  |  |  |  |
|  |  |  |  |  |  |  |  |
| All-cause mortality |  |  |  |  |  |  |  |
| Unexposed | 2,567,872 | 14,925,990 | 199,645 | 1.00 | (ref) | 1.00 | (ref) |
| Exposed – never active | 200,783 | 1,230,637 | 14,444 | 0.99 | 0.96, 1.01 | 0.96 | 0.94, 0.99 |
| Exposed – <50% active | 215,991 | 1,482,505 | 16,808 | 0.91 | 0.89, 0.93 | 0.89 | 0.86, 0.91 |
| Exposed – 50+% active | 109,962 | 595,636 | 18,262 | 1.42 | 1.39, 1.46 | 1.35 | 1.32, 1.39 |
|  |  |  |  |  |  |  |  |
| **Cause-specific mortality** |  |  |  |  |  |  |  |
|  |  |  |  |  |  |  |  |
| Infections |  |  |  |  |  |  |  |
| Unexposed | 2,567,872 | 14,925,990 | 2,040 | 1.00 | (ref) | 1.00 | (ref) |
| Exposed – never active | 200,783 | 1,230,637 | 159 | 0.96 | 0.73, 1.25 | 0.94 | 0.72, 1.24 |
| Exposed – <50% active | 215,991 | 1,482,505 | 188 | 1.07 | 0.83, 1.36 | 1.03 | 0.80, 1.32 |
| Exposed – 50+% active | 109,962 | 595,636 | 216 | 1.58 | 1.23, 2.03 | 1.52 | 1.18, 1.96 |
|  |  |  |  |  |  |  |  |
| Neoplasms |  |  |  |  |  |  |  |
| Unexposed | 2,567,872 | 14,925,990 | 56,873 | 1.00 | (ref) | 1.00 | (ref) |
| Exposed – never active | 200,783 | 1,230,637 | 4,194 | 1.01 | 0.96, 1.06 | 1.00 | 0.95, 1.05 |
| Exposed – <50% active | 215,991 | 1,482,505 | 5,229 | 0.95 | 0.91, 0.99 | 0.94 | 0.90, 0.98 |
| Exposed – 50+% active | 109,962 | 595,636 | 4,629 | 1.37 | 1.30, 1.44 | 1.33 | 1.26, 1.40 |
|  |  |  |  |  |  |  |  |
| Circulatory disease |  |  |  |  |  |  |  |
| Unexposed | 2,567,872 | 14,925,990 | 68,669 | 1.00 | (ref) | 1.00 | (ref) |
| Exposed – never active | 200,783 | 1,230,637 | 5,082 | 1.03 | 0.98, 1.08 | 1.01 | 0.96, 1.06 |
| Exposed – <50% active | 215,991 | 1,482,505 | 5,606 | 0.88 | 0.84, 0.92 | 0.87 | 0.83, 0.91 |
| Exposed – 50+% active | 109,962 | 595,636 | 6,100 | 1.34 | 1.28, 1.40 | 1.30 | 1.24, 1.36 |
|  |  |  |  |  |  |  |  |
| Respiratory disease |  |  |  |  |  |  |  |
| Unexposed | 2,567,872 | 14,925,990 | 28,383 | 1.00 | (ref) | 1.00 | (ref) |
| Exposed – never active | 200,783 | 1,230,637 | 2,013 | 0.94 | 0.87, 1.02 | 0.88 | 0.81, 0.95 |
| Exposed – <50% active | 215,991 | 1,482,505 | 2,429 | 0.95 | 0.89, 1.02 | 0.89 | 0.83, 0.95 |
| Exposed – 50+% active | 109,962 | 595,636 | 3,228 | 1.68 | 1.58, 1.79 | 1.46 | 1.36, 1.56 |
|  |  |  |  |  |  |  |  |
| Digestive disease |  |  |  |  |  |  |  |
| Unexposed | 2,567,872 | 14,925,990 | 9,492 | 1.00 | (ref) | 1.00 | (ref) |
| Exposed – never active | 200,783 | 1,230,637 | 741 | 1.03 | 0.91, 1.17 | 1.01 | 0.89, 1.15 |
| Exposed – <50% active | 215,991 | 1,482,505 | 842 | 0.97 | 0.87, 1.09 | 0.95 | 0.85, 1.06 |
| Exposed – 50+% active | 109,962 | 595,636 | 868 | 1.52 | 1.35, 1.71 | 1.46 | 1.29, 1.64 |
|  |  |  |  |  |  |  |  |
| Diseases of the GUS |  |  |  |  |  |  |  |
| Unexposed | 2,567,872 | 14,925,990 | 3,877 | 1.00 | (ref) | 1.00 | (ref) |
| Exposed – never active | 200,783 | 1,230,637 | 277 | 1.01 | 0.81, 1.25 | 0.99 | 0.80, 1.24 |
| Exposed – <50% active | 215,991 | 1,482,505 | 306 | 0.80 | 0.66, 0.98 | 0.79 | 0.65, 0.97 |
| Exposed – 50+% active | 109,962 | 595,636 | 425 | 1.57 | 1.31, 1.88 | 1.52 | 1.27, 1.82 |
|  |  |  |  |  |  |  |  |
| Other causes |  |  |  |  |  |  |  |
| Unexposed | 2,567,872 | 14,925,990 | 30,311 | 1.00 | (ref) | 1.00 | (ref) |
| Exposed – never active | 200,783 | 1,230,637 | 1,978 | 0.86 | 0.80, 0.93 | 0.86 | 0.79, 0.93 |
| Exposed – <50% active | 215,991 | 1,482,505 | 2,208 | 0.81 | 0.76, 0.87 | 0.81 | 0.75, 0.87 |
| Exposed – 50+% active | 109,962 | 595,636 | 2,796 | 1.41 | 1.32, 1.52 | 1.39 | 1.30, 1.49 |
|  |  |  |  |  |  |  |  |

^1^Estimated hazard ratios from Cox regression with current age as underlying timescale, stratified by matched set (matched on age at cohort entry, gender, date at cohort entry and practice).

GUS: Genitourinary system

Unadjusted: No adjustment.

Adjusted: Adjusted for current calendar period (1998-2001, 2002-2004, 2005-2007, 2008-2010, 2011-2013, 2014-2016), Index of Multiple Deprivation at cohort entry and time-varying asthma.

# **Table E15**. Association between atopic eczema activity and all-cause and cause-specific mortality (cause-specific hazards), by activity of atopic eczema (pre-specified activity definition: assessed across all of follow-up, assuming active eczema for 12 months after two health care contacts). n = 3,094,608.

|  |  |  |  | HR & 99% CI^1^ | | | |
| --- | --- | --- | --- | --- | --- | --- | --- |
|  | n | P-Y at risk | Events | Unadjusted | | Adjusted | |
| **All-cause mortality** |  |  |  |  |  |  |  |
|  |  |  |  |  |  |  |  |
| All-cause mortality |  |  |  |  |  |  |  |
| Unexposed | 2,567,872 | 14,925,990 | 199,645 | 1.00 | (ref) | 1.00 | (ref) |
| Exposed – never active | 193,505 | 977,278 | 9,467 | 1.19 | 1.15, 1.23 | 1.17 | 1.13, 1.21 |
| Exposed – <50% active | 172,868 | 1,518,688 | 14,241 | 0.72 | 0.71, 0.74 | 0.71 | 0.69, 0.73 |
| Exposed – 50+% active | 160,363 | 812,812 | 25,806 | 1.40 | 1.37, 1.43 | 1.33 | 1.30, 1.36 |
|  |  |  |  |  |  |  |  |
| **Cause-specific mortality** |  |  |  |  |  |  |  |
|  |  |  |  |  |  |  |  |
| Infections |  |  |  |  |  |  |  |
| Unexposed | 2,567,872 | 14,925,990 | 2,040 | 1.00 | (ref) | 1.00 | (ref) |
| Exposed – never active | 193,505 | 977,278 | 94 | 1.11 | 0.79, 1.57 | 1.09 | 0.77, 1.54 |
| Exposed – <50% active | 172,868 | 1,518,688 | 160 | 0.80 | 0.62, 1.04 | 0.79 | 0.61, 1.03 |
| Exposed – 50+% active | 160,363 | 812,812 | 309 | 1.60 | 1.30, 1.97 | 1.53 | 1.23, 1.89 |
|  |  |  |  |  |  |  |  |
| Neoplasms |  |  |  |  |  |  |  |
| Unexposed | 2,567,872 | 14,925,990 | 56,873 | 1.00 | (ref) | 1.00 | (ref) |
| Exposed – never active | 193,505 | 977,278 | 2,644 | 1.13 | 1.06, 1.21 | 1.13 | 1.06, 1.20 |
| Exposed – <50% active | 172,868 | 1,518,688 | 4,893 | 0.84 | 0.80, 0.88 | 0.83 | 0.79, 0.87 |
| Exposed – 50+% active | 160,363 | 812,812 | 6,515 | 1.34 | 1.28, 1.40 | 1.30 | 1.25, 1.36 |
|  |  |  |  |  |  |  |  |
| Circulatory disease |  |  |  |  |  |  |  |
| Unexposed | 2,567,872 | 14,925,990 | 68,669 | 1.00 | (ref) | 1.00 | (ref) |
| Exposed – never active | 193,505 | 977,278 | 3,539 | 1.38 | 1.30, 1.46 | 1.36 | 1.28, 1.44 |
| Exposed – <50% active | 172,868 | 1,518,688 | 4,548 | 0.67 | 0.64, 0.70 | 0.67 | 0.63, 0.70 |
| Exposed – 50+% active | 160,363 | 812,812 | 8,701 | 1.32 | 1.28, 1.37 | 1.28 | 1.23, 1.33 |
|  |  |  |  |  |  |  |  |
| Respiratory disease |  |  |  |  |  |  |  |
| Unexposed | 2,567,872 | 14,925,990 | 28,383 | 1.00 | (ref) | 1.00 | (ref) |
| Exposed – never active | 193,505 | 977,278 | 1,192 | 1.02 | 0.92, 1.13 | 0.97 | 0.87, 1.07 |
| Exposed – <50% active | 172,868 | 1,518,688 | 1,857 | 0.68 | 0.63, 0.74 | 0.66 | 0.61, 0.71 |
| Exposed – 50+% active | 160,363 | 812,812 | 4,621 | 1.68 | 1.60, 1.78 | 1.45 | 1.37, 1.54 |
|  |  |  |  |  |  |  |  |
| Digestive disease |  |  |  |  |  |  |  |
| Unexposed | 2,567,872 | 14,925,990 | 9,492 | 1.00 | (ref) | 1.00 | (ref) |
| Exposed – never active | 193,505 | 977,278 | 492 | 1.32 | 1.13, 1.54 | 1.30 | 1.12, 1.52 |
| Exposed – <50% active | 172,868 | 1,518,688 | 722 | 0.77 | 0.69, 0.87 | 0.76 | 0.67, 0.86 |
| Exposed – 50+% active | 160,363 | 812,812 | 1,237 | 1.47 | 1.33, 1.62 | 1.40 | 1.27, 1.55 |
|  |  |  |  |  |  |  |  |
| Diseases of the GUS |  |  |  |  |  |  |  |
| Unexposed | 2,567,872 | 14,925,990 | 3,877 | 1.00 | (ref) | 1.00 | (ref) |
| Exposed – never active | 193,505 | 977,278 | 177 | 1.37 | 1.04, 1.80 | 1.36 | 1.03, 1.79 |
| Exposed – <50% active | 172,868 | 1,518,688 | 250 | 0.60 | 0.48, 0.74 | 0.60 | 0.48, 0.74 |
| Exposed – 50+% active | 160,363 | 812,812 | 581 | 1.49 | 1.28, 1.74 | 1.44 | 1.23, 1.69 |
|  |  |  |  |  |  |  |  |
| Other causes |  |  |  |  |  |  |  |
| Unexposed | 2,567,872 | 14,925,990 | 30,311 | 1.00 | (ref) | 1.00 | (ref) |
| Exposed – never active | 193,505 | 977,278 | 1,329 | 0.99 | 0.90, 1.09 | 0.99 | 0.90, 1.09 |
| Exposed – <50% active | 172,868 | 1,518,688 | 1,811 | 0.63 | 0.58, 0.68 | 0.63 | 0.58, 0.68 |
| Exposed – 50+% active | 160,363 | 812,812 | 3,842 | 1.38 | 1.30, 1.46 | 1.36 | 1.28, 1.44 |
|  |  |  |  |  |  |  |  |

^1^Estimated hazard ratios from Cox regression with current age as underlying timescale, stratified by matched set (matched on age at cohort entry, gender, date at cohort entry and practice).

GUS: Genitourinary system

Unadjusted: No adjustment.

Adjusted: Adjusted for current calendar period (1998-2001, 2002-2004, 2005-2007, 2008-2010, 2011-2013, 2014-2016), Index of Multiple Deprivation at cohort entry and time-varying asthma.

# **Table E16**. Association between atopic eczema activity and all-cause and cause-specific mortality (cause-specific hazards), by activity of atopic eczema (pre-specified activity definition: assessed across all of follow-up, assuming active eczema for 12 months after two health care contacts), restricted to patients with at least 5 years of follow-up. Fitted to individuals from valid matched sets^1^. n = 1,159,330.

|  |  |  |  | HR & 99% CI^2^ | | | |
| --- | --- | --- | --- | --- | --- | --- | --- |
|  | n | P-Y at risk | Events | Unadjusted | | Adjusted | |
| **All-cause mortality** |  |  |  |  |  |  |  |
|  |  |  |  |  |  |  |  |
| All-cause mortality |  |  |  |  |  |  |  |
| Unexposed | 900,432 | 9,317,543 | 63,247 | 1.00 | (ref) | 1.00 | (ref) |
| Exposed – never active | 74,154 | 728,858 | 3,210 | 1.00 | 0.94, 1.06 | 1.02 | 0.96, 1.09 |
| Exposed – <50% active | 124,730 | 1,353,661 | 9,570 | 0.93 | 0.90, 0.97 | 0.93 | 0.90, 0.97 |
| Exposed – 50+% active | 60,014 | 613,729 | 9,620 | 1.30 | 1.25, 1.35 | 1.25 | 1.20, 1.29 |
|  |  |  |  |  |  |  |  |
| **Cause-specific mortality** |  |  |  |  |  |  |  |
|  |  |  |  |  |  |  |  |
| Infections |  |  |  |  |  |  |  |
| Unexposed | 900,432 | 9,317,543 | 736 | 1.00 | (ref) | 1.00 | (ref) |
| Exposed – never active | 74,154 | 728,858 | 34 | 1.01 | 0.57, 1.79 | 1.08 | 0.60, 1.96 |
| Exposed – <50% active | 124,730 | 1,353,661 | 115 | 0.82 | 0.60, 1.13 | 0.83 | 0.60, 1.14 |
| Exposed – 50+% active | 60,014 | 613,729 | 142 | 1.68 | 1.23, 2.30 | 1.66 | 1.20, 2.29 |
|  |  |  |  |  |  |  |  |
| Neoplasms |  |  |  |  |  |  |  |
| Unexposed | 900,432 | 9,317,543 | 20,820 | 1.00 | (ref) | 1.00 | (ref) |
| Exposed – never active | 74,154 | 728,858 | 907 | 0.86 | 0.77, 0.95 | 0.89 | 0.80, 0.99 |
| Exposed – <50% active | 124,730 | 1,353,661 | 3,243 | 1.00 | 0.95, 1.06 | 1.02 | 0.96, 1.08 |
| Exposed – 50+% active | 60,014 | 613,729 | 2,355 | 1.08 | 1.01, 1.16 | 1.06 | 0.99, 1.14 |
|  |  |  |  |  |  |  |  |
| Circulatory disease |  |  |  |  |  |  |  |
| Unexposed | 900,432 | 9,317,543 | 20,496 | 1.00 | (ref) | 1.00 | (ref) |
| Exposed – never active | 74,154 | 728,858 | 1,103 | 1.10 | 1.00, 1.23 | 1.13 | 1.02, 1.26 |
| Exposed – <50% active | 124,730 | 1,353,661 | 3,013 | 0.89 | 0.83, 0.94 | 0.89 | 0.84, 0.95 |
| Exposed – 50+% active | 60,014 | 613,729 | 3,237 | 1.26 | 1.19, 1.34 | 1.24 | 1.17, 1.32 |
|  |  |  |  |  |  |  |  |
| Respiratory disease |  |  |  |  |  |  |  |
| Unexposed | 900,432 | 9,317,543 | 8,549 | 1.00 | (ref) | 1.00 | (ref) |
| Exposed – never active | 74,154 | 728,858 | 424 | 0.91 | 0.77, 1.09 | 0.93 | 0.77, 1.11 |
| Exposed – <50% active | 124,730 | 1,353,661 | 1,315 | 0.94 | 0.86, 1.04 | 0.93 | 0.84, 1.03 |
| Exposed – 50+% active | 60,014 | 613,729 | 1,853 | 1.74 | 1.60, 1.91 | 1.49 | 1.35, 1.63 |
|  |  |  |  |  |  |  |  |
| Digestive disease |  |  |  |  |  |  |  |
| Unexposed | 900,432 | 9,317,543 | 3,207 | 1.00 | (ref) | 1.00 | (ref) |
| Exposed – never active | 74,154 | 728,858 | 191 | 1.21 | 0.94, 1.55 | 1.21 | 0.94, 1.56 |
| Exposed – <50% active | 124,730 | 1,353,661 | 483 | 0.96 | 0.83, 1.12 | 0.95 | 0.82, 1.11 |
| Exposed – 50+% active | 60,014 | 613,729 | 462 | 1.30 | 1.11, 1.53 | 1.26 | 1.07, 1.48 |
|  |  |  |  |  |  |  |  |
| Diseases of the GUS |  |  |  |  |  |  |  |
| Unexposed | 900,432 | 9,317,543 | 1,181 | 1.00 | (ref) | 1.00 | (ref) |
| Exposed – never active | 74,154 | 728,858 | 69 | 1.28 | 0.82, 2.00 | 1.29 | 0.82, 2.03 |
| Exposed – <50% active | 124,730 | 1,353,661 | 173 | 0.76 | 0.58, 1.00 | 0.78 | 0.59, 1.02 |
| Exposed – 50+% active | 60,014 | 613,729 | 245 | 1.66 | 1.29, 2.13 | 1.60 | 1.24, 2.07 |
|  |  |  |  |  |  |  |  |
| Other causes |  |  |  |  |  |  |  |
| Unexposed | 900,432 | 9,317,543 | 8,258 | 1.00 | (ref) | 1.00 | (ref) |
| Exposed – never active | 74,154 | 728,858 | 482 | 1.10 | 0.94, 1.29 | 1.12 | 0.96, 1.32 |
| Exposed – <50% active | 124,730 | 1,353,661 | 1,228 | 0.87 | 0.78, 0.96 | 0.88 | 0.80, 0.97 |
| Exposed – 50+% active | 60,014 | 613,729 | 1,326 | 1.38 | 1.24, 1.52 | 1.37 | 1.23, 1.52 |
|  |  |  |  |  |  |  |  |

^1^Matched sets including one exposed person and at least one unexposed person.

^2^Estimated hazard ratios from Cox regression with current age as underlying timescale, stratified by matched set (matched on age at cohort entry, gender, date at cohort entry and practice).

^3^Exploratory analysis additionally adjusting for potential mediators.

GUS: Genitourinary system

Unadjusted: No adjustment.

Adjusted: Adjusted for current calendar period (1998-2001, 2002-2004, 2005-2007, 2008-2010, 2011-2013, 2014-2016), Index of Multiple Deprivation at cohort entry and time-varying asthma.

# **Table E17**. Covariate summary statistics. Number (%) unless otherwise stated.

|  | Overall sample | | |  | Complete data on analysis variables | | |  | From valid matched set^1^ (mediation analysis sample) | | |
| --- | --- | --- | --- | --- | --- | --- | --- | --- | --- | --- | --- |
|  | Without  atopic eczema  n = 2,567,872  (83.0%) | With atopic  eczema  n = 526,736  (17.0%) | Total  n = 3,094,608 |  | Without  atopic eczema  n = 2,063,597  (82.3%) | With atopic  eczema  n = 445,152  (17.7%) | Total  n = 2,508,749 |  | Without  atopic eczema  n = 1,838,627  (80.7%) | With atopic  eczema  n = 440,317  (19.3%) | Total  n = 2,278,944 |
| Follow-up (years) |  |  |  |  |  |  |  |  |  |  |  |
| Median (1^st^ and  3^rd^ quartiles) | 4.4 (1.7, 8.9) | 5.0 (2.0, 9.6) | 4.5 (1.7, 9.0) |  | 4.9 (1.9, 9.5) | 5.5 (2.3, 10.3) | 5.0 (2.0, 9.7) |  | 5.0 (2.0, 9.7) | 5.5 (2.3, 10.3) | 5.1 (2.1, 9.80 |
|  |  |  |  |  |  |  |  |  |  |  |  |
| **At entry to cohort** |  |  |  |  |  |  |  |  |  |  |  |
| Gender |  |  |  |  |  |  |  |  |  |  |  |
| Male | 1,079,198 (42.0) | 218,702 (41.5) | 1,297,900 (41.9) |  | 792,228 (38.4) | 170,608 (38.3) | 962,836 (38.4) |  | 677,591 (36.9) | 167,748 (38.1) | 845,339 (37.1) |
| Female | 1,488,674 (58.0) | 308,034 (58.5) | 1,796,708 (58.1) |  | 1,271,369 (61.6) | 274,544 (61.7) | 1,545,913 (61.6) |  | 1,161,036 (63.1) | 272,569 (61.9) | 1,433,605 (62.9) |
|  |  |  |  |  |  |  |  |  |  |  |  |
| Age (years) |  |  |  |  |  |  |  |  |  |  |  |
| Median (1st and  3^rd^ quartiles) | 41.7 (27.0, 60.8) | 42.3 (25.7, 62.3) | 41.8 (26.7, 61.1) |  | 44.9 (30.3, 62.1) | 45.5 (30.0, 63.6) | 45.0 (30.2, 62.5) |  | 46.6 (32.1, 62.7) | 45.7 (30.3, 63.6) | 46.5 (31.8, 62.9) |
| 18-19 | 317,332 (12.4) | 81,760 (15.5) | 399,092 (12.9) |  | 142,933 (6.9) | 40,912 (9.2) | 183,845 (7.3) |  | 91,676 (5.0) | 38,455 (8.7) | 130,131 (5.7) |
| 20-29 | 471,212 (18.4) | 84,511 (16.0) | 555,723 (18.0) |  | 363,495 (17.6) | 70,307 (15.8) | 433,802 (17.3) |  | 295,171 (16.1) | 69,635 (15.8) | 364,806 (16.0) |
| 30-39 | 429,028 (16.7) | 80,315 (15.2) | 509,343 (16.5) |  | 366,999 (17.8) | 73,713 (16.6) | 440,712 (17.6) |  | 333,430 (18.1) | 73,541 (16.7) | 406,971 (17.9) |
| 40-49 | 351,880 (13.7) | 69,694 (13.2) | 421,574 (13.6) |  | 311,698 (15.1) | 65,436 (14.7) | 377,134 (15.0) |  | 294,373 (16.0) | 65,371 (14.8) | 359,744 (15.8) |
| 50-59 | 328,941 (12.8) | 63,942 (12.1) | 392,883 (12.7) |  | 298,242 (14.5) | 60,977 (13.7) | 359,219 (14.3) |  | 285,382 (15.5) | 60,888 (13.8) | 346,270 (15.2) |
| 60-69 | 303,674 (11.8) | 61,896 (11.8) | 365,570 (11.8) |  | 279,626 (13.6) | 59,322 (13.3) | 338,948 (13.5) |  | 268,197 (14.6) | 59,184 (13.4) | 327,381 (14.4) |
| 70-79 | 227,150 (8.8) | 52,037 (9.9) | 279,187 (9.0) |  | 200,364 (9.7) | 48,239 (10.8) | 248,603 (9.9) |  | 185,709 (10.1) | 47,862 (10.9) | 233,571 (10.2) |
| 80+ | 138,655 (5.4) | 32,581 (6.2) | 171,236 (5.5) |  | 100,240 (4.9) | 26,246 (5.9) | 126,486 (5.0) |  | 84,689 (4.6) | 25,381 (5.8) | 110,070 (4.8) |
|  |  |  |  |  |  |  |  |  |  |  |  |
| IMD |  |  |  |  |  |  |  |  |  |  |  |
| 1 (Least deprived) | 611,696 (23.8) | 126,788 (24.1) | 738,484 (23.9) |  | 492,258 (23.9) | 106,712 (24.0) | 598,970 (23.9) |  | 440,206 (23.9) | 105,492 (24.0) | 545,698 (23.9) |
| 2 | 589,085 (22.9) | 120,936 (23.0) | 710,021 (22.9) |  | 474,772 (23.0) | 102,306 (23.0) | 577,078 (23.0) |  | 423,456 (23.0) | 101,188 (23.0) | 524,644 (23.0) |
| 3 | 508,244 (19.8) | 103,632 (19.7) | 611,876 (19.8) |  | 407,877 (19.8) | 87,235 (19.6) | 495,112 (19.7) |  | 362,701 (19.7) | 86,239 (19.6) | 448,940 (19.7) |
| 4 | 488,876 (19.0) | 100,415 (19.1) | 589,291 (19.0) |  | 393,946 (19.1) | 85,209 (19.1) | 479,155 (19.1) |  | 350,529 (19.1) | 84,365 (19.2) | 434,894 (19.1) |
| 5 (Most deprived) | 369,971 (14.4) | 74,965 (14.2) | 444,936 (14.4) |  | 294,744 (14.3) | 63,690 (14.3) | 358,434 (14.3) |  | 261,735 (14.2) | 63,033 (14.3) | 324,768 (14.3) |
|  |  |  |  |  |  |  |  |  |  |  |  |
| BMI |  |  |  |  |  |  |  |  |  |  |  |
| Underweight | 65,917 (3.2) | 13,442 (3.0) | 79,359 (3.1) |  | 65,109 (3.2) | 13,324 (3.0) | 78,433 (3.1) |  | 53,392 (2.9) | 12,950 (2.9) | 66,342 (2.9) |
| Normal weight | 947,898 (45.7) | 196,737 (44.1) | 1,144,635 (45.4) |  | 942,545 (45.7) | 196,121 (44.1) | 1,138,666 (45.4) |  | 827,011 (45.0) | 193,656 (44.0) | 1,020,667 (44.8) |
| Overweight | 667,042 (32.2) | 143,901 (32.2) | 810,943 (32.2) |  | 664,183 (32.2) | 143,495 (32.2) | 807,678 (32.2) |  | 601,001 (32.7) | 142,283 (32.3) | 743,284 (32.6) |
| Obese | 393,485 (19.0) | 92,520 (20.7) | 486,005 (19.3) |  | 391,760 (19.0) | 92,212 (20.7) | 483,972 (19.3) |  | 357,223 (19.4) | 91,428 (20.8) | 448,651 (19.7) |
|  |  |  |  |  |  |  |  |  |  |  |  |
| Smoking |  |  |  |  |  |  |  |  |  |  |  |
| Non-smoker | 1,293,562 (53.5) | 266,116 (51.9) | 1,559,678 (53.2) |  | 1,077,485 (52.2) | 222,934 (50.1) | 1,300,419 (51.8) |  | 952,392 (51.8) | 220,050 (50.0) | 1,172,442 (51.4) |
| Current/ex-smoker | 1,125,203 (46.5) | 246,748 (48.1) | 1,371,951 (46.8) |  | 986,112 (47.8) | 222,218 (49.9) | 1,208,330 (48.2) |  | 886,235 (48.2) | 220,267 (50.0) | 1,106,502 (48.6) |
|  |  |  |  |  |  |  |  |  |  |  |  |
| Diabetes | 127,484 (5.0) | 32,387 (6.1) | 159,871 (5.2) |  | 120,274 (5.8) | 31,039 (7.0) | 151,313 (6.0) |  | 110,491 (6.0) | 30,660 (7.0) | 141,151 (6.2) |
| Depression | 53,619 (2.1) | 15,382 (2.9) | 69,001 (2.2) |  | 46,917 (2.3) | 14,080 (3.2) | 60,997 (2.4) |  | 42,893 (2.3) | 13,973 (3.2) | 56,866 (2.5) |
| Anxiety | 18,577 (0.7) | 5,941 (1.1) | 24,518 (0.8) |  | 16,612 (0.8) | 5,481 (1.2) | 22,093 (0.9) |  | 15,203 (0.8) | 5,451 (1.2) | 20,654 (0.9) |
| Asthma | 318,263 (12.4) | 125,815 (23.9) | 444,078 (14.4) |  | 263,265 (12.8) | 104,794 (23.5) | 368,059 (14.7) |  | 228,203 (12.4) | 102,909 (23.4) | 331,112 (14.5) |
| Severe alcohol use | 57,215 (2.2) | 14,351 (2.7) | 71,566 (2.3) |  | 51,037 (2.5) | 13,234 (3.0) | 64,271 (2.6) |  | 46,618 (2.5) | 13,149 (3.0) | 59,767 (2.6) |
|  |  |  |  |  |  |  |  |  |  |  |  |
| **By exit from cohort** |  |  |  |  |  |  |  |  |  |  |  |
| Diabetes | 233,623 (9.1) | 59,922 (11.4) | 293,545 (9.5) |  | 221,935 (10.8) | 57,669 (13.0) | 279,604 (11.1) |  | 204,524 (11.1) | 57,140 (13.0) | 261,664 (11.5) |
| Depression | 107,424 (4.2) | 31,018 (5.9) | 138,442 (4.5) |  | 95,790 (4.6) | 28,665 (6.4) | 124,455 (5.0) |  | 87,202 (4.7) | 28,440 (6.5) | 115,642 (5.1) |
| Anxiety | 33,302 (1.3) | 10,067 (1.9) | 43,369 (1.4) |  | 30,073 (1.5) | 9,291 (2.1) | 39,364 (1.6) |  | 27,296 (1.5) | 9,232 (2.1) | 36,528 (1.6) |
| Asthma | 385,700 (15.0) | 146,179 (27.8) | 531,879 (17.2) |  | 326,377 (15.8) | 124,040 (27.9) | 450,417 (18.0) |  | 285,677 (15.5) | 122,039 (27.7) | 407,716 (17.9) |
| Severe alcohol use | 86,306 (3.4) | 21,794 (4.1) | 108,100 (3.5) |  | 77,427 (3.8) | 20,117 (4.5) | 97,544 (3.9) |  | 70,543 (3.8) | 19,979 (4.5) | 90,522 (4.0) |
|  |  |  |  |  |  |  |  |  |  |  |  |
| **Death from** |  |  |  |  |  |  |  |  |  |  |  |
| Infections | 2,040 (0.1) | 563 (0.1) | 2,603 (0.1) |  | 1,679 (0.1) | 486 (0.1) | 2,165 (0.1) |  | 1,493 (0.1) | 480 (0.1) | 1,973 (0.1) |
| Neoplasms | 56,873 (2.2) | 14,052 (2.7) | 70,925 (2.3) |  | 48,334 (2.3) | 12,493 (2.8) | 60,827 (2.4) |  | 44,567 (2.4) | 12,404 (2.8) | 56,971 (2.5) |
| Circulatory disease | 68,669 (2.7) | 16,788 (3.2) | 85,457 (2.8) |  | 53,430 (2.6) | 14,083 (3.2) | 67,513 (2.7) |  | 47,254 (2.6) | 13,887 (3.2) | 61,141 (2.7) |
| Respiratory disease | 28,383 (1.1) | 7,670 (1.5) | 36,053 (1.2) |  | 21,633 (1.0) | 6,347 (1.4) | 27,980 (1.1) |  | 19,062 (1.0) | 6,243 (1.4) | 25,305 (1.1) |
| Digestive disease | 9,492 (0.4) | 2,451 (0.5) | 11,943 (0.4) |  | 7,556 (0.4) | 2,105 (0.5) | 9,661 (0.4) |  | 6,788 (0.4) | 2,087 (0.5) | 8,875 (0.4) |
| Diseases of the GUS | 3,877 (0.2) | 1,008 (0.2) | 4,885 (0.2) |  | 2,934 (0.1) | 835 (0.2) | 3,769 (0.2) |  | 2,535 (0.1) | 818 (0.2) | 3,353 (0.1) |
| Other causes | 30,311 (1.2) | 6,982 (1.3) | 37,293 (1.2) |  | 20,957 (1.0) | 5,410 (1.2) | 26,367 (1.1) |  | 18,282 (1.0) | 5,310 (1.2) | 23,592 (1.0) |
| Any cause | 199,645 (7.8) | 49,514 (9.4) | 249,159 (8.1) |  | 156,523 (7.6) | 41,759 (9.4) | 198,282 (7.9) |  | 139,981 (7.6) | 41,229 (9.4) | 181,210 (8.0) |
|  |  |  |  |  |  |  |  |  |  |  |  |

^1^Matched sets including one exposed patient and at least one unexposed patient.

GUS: Genitourinary system

# **Table E18**. Association between eczema and all-cause and cause-specific mortality (cause-specific hazards). Fitted to individuals with complete data for all variables included in the models and from valid matched sets^1^. n = 2,278,944.

|  |  |  |  | HR & 99% CI^2^ | | | |
| --- | --- | --- | --- | --- | --- | --- | --- |
|  | n | P-Y at risk | Events | Adjusted | | Mediation^3^ | |
| **All-cause mortality** |  |  |  |  |  |  |  |
|  |  |  |  |  |  |  |  |
| All-cause mortality |  |  |  |  |  |  |  |
| Unexposed | 1,838,627 | 11,579,809 | 139,981 | 1.00 | (ref) | 1.00 | (ref) |
| Exposed | 440,317 | 2,949,283 | 41,229 | 1.07 | 1.05, 1.08 | 1.04 | 1.02, 1.06 |
|  |  |  |  |  |  |  |  |
| **Cause-specific mortality** |  |  |  |  |  |  |  |
|  |  |  |  |  |  |  |  |
| Infections |  |  |  |  |  |  |  |
| Unexposed | 1,838,627 | 11,579,809 | 1,493 | 1.00 | (ref) | 1.00 | (ref) |
| Exposed | 440,317 | 2,949,283 | 480 | 1.16 | 0.98, 1.36 | 1.10 | 0.92, 1.30 |
|  |  |  |  |  |  |  |  |
| Neoplasms |  |  |  |  |  |  |  |
| Unexposed | 1,838,627 | 11,579,809 | 44,567 | 1.00 | (ref) | 1.00 | (ref) |
| Exposed | 440,317 | 2,949,283 | 12,404 | 1.06 | 1.03, 1.09 | 1.03 | 1.00, 1.07 |
|  |  |  |  |  |  |  |  |
| Circulatory disease |  |  |  |  |  |  |  |
| Unexposed | 1,838,627 | 11,579,809 | 47,254 | 1.00 | (ref) | 1.00 | (ref) |
| Exposed | 440,317 | 2,949,283 | 13,887 | 1.06 | 1.03, 1.09 | 1.03 | 1.00, 1.07 |
|  |  |  |  |  |  |  |  |
| Respiratory disease |  |  |  |  |  |  |  |
| Unexposed | 1,838,627 | 11,579,809 | 19,062 | 1.00 | (ref) | 1.00 | (ref) |
| Exposed | 440,317 | 2,949,283 | 6,243 | 1.09 | 1.04, 1.15 | 1.07 | 1.02, 1.13 |
|  |  |  |  |  |  |  |  |
| Digestive disease |  |  |  |  |  |  |  |
| Unexposed | 1,838,627 | 11,579,809 | 6,788 | 1.00 | (ref) | 1.00 | (ref) |
| Exposed | 440,317 | 2,949,283 | 2,087 | 1.15 | 1.07, 1.24 | 1.08 | 0.99, 1.17 |
|  |  |  |  |  |  |  |  |
| Diseases of the GUS |  |  |  |  |  |  |  |
| Unexposed | 1,838,627 | 11,579,809 | 2,535 | 1.00 | (ref) | 1.00 | (ref) |
| Exposed | 440,317 | 2,949,283 | 818 | 1.11 | 0.97, 1.27 | 1.08 | 0.95, 1.24 |
|  |  |  |  |  |  |  |  |
| Other causes |  |  |  |  |  |  |  |
| Unexposed | 1,838,627 | 11,579,809 | 18,282 | 1.00 | (ref) | 1.00 | (ref) |
| Exposed | 440,317 | 2,949,283 | 5,310 | 1.05 | 1.00, 1.10 | 1.03 | 0.98, 1.09 |
|  |  |  |  |  |  |  |  |

^1^Matched sets including one exposed patient and at least one unexposed patient.

^2^Estimated hazard ratios from Cox regression with current age as underlying timescale, stratified by matched set (matched on age at cohort entry, gender, date at cohort entry and practice).

^3^Exploratory analysis additionally adjusting for potential mediators.

GUS: Genitourinary system

Adjusted: Adjusted for current calendar period (1998-2001, 2002-2004, 2005-2007, 2008-2010, 2011-2013, 2014-2016), Index of Multiple Deprivation at cohort entry and time-varying asthma.

Mediation: Adjusted additionally for BMI and smoking at cohort entry, and time-varying depression, anxiety, diabetes and harmful alcohol use.

# **Table E19**. Association between atopic eczema severity and all-cause and cause-specific mortality (cause-specific hazards). Fitted to individuals with complete data for all variables included in the models and from valid matched sets^1^. n = 2,278,944.

|  |  |  |  | HR & 99% CI^2^ | | | |
| --- | --- | --- | --- | --- | --- | --- | --- |
|  | n | P-Y at risk | Events | Adjusted | | Mediation^3^ | |
| **All-cause mortality** |  |  |  |  |  |  |  |
|  |  |  |  |  |  |  |  |
| All-cause mortality |  |  |  |  |  |  |  |
| Unexposed | 1,838,627 | 11,579,809 | 139,981 | 1.00 | (ref) | 1.00 | (ref) |
| Exposed – low | 318,935 | 1,754,038 | 19,919 | 1.00 | 0.98, 1.03 | 0.99 | 0.97, 1.02 |
| Exposed – moderate | 163,834 | 1,025,126 | 17,595 | 1.06 | 1.03, 1.09 | 1.02 | 0.99, 1.05 |
| Exposed – severe | 29,715 | 170,118 | 3,715 | 1.64 | 1.54, 1.74 | 1.55 | 1.46, 1.65 |
|  |  |  |  |  |  |  |  |
| **Cause-specific mortality** |  |  |  |  |  |  |  |
|  |  |  |  |  |  |  |  |
| Infections |  |  |  |  |  |  |  |
| Unexposed | 1,838,627 | 11,579,809 | 1,493 | 1.00 | (ref) | 1.00 | (ref) |
| Exposed – low | 318,935 | 1,754,038 | 208 | 0.96 | 0.76, 1.22 | 0.91 | 0.71, 1.16 |
| Exposed – moderate | 163,834 | 1,025,126 | 206 | 1.18 | 0.91, 1.52 | 1.13 | 0.86, 1.47 |
| Exposed – severe | 29,715 | 170,118 | 66 | 2.86 | 1.70, 4.79 | 2.61 | 1.52, 4.49 |
|  |  |  |  |  |  |  |  |
| Neoplasms |  |  |  |  |  |  |  |
| Unexposed | 1,838,627 | 11,579,809 | 44,567 | 1.00 | (ref) | 1.00 | (ref) |
| Exposed – low | 318,935 | 1,754,038 | 6,525 | 1.07 | 1.03, 1.12 | 1.06 | 1.02, 1.11 |
| Exposed – moderate | 163,834 | 1,025,126 | 5,005 | 1.01 | 0.96, 1.06 | 0.98 | 0.93, 1.02 |
| Exposed – severe | 29,715 | 170,118 | 874 | 1.23 | 1.09, 1.38 | 1.17 | 1.04, 1.32 |
|  |  |  |  |  |  |  |  |
| Circulatory disease |  |  |  |  |  |  |  |
| Unexposed | 1,838,627 | 11,579,809 | 47,254 | 1.00 | (ref) | 1.00 | (ref) |
| Exposed – low | 318,935 | 1,754,038 | 6,490 | 0.97 | 0.92, 1.01 | 0.96 | 0.92, 1.00 |
| Exposed – moderate | 163,834 | 1,025,126 | 6,209 | 1.10 | 1.05, 1.15 | 1.06 | 1.01, 1.11 |
| Exposed – severe | 29,715 | 170,118 | 1,188 | 1.54 | 1.38, 1.72 | 1.47 | 1.32, 1.65 |
|  |  |  |  |  |  |  |  |
| Respiratory disease |  |  |  |  |  |  |  |
| Unexposed | 1,838,627 | 11,579,809 | 19,062 | 1.00 | (ref) | 1.00 | (ref) |
| Exposed – low | 318,935 | 1,754,038 | 2,846 | 0.96 | 0.89, 1.03 | 0.95 | 0.88, 1.02 |
| Exposed – moderate | 163,834 | 1,025,126 | 2,678 | 1.09 | 1.01, 1.17 | 1.06 | 0.98, 1.14 |
| Exposed – severe | 29,715 | 170,118 | 719 | 2.35 | 2.00, 2.77 | 2.29 | 1.93, 2.72 |
|  |  |  |  |  |  |  |  |
| Digestive disease |  |  |  |  |  |  |  |
| Unexposed | 1,838,627 | 11,579,809 | 6,788 | 1.00 | (ref) | 1.00 | (ref) |
| Exposed – low | 318,935 | 1,754,038 | 956 | 1.00 | 0.90, 1.12 | 0.95 | 0.84, 1.08 |
| Exposed – moderate | 163,834 | 1,025,126 | 915 | 1.21 | 1.08, 1.36 | 1.10 | 0.97, 1.25 |
| Exposed – severe | 29,715 | 170,118 | 216 | 1.99 | 1.54, 2.57 | 1.90 | 1.43, 2.52 |
|  |  |  |  |  |  |  |  |
| Diseases of the GUS |  |  |  |  |  |  |  |
| Unexposed | 1,838,627 | 11,579,809 | 2,535 | 1.00 | (ref) | 1.00 | (ref) |
| Exposed – low | 318,935 | 1,754,038 | 342 | 0.94 | 0.78, 1.15 | 0.95 | 0.77, 1.16 |
| Exposed – moderate | 163,834 | 1,025,126 | 374 | 1.16 | 0.95, 1.41 | 1.13 | 0.92, 1.38 |
| Exposed – severe | 29,715 | 170,118 | 102 | 2.21 | 1.43, 3.41 | 1.89 | 1.20, 2.98 |
|  |  |  |  |  |  |  |  |
| Other causes |  |  |  |  |  |  |  |
| Unexposed | 1,838,627 | 11,579,809 | 18,282 | 1.00 | (ref) | 1.00 | (ref) |
| Exposed – low | 318,935 | 1,754,038 | 2,552 | 0.97 | 0.90, 1.04 | 0.96 | 0.90, 1.04 |
| Exposed – moderate | 163,834 | 1,025,126 | 2,208 | 1.01 | 0.93, 1.09 | 0.98 | 0.90, 1.06 |
| Exposed – severe | 29,715 | 170,118 | 550 | 2.12 | 1.79, 2.51 | 2.02 | 1.69, 2.41 |
|  |  |  |  |  |  |  |  |

^1^Matched sets including one exposed patient and at least one unexposed patient.

^2^Estimated hazard ratios from Cox regression with current age as underlying timescale, stratified by matched set (matched on age at cohort entry, gender, date at cohort entry and practice).

^3^Exploratory analysis additionally adjusting for potential mediators.

GUS: Genitourinary system

Adjusted: Adjusted for current calendar period (1998-2001, 2002-2004, 2005-2007, 2008-2010, 2011-2013, 2014-2016), Index of Multiple Deprivation at cohort entry and time-varying asthma.

Mediation: Adjusted additionally for BMI and smoking at cohort entry, and time-varying depression, anxiety, diabetes and harmful alcohol use.

# **Table E20**. Association between atopic eczema activity and all-cause and cause-specific mortality (cause-specific hazards), by activity of atopic eczema (assessed during the first twelve months of follow-up, assuming active eczema for three months after a single health care contact), excluding the first 12 months of follow-up. Fitted to individuals with complete data for all variables included in the models and from valid matched sets^1^. n = 1,882,012.

|  |  |  |  | HR & 99% CI^2^ | | | |
| --- | --- | --- | --- | --- | --- | --- | --- |
|  | n | P-Y at risk | Events | Adjusted | | Mediation^3^ | |
| **All-cause mortality** |  |  |  |  |  |  |  |
|  |  |  |  |  |  |  |  |
| All-cause mortality |  |  |  |  |  |  |  |
| Unexposed | 1,495,905 | 9,406,298 | 115,874 | 1.00 | (ref) | 1.00 | (ref) |
| Exposed – never active | 131,987 | 898,689 | 10,868 | 0.99 | 0.96, 1.03 | 0.98 | 0.94, 1.01 |
| Exposed – <50% active | 179,355 | 1,167,831 | 13,635 | 0.98 | 0.95, 1.01 | 0.96 | 0.93, 0.99 |
| Exposed – 50+% active | 74,765 | 460,998 | 11,515 | 1.27 | 1.23, 1.31 | 1.21 | 1.17, 1.25 |
|  |  |  |  |  |  |  |  |
| **Cause-specific mortality** |  |  |  |  |  |  |  |
|  |  |  |  |  |  |  |  |
| Infections |  |  |  |  |  |  |  |
| Unexposed | 1,495,905 | 9,406,298 | 1,259 | 1.00 | (ref) | 1.00 | (ref) |
| Exposed – never active | 131,987 | 898,689 | 114 | 0.88 | 0.64, 1.22 | 0.88 | 0.63, 1.23 |
| Exposed – <50% active | 179,355 | 1,167,831 | 153 | 1.09 | 0.82, 1.44 | 1.01 | 0.75, 1.36 |
| Exposed – 50+% active | 74,765 | 460,998 | 159 | 1.54 | 1.14, 2.07 | 1.41 | 1.03, 1.92 |
|  |  |  |  |  |  |  |  |
| Neoplasms |  |  |  |  |  |  |  |
| Unexposed | 1,495,905 | 9,406,298 | 37,570 | 1.00 | (ref) | 1.00 | (ref) |
| Exposed – never active | 131,987 | 898,689 | 3,323 | 1.00 | 0.95, 1.06 | 0.98 | 0.93, 1.05 |
| Exposed – <50% active | 179,355 | 1,167,831 | 4,444 | 1.00 | 0.96, 1.05 | 0.99 | 0.94, 1.04 |
| Exposed – 50+% active | 74,765 | 460,998 | 2,898 | 1.10 | 1.03, 1.17 | 1.06 | 0.99, 1.13 |
|  |  |  |  |  |  |  |  |
| Circulatory disease |  |  |  |  |  |  |  |
| Unexposed | 1,495,905 | 9,406,298 | 38,774 | 1.00 | (ref) | 1.00 | (ref) |
| Exposed – never active | 131,987 | 898,689 | 3,741 | 1.03 | 0.97, 1.09 | 1.01 | 0.95, 1.07 |
| Exposed – <50% active | 179,355 | 1,167,831 | 4,519 | 0.97 | 0.92, 1.02 | 0.96 | 0.91, 1.01 |
| Exposed – 50+% active | 74,765 | 460,998 | 3,918 | 1.27 | 1.20, 1.34 | 1.20 | 1.13, 1.28 |
|  |  |  |  |  |  |  |  |
| Respiratory disease |  |  |  |  |  |  |  |
| Unexposed | 1,495,905 | 9,406,298 | 15,699 | 1.00 | (ref) | 1.00 | (ref) |
| Exposed – never active | 131,987 | 898,689 | 1,518 | 0.92 | 0.83, 1.02 | 0.91 | 0.82, 1.02 |
| Exposed – <50% active | 179,355 | 1,167,831 | 1,927 | 0.98 | 0.90, 1.06 | 0.95 | 0.87, 1.04 |
| Exposed – 50+% active | 74,765 | 460,998 | 2,110 | 1.48 | 1.36, 1.62 | 1.43 | 1.31, 1.57 |
|  |  |  |  |  |  |  |  |
| Digestive disease |  |  |  |  |  |  |  |
| Unexposed | 1,495,905 | 9,406,298 | 5,675 | 1.00 | (ref) | 1.00 | (ref) |
| Exposed – never active | 131,987 | 898,689 | 576 | 1.04 | 0.90, 1.21 | 0.97 | 0.82, 1.14 |
| Exposed – <50% active | 179,355 | 1,167,831 | 697 | 1.05 | 0.93, 1.20 | 1.00 | 0.87, 1.16 |
| Exposed – 50+% active | 74,765 | 460,998 | 564 | 1.38 | 1.19, 1.61 | 1.24 | 1.05, 1.46 |
|  |  |  |  |  |  |  |  |
| Diseases of the GUS |  |  |  |  |  |  |  |
| Unexposed | 1,495,905 | 9,406,298 | 2,110 | 1.00 | (ref) | 1.00 | (ref) |
| Exposed – never active | 131,987 | 898,689 | 211 | 1.03 | 0.79, 1.35 | 1.06 | 0.80, 1.39 |
| Exposed – <50% active | 179,355 | 1,167,831 | 252 | 0.88 | 0.70, 1.11 | 0.84 | 0.66, 1.07 |
| Exposed – 50+% active | 74,765 | 460,998 | 273 | 1.60 | 1.27, 2.02 | 1.51 | 1.18, 1.93 |
|  |  |  |  |  |  |  |  |
| Other causes |  |  |  |  |  |  |  |
| Unexposed | 1,495,905 | 9,406,298 | 14,787 | 1.00 | (ref) | 1.00 | (ref) |
| Exposed – never active | 131,987 | 898,689 | 1,385 | 0.96 | 0.87, 1.06 | 0.93 | 0.84, 1.03 |
| Exposed – <50% active | 179,355 | 1,167,831 | 1,643 | 0.91 | 0.84, 1.00 | 0.91 | 0.83, 1.00 |
| Exposed – 50+% active | 74,765 | 460,998 | 1,593 | 1.38 | 1.25, 1.52 | 1.32 | 1.19, 1.46 |
|  |  |  |  |  |  |  |  |

^1^Matched sets including one exposed patient and at least one unexposed patient.

^2^Estimated hazard ratios from Cox regression with current age as underlying timescale, stratified by matched set (matched on age at cohort entry, gender, date at cohort entry and practice).

^3^Exploratory analysis additionally adjusting for potential mediators.

GUS: Genitourinary system

Unadjusted: No adjustment.

Adjusted: Adjusted for current calendar period (1998-2001, 2002-2004, 2005-2007, 2008-2010, 2011-2013, 2014-2016), Index of Multiple Deprivation at cohort entry and time-varying asthma.

Mediation: Adjusted additionally for BMI and smoking at cohort entry, and time-varying depression, anxiety, diabetes and harmful alcohol use.

# **Table E21**. Summary statistics comparing people with registrations from 2006 onwards with the overall cohort.

|  | Complete data for mediation analysis variables  & from valid matched set^1^ | | |  | Registration 2006 onwards, complete data for  mediation analysis variables & from valid matched set^1^ | | |
| --- | --- | --- | --- | --- | --- | --- | --- |
|  | Without  atopic eczema  n = 1,838,627  (80.7%) | With atopic  eczema  n = 440,317  (19.3%) | Total  n = 2,278,944 |  | Without  atopic eczema  n = 228,548  (75.8%) | With atopic  eczema  n = 73,145  (24.2%) | Total  n = 301,693 |
| Follow-up (years) |  |  |  |  |  |  |  |
| Median | 5.0 | 5.5 | 5.1 |  | 1.7 | 2.0 | 1.8 |
| Quartiles | 2.0, 9.7 | 2.3, 10.3 | 2.1, 9.8 |  | 0.7, 3.3 | 0.9, 3.7 | 0.8, 3.4 |
|  |  |  |  |  |  |  |  |
| **At entry to cohort** |  |  |  |  |  |  |  |
| Gender |  |  |  |  |  |  |  |
| Male | 677,591 (36.9) | 167,748 (38.1) | 845,339 (37.1) |  | 68,074 (29.8) | 25,230 (34.5) | 93,304 (30.9) |
| Female | 1,161,036 (63.1) | 272,569 (61.9) | 1,433,605 (62.9) |  | 160,474 (70.2) | 47,915 (65.5) | 208,389 (69.1) |
|  |  |  |  |  |  |  |  |
| Age (years) |  |  |  |  |  |  |  |
| 18-19 | 91,676 (5.0) | 38,455 (8.7) | 130,131 (5.7) |  | 5,970 (2.6) | 3,044 (4.2) | 9,014 (3.0) |
| 20-29 | 295,171 (16.1) | 69,635 (15.8) | 364,806 (16.0) |  | 86,377 (37.8) | 25,271 (34.5) | 111,648 (37.0) |
| 30-39 | 333,430 (18.1) | 73,541 (16.7) | 406,971 (17.9) |  | 65,724 (28.8) | 17,771 (24.3) | 83,495 (27.7) |
| 40-49 | 294,373 (16.0) | 65,371 (14.8) | 359,744 (15.8) |  | 28,622 (12.5) | 9,927 (13.6) | 38,549 (12.8) |
| 50-59 | 285,382 (15.5) | 60,888 (13.8) | 346,270 (15.2) |  | 15,605 (6.8) | 6,036 (8.3) | 21,641 (7.2) |
| 60-69 | 268,197 (14.6) | 59,184 (13.4) | 327,381 (14.4) |  | 12,905 (5.6) | 5,242 (7.2) | 18,147 (6.0) |
| 70-79 | 185,709 (10.1) | 47,862 (10.9) | 233,571 (10.2) |  | 7,373 (3.2) | 3,229 (4.4) | 10,602 (3.5) |
| 80+ | 84,689 (4.6) | 25,381 (5.8) | 110,070 (4.8) |  | 5,972 (2.6) | 2,625 (3.6) | 8,597 (2.8) |
|  |  |  |  |  |  |  |  |
| IMD |  |  |  |  |  |  |  |
| 1 (Least deprived) | 440,206 (23.9) | 105,492 (24.0) | 545,698 (23.9) |  | 52,141 (22.8) | 16,922 (23.1) | 69,063 (22.9) |
| 2 | 423,456 (23.0) | 101,188 (23.0) | 524,644 (23.0) |  | 49,267 (21.6) | 16,036 (21.9) | 65,303 (21.6) |
| 3 | 362,701 (19.7) | 86,239 (19.6) | 448,940 (19.7) |  | 43,835 (19.2) | 14,164 (19.4) | 57,999 (19.2) |
| 4 | 350,529 (19.1) | 84,365 (19.2) | 434,894 (19.1) |  | 46,353 (20.3) | 14,718 (20.1) | 61,071 (20.2) |
| 5 (Most deprived) | 261,735 (14.2) | 63,033 (14.3) | 324,768 (14.3) |  | 36,952 (16.2) | 11,305 (15.5) | 48,257 (16.0) |
|  |  |  |  |  |  |  |  |
| BMI |  |  |  |  |  |  |  |
| Underweight | 53,392 (2.9) | 12,950 (2.9) | 66,342 (2.9) |  | 8,706 (3.8) | 2,362 (3.2) | 11,068 (3.7) |
| Normal weight | 827,011 (45.0) | 193,656 (44.0) | 1,020,667 (44.8) |  | 112,326 (49.1) | 33,518 (45.8) | 145,844 (48.3) |
| Overweight | 601,001 (32.7) | 142,283 (32.3) | 743,284 (32.6) |  | 64,792 (28.3) | 21,601 (29.5) | 86,393 (28.6) |
| Obese | 357,223 (19.4) | 91,428 (20.8) | 448,651 (19.7) |  | 42,724 (18.7) | 15,664 (21.4) | 58,388 (19.4) |
|  |  |  |  |  |  |  |  |
| Smoking |  |  |  |  |  |  |  |
| Non-smoker | 952,392 (51.8) | 220,050 (50.0) | 1,172,442 (51.4) |  | 113,953 (49.9) | 33,718 (46.1) | 147,671 (48.9) |
| Current/ex-smoker | 886,235 (48.2) | 220,267 (50.0) | 1,106,502 (48.6) |  | 114,595 (50.1) | 39,427 (53.9) | 154,022 (51.1) |
|  |  |  |  |  |  |  |  |
| Diabetes | 110,491 (6.0) | 30,660 (7.0) | 141,151 (6.2) |  | 10,735 (4.7) | 4,630 (6.3) | 15,365 (5.1) |
| Depression | 42,893 (2.3) | 13,973 (3.2) | 56,866 (2.5) |  | 7,098 (3.1) | 3,267 (4.5) | 10,365 (3.4) |
| Anxiety | 15,203 (0.8) | 5,451 (1.2) | 20,654 (0.9) |  | 1,516 (0.7) | 842 (1.2) | 2,358 (0.8) |
| Asthma | 228,203 (12.4) | 102,909 (23.4) | 331,112 (14.5) |  | 32,172 (14.1) | 21,542 (29.5) | 53,714 (17.8) |
| Harmful alcohol use | 46,618 (2.5) | 13,149 (3.0) | 59,767 (2.6) |  | 8,604 (3.8) | 3,497 (4.8) | 12,101 (4.0) |
|  |  |  |  |  |  |  |  |
| **By exit from cohort** |  |  |  |  |  |  |  |
| Diabetes | 204,524 (11.1) | 57,140 (13.0) | 261,664 (11.5) |  | 14,044 (6.1) | 6,159 (8.4) | 20,203 (6.7) |
| Depression | 87,202 (4.7) | 28,440 (6.5) | 115,642 (5.1) |  | 10,145 (4.4) | 4,635 (6.3) | 14,780 (4.9) |
| Anxiety | 27,296 (1.5) | 9,232 (2.1) | 36,528 (1.6) |  | 2,270 (1.0) | 1,185 (1.6) | 3,455 (1.1) |
| Asthma | 285,677 (15.5) | 122,039 (27.7) | 407,716 (17.9) |  | 34,554 (15.1) | 22,763 (31.1) | 57,317 (19.0) |
| Harmful alcohol use | 70,543 (3.8) | 19,979 (4.5) | 90,522 (4.0) |  | 9,858 (4.3) | 4,056 (5.5) | 13,914 (4.6) |
|  |  |  |  |  |  |  |  |

^1^Matched sets including one exposed person and at least one unexposed person.

# **Table E22**. Association between atopic eczema and all-cause and cause-specific mortality (cause-specific hazards) restricted to people registered from 2006 onwards. Fitted to individuals with complete data for all variables included in the models and from valid matched sets^1^. n = 301,693.

|  |  |  |  | HR & 99% CI^2^ | | | |
| --- | --- | --- | --- | --- | --- | --- | --- |
|  | n | P-Y at risk | Events | Adjusted | | Mediation^3^ | |
| **All-cause mortality** |  |  |  |  |  |  |  |
|  |  |  |  |  |  |  |  |
| All-cause mortality |  |  |  |  |  |  |  |
| Unexposed | 228,548 | 516,574 | 3,097 | 1.00 | (ref) | 1.00 | (ref) |
| Exposed | 73,145 | 183,314 | 1,534 | 1.08 | 0.95, 1.22 | 1.06 | 0.93, 1.20 |
|  |  |  |  |  |  |  |  |
| **Cause-specific mortality** |  |  |  |  |  |  |  |
|  |  |  |  |  |  |  |  |
| Infections |  |  |  |  |  |  |  |
| Unexposed | 228,548 | 516,574 | 32 | 1.00 | (ref) | 1.00 | (ref) |
| Exposed | 73,145 | 183,314 | 15 | 0.70 | 0.18, 2.74 | 0.38 | 0.05, 2.88 |
|  |  |  |  |  |  |  |  |
| Neoplasms |  |  |  |  |  |  |  |
| Unexposed | 228,548 | 516,574 | 687 | 1.00 | (ref) | 1.00 | (ref) |
| Exposed | 73,145 | 183,314 | 362 | 1.22 | 0.97, 1.55 | 1.18 | 0.93, 1.51 |
|  |  |  |  |  |  |  |  |
| Circulatory disease |  |  |  |  |  |  |  |
| Unexposed | 228,548 | 516,574 | 833 | 1.00 | (ref) | 1.00 | (ref) |
| Exposed | 73,145 | 183,314 | 394 | 0.99 | 0.78, 1.27 | 0.94 | 0.72, 1.21 |
|  |  |  |  |  |  |  |  |
| Respiratory disease |  |  |  |  |  |  |  |
| Unexposed | 228,548 | 516,574 | 421 | 1.00 | (ref) | 1.00 | (ref) |
| Exposed | 73,145 | 183,314 | 248 | 1.24 | 0.89, 1.74 | 1.22 | 0.83, 1.79 |
|  |  |  |  |  |  |  |  |
| Digestive disease |  |  |  |  |  |  |  |
| Unexposed | 228,548 | 516,574 | 135 | 1.00 | (ref) | 1.00 | (ref) |
| Exposed | 73,145 | 183,314 | 81 | 1.66 | 0.97, 2.83 | 1.45 | 0.72, 2.92 |
|  |  |  |  |  |  |  |  |
| Diseases of the GUS |  |  |  |  |  |  |  |
| Unexposed | 228,548 | 516,574 | 54 | 1.00 | (ref) | 1.00 | (ref) |
| Exposed | 73,145 | 183,314 | 25 | 1.00 | 0.34, 2.96 | 0.83 | 0.23, 2.94 |
|  |  |  |  |  |  |  |  |
| Other causes |  |  |  |  |  |  |  |
| Unexposed | 228,548 | 516,574 | 935 | 1.00 | (ref) | 1.00 | (ref) |
| Exposed | 73,145 | 183,314 | 409 | 0.90 | 0.70, 1.15 | 0.96 | 0.74, 1.25 |
|  |  |  |  |  |  |  |  |

^1^Matched sets including one exposed person and at least one unexposed person.

^2^Estimated hazard ratios from Cox regression with current age as underlying timescale, stratified by matched set (matched on age at cohort entry, gender, date at cohort entry and practice).

^3^Exploratory analysis additionally adjusting for potential mediators.

GUS: Genitourinary system

Adjusted: Adjusted for current calendar period (2006-2010, 2011-2013, 2014-2016), Index of Multiple Deprivation at cohort entry and time-varying asthma.

Mediation: Adjusted additionally for BMI and smoking at cohort entry, and time-varying depression, anxiety, diabetes and harmful alcohol use.

# **Table E23**. Association between atopic eczema and all-cause and cause-specific mortality (cause-specific hazards), additionally adjusted for ever systemic drug or high-dose oral glucocorticoid use. Fitted to individuals with complete data for all variables included in the models and from valid matched sets^1^. n = 2,278,944.

|  |  |  |  | HR & 99% CI^2^ | | | |
| --- | --- | --- | --- | --- | --- | --- | --- |
|  |  |  |  | Mediation^3^ | | | |
|  | n | P-Y at risk | Events | + systemic | | + HDOGC | |
| **All-cause mortality** |  |  |  |  |  |  |  |
|  |  |  |  |  |  |  |  |
| All-cause mortality |  |  |  |  |  |  |  |
| Unexposed | 1,838,627 | 11,579,809 | 139,981 | 1.00 | (ref) | ^4^ |  |
| Exposed | 440,317 | 2,949,283 | 41,229 | 1.03 | 1.01, 1.05 |  |  |
|  |  |  |  |  |  |  |  |
| **Cause-specific mortality** |  |  |  |  |  |  |  |
|  |  |  |  |  |  |  |  |
| Infections |  |  |  |  |  |  |  |
| Unexposed | 1,838,627 | 11,579,809 | 1,493 | 1.00 | (ref) | 1.00 | (ref) |
| Exposed | 440,317 | 2,949,283 | 480 | 1.07 | 0.90, 1.27 | 1.05 | 0.88, 1.24 |
|  |  |  |  |  |  |  |  |
| Neoplasms |  |  |  |  |  |  |  |
| Unexposed | 1,838,627 | 11,579,809 | 44,567 | 1.00 | (ref) | 1.00 | (ref) |
| Exposed | 440,317 | 2,949,283 | 12,404 | 1.03 | 1.00, 1.06 | 0.95 | 0.92, 0.98 |
|  |  |  |  |  |  |  |  |
| Circulatory disease |  |  |  |  |  |  |  |
| Unexposed | 1,838,627 | 11,579,809 | 47,254 | 1.00 | (ref) | ^4^ |  |
| Exposed | 440,317 | 2,949,283 | 13,887 | 1.03 | 1.00, 1.06 |  |  |
|  |  |  |  |  |  |  |  |
| Respiratory disease |  |  |  |  |  |  |  |
| Unexposed | 1,838,627 | 11,579,809 | 19,062 | 1.00 | (ref) | ^4^ |  |
| Exposed | 440,317 | 2,949,283 | 6,243 | 1.05 | 1.00, 1.11 |  |  |
|  |  |  |  |  |  |  |  |
| Digestive disease |  |  |  |  |  |  |  |
| Unexposed | 1,838,627 | 11,579,809 | 6,788 | 1.00 | (ref) | 1.00 | (ref) |
| Exposed | 440,317 | 2,949,283 | 2,087 | 1.06 | 0.98, 1.16 | 1.05 | 0.97, 1.15 |
|  |  |  |  |  |  |  |  |
| Diseases of the GUS |  |  |  |  |  |  |  |
| Unexposed | 1,838,627 | 11,579,809 | 2,535 | 1.00 | (ref) | ^4^ |  |
| Exposed | 440,317 | 2,949,283 | 818 | 1.07 | 0.94, 1.23 |  |  |
|  |  |  |  |  |  |  |  |
| Other causes |  |  |  |  |  |  |  |
| Unexposed | 1,838,627 | 11,579,809 | 18,282 | 1.00 | (ref) | ^4^ |  |
| Exposed | 440,317 | 2,949,283 | 5,310 | 1.02 | 0.97, 1.07 |  |  |
|  |  |  |  |  |  |  |  |

^1^Matched sets including one exposed person and at least one unexposed person.

^2^Estimated hazard ratios from Cox regression with current age as underlying timescale, stratified by matched set (matched on age at cohort entry, gender, date at cohort entry and practice).

^3^Exploratory analysis additionally adjusting for potential mediators.

^4^Ever high-dose oral glucocorticoid use results not reported as hypothesised mechanism for these causes of death is only via current high-dose oral glucocorticoid use (see Table S18).

Systemic: Systemic drug use (ever, time-updated variable).

HDOGS: High-dose oral glucocorticoid use (ever, time-updated variable).

GUS: Genitourinary system

Adjusted: Adjusted for current calendar period (1998-2001, 2002-2004, 2005-2007, 2008-2010, 2011-2013, 2014-2016), Index of Multiple Deprivation at cohort entry and time-varying asthma.

Mediation: Adjusted additionally for BMI and smoking at cohort entry, and time-varying depression, anxiety, diabetes and harmful alcohol use.

# **Table E24**. Association between atopic eczema and all-cause and cause-specific mortality (cause-specific hazards), additionally adjusted for current high-dose oral glucocorticoid use. Fitted to individuals with complete data for all variables included in the models and from valid matched sets^1^. n = 2,278,944.

|  |  |  |  | HR & 99% CI^2^ | |
| --- | --- | --- | --- | --- | --- |
|  |  |  |  | Mediation^3^ | |
|  | n | P-Y at risk | Events | + HDOGC | |
| **All-cause mortality** |  |  |  |  |  |
|  |  |  |  |  |  |
| All-cause mortality |  |  |  |  |  |
| Unexposed | 1,838,627 | 11,579,809 | 139,981 | 1.00 | (ref) |
| Exposed | 440,317 | 2,949,283 | 41,229 | 1.03 | 1.01, 1.05 |
|  |  |  |  |  |  |
| **Cause-specific mortality** |  |  |  |  |  |
|  |  |  |  |  |  |
| Infections |  |  |  |  |  |
| Unexposed | 1,838,627 | 11,579,809 | 1,493 | 1.00 | (ref) |
| Exposed | 440,317 | 2,949,283 | 480 | 1.08 | 0.91, 1.28 |
|  |  |  |  |  |  |
| Neoplasms |  |  |  |  |  |
| Unexposed | 1,838,627 | 11,579,809 | 44,567 | ^4^ |  |
| Exposed | 440,317 | 2,949,283 | 12,404 |  |  |
|  |  |  |  |  |  |
| Circulatory disease |  |  |  |  |  |
| Unexposed | 1,838,627 | 11,579,809 | 47,254 | 1.00 | (ref) |
| Exposed | 440,317 | 2,949,283 | 13,887 | 1.03 | 1.00, 1.06 |
|  |  |  |  |  |  |
| Respiratory disease |  |  |  |  |  |
| Unexposed | 1,838,627 | 11,579,809 | 19,062 | 1.00 | (ref) |
| Exposed | 440,317 | 2,949,283 | 6,243 | 1.05 | 1.00, 1.11 |
|  |  |  |  |  |  |
| Digestive disease |  |  |  |  |  |
| Unexposed | 1,838,627 | 11,579,809 | 6,788 | 1.00 | (ref) |
| Exposed | 440,317 | 2,949,283 | 2,087 | 1.07 | 0.99, 1.17 |
|  |  |  |  |  |  |
| Diseases of the GUS |  |  |  |  |  |
| Unexposed | 1,838,627 | 11,579,809 | 2,535 | 1.00 | (ref) |
| Exposed | 440,317 | 2,949,283 | 818 | 1.08 | 0.94, 1.24 |
|  |  |  |  |  |  |
| Other causes |  |  |  |  |  |
| Unexposed | 1,838,627 | 11,579,809 | 18,282 | 1.00 | (ref) |
| Exposed | 440,317 | 2,949,283 | 5,310 | 1.03 | 0.98, 1.09 |
|  |  |  |  |  |  |

^1^Matched sets including one exposed person and at least one unexposed person.

^2^Estimated hazard ratios from Cox regression with current age as underlying timescale, stratified by matched set (matched on age at cohort entry, gender, date at cohort entry and practice).

HDOGS: High-dose oral glucocorticoid use (current, time-updated variable).

^3^Exploratory analysis additionally adjusting for potential mediators.

^4^Current high-dose oral glucocorticoid use results not reported as hypothesised mechanism for these causes of death is only via ever high-dose oral glucocorticoid use (see Table S17).

GUS: Genitourinary system

Adjusted: Adjusted for current calendar period (1998-2001, 2002-2004, 2005-2007, 2008-2010, 2011-2013, 2014-2016), Index of Multiple Deprivation at cohort entry and time-varying asthma.

Mediation: Adjusted additionally for BMI and smoking at cohort entry, and time-varying depression, anxiety, diabetes and harmful alcohol use.


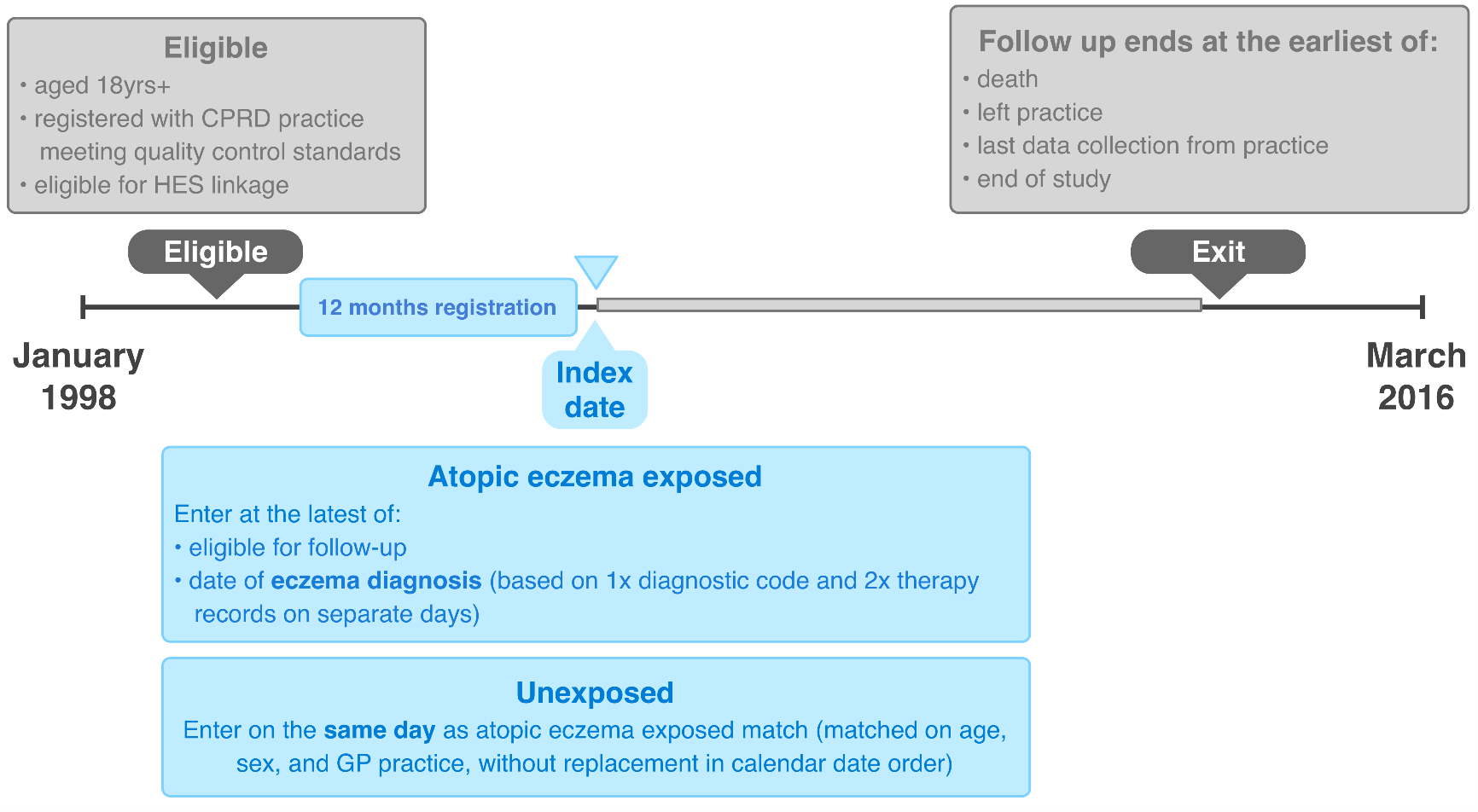


# **Fig. E1**. Graphical depiction of study design.

Diabetes

BMI

SES

Ethnicity

Age

Smoking

Atopy

Asthma

Harmful

alcohol use

Atopic

eczema

Depression/

anxiety

Mortality

Physical

activity

Gender

# **Fig. E2**. Directed acyclic graph used to inform the identification of covariates and mediators. BMI: Body mass index; SES: Socioeconomic status. Arrows between covariates have been omitted to ensure that the figure can be clearly read.

**
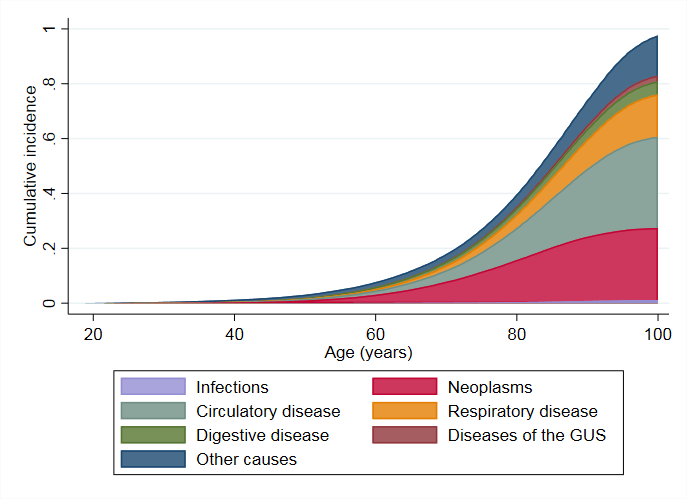
**

# **Fig. E3**. Stacked cause-specific cumulative incidence functions up to age 100 years among people with atopic eczema. Estimated non-parametrically in the full sample, allowing for competing risks. GUS: Genitourinary system.
